# De novo design of peptides localizing at the interface of biomolecular condensates

**DOI:** 10.1038/s41467-026-73099-9

**Published:** 2026-05-16

**Authors:** Timo N. Schneider, Marcos Gil-Garcia, Marco A. Bühler, Lucas F. Santos, Lenka Faltova, Gonzalo Guillén-Gosálbez, Paolo Arosio

**Affiliations:** Department of Chemistry and Applied Biosciences, Institute for Chemical and Bioengineering, ETH Zurich, Zurich, Switzerland

**Keywords:** Intrinsically disordered proteins, Computational biophysics, Machine learning

## Abstract

The interface of biomolecular condensates plays a key role in processes such as protein aggregation and biochemical reactions, making it an attractive target for engineering condensates. However, the molecular grammar that drives preferential localization to condensate interfaces remains poorly understood. Here, we develop a computational pipeline that integrates high-throughput coarse-grained simulations, machine learning, and mixed-integer linear programming to design peptides that partition at the interfaces of defined condensate targets. We validated the workflow by designing and synthesizing peptides that localize at the interface of three distinct condensates formed by intrinsically disordered protein regions. These peptides exhibit surfactant-like architectures. Specifically, one tail inserts into the condensate and is enriched in aromatic residues, while the opposite tail is excluded from the dense phase, with its sequence varying according to the scaffold’s net charge. Overall, our pipeline offers a general strategy for rationally designing interface-localizing peptides and for unraveling the governing design principles.

## Abstract (web summary)

Combining high-throughput molecular simulations, machine learning, and mixed-integer linear programming, the authors design peptides that localize to condensate interfaces, revealing surfactant-like, charge-dependent sequence rules.

## Introduction

Growing evidence shows that cells regulate biochemical reactions in space and time through the formation of membraneless organelles, also known as biomolecular condensates^1–4^. These protein- and RNA-rich condensates have been linked to several cellular functions, such as RNA metabolism and stress responses^1^, as well as disease-associated dysfunctions, including progression to arrested states and formation of amyloid fibrils^5–7^. In addition to changes in local concentrations, condensates can affect biochemical functions through other emergent properties at the mesoscale. One of these properties, which is attracting increasing attention, is the interface between the dense and dilute phases. This interface has been shown to promote the formation of disease-associated fibrils of hnRNPA1^8,9^, as well as the liquid-to-solid transition of FUS^10^. Condensate interfaces can also promote the aggregation of client proteins, as shown with mutant huntingtin polyglutamine in vivo^11^, and also *α*-synuclein in vitro^12,13^. Moreover, interfaces can define electric potentials and promote redox reactions^14–16^.

Targeted modulation of the interface of condensates, without perturbing their interior and other mesoscale properties, could therefore be an attractive strategy for engineering condensates and correcting aberrant behaviors. A traditional strategy in colloid science for altering interfacial properties involves the use of surfactant molecules. However, this approach presents challenges in the context of condensates, as the dilute and dense phases are more similar to each other than, for example, in oil-in-water emulsions^17,18^. Both phases contain substantial amounts of water and dissolved ions, with the volume fraction of water in the dense phase estimated to be around 70%^19–22^. Therefore, it is more challenging to design surfactant-like molecules that would preferentially partition at the interface of biomolecular condensates. Furthermore, interfacial localization is expected to strongly depend on the identity of the condensate.

For a set of natural and rationally designed proteins, interfacial localization has been confirmed^8,11–13,23–28^, and some condensates exhibit colloidal stability based on mechanisms reminiscent of Pickering emulsions^29^. Also, condensate interfaces have been targeted by encapsulation in lipid bilayers, formed by, for instance, fatty acids or phospholipids^30–33^. There have also been a set of engineered, potentially dendritic, polymers^34,35^, as well as peptides covalently fused to PEG polymers^36^. Generally, reducing molecular size by designing short peptides while maintaining interfacial localization is challenging, because fewer interaction sites are available per molecule. In other words, the penalty in translational entropy associated with interfacial confinement becomes increasingly difficult to offset when decreasing the size of the molecule^37^. Moreover, a high-throughput experimental or computational strategy for extensive screening is still lacking. As a result, the guiding principles for preferential localization at the interface of biomolecular condensates remain elusive.

The predictive capability of molecular dynamics simulations using coarse-grained force fields^38–41^ has shown promising results for designing polymers, peptides, and disordered proteins for a variety of applications^42–45^. The efficiency of the design process can be improved with machine learning models, enabling active learning in sequence space. The inverse design problem, i.e., selecting new sequences from a large space, guided by a trained model, is commonly tackled with genetic algorithms or by performing local optimization in a continuous, potentially learned representation of the sequence space^46–53^. One drawback of these approaches is the risk of getting trapped in local optima due to the nonconvex nature of the optimization model. Recent work^54,55^ has enabled the identification of globally optimal inputs for trained neural networks using mixed-integer linear programming (MILP), which can be used to mimic the behavior of complex systems, including nonconvex models based on first principles.

Here, we have developed a computational pipeline based on a combination of coarse-grained molecular dynamics simulations, machine learning, and MILP for the de novo design of peptides partitioning at the interface of biomolecular condensates. Specifically, coarse-grained molecular dynamics simulations were integrated with an active learning algorithm involving the training and optimization of a surrogate model based on a neural network. The optimization problem containing the trained neural network was formulated as an MILP, which led to globally optimal sequences for each iteration. We applied this framework to design de novo and experimentally validate peptides that partition at the interface of condensates formed by the intrinsically disordered domains of various proteins associated with biological condensates, such as hnRNPA1, LAF-1, and DDX4. In contrast to previous studies that generated interface-localizing molecules through often one-shot rational design, our high-throughput, simulation-based approach, augmented by active learning, enables a more thorough exploration of the design space, allowing the identification of efficient short-peptide sequences tailored to specific condensate targets. The designed peptides accumulated at the protein interface in vitro, as intended, and were also capable of modifying condensate size distribution. The obtained sequences were surfactant-like, with each case exhibiting a distinct composition. Based on these results, we identified design rules for interface-partitioning peptides, highlighting the net charge of the condensate-forming protein as a key physicochemical feature.

## Results

### Overall workflow

**Fig. 1:**
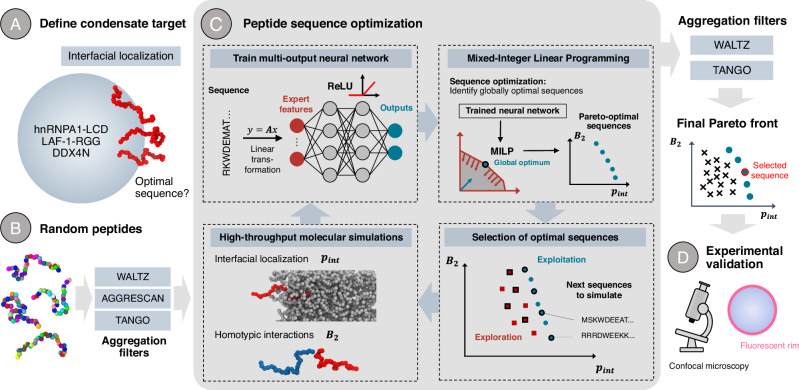
Overall workflow for designing peptides partitioning at the interface of biomolecular condensates. After the definition of a condensate target (**A**), randomly generated peptides were screened to exclude aggregation-prone regions (**B**), and entered the optimization cycle (**C**). Interface partitioning and homotypic interactions were quantified using coarse-grained molecular simulations, and the resulting data were used to train a surrogate model (neural network). In the next step, we leveraged MILP to identify globally optimal sequences, leading to new peptides to simulate. Upon convergence of this optimization cycle, we experimentally validated our predictions using confocal microscopy (**D**).

We chose to design peptides comprising 30 amino acid residues to achieve a balance between sequence variability and accessibility for chemical synthesis. The de novo design of such peptides encompasses selecting optimal sequences of amino acids from a vast design space. To address this challenge, we therefore combined high-throughput coarse-grained simulations with an active learning algorithm for a more efficient navigation. A representation of the entire workflow is shown in Fig. 1.

After defining the condensate target (Fig. 1A), the analysis began with a set of fully randomized peptides, in which each of the 20 amino acids was uniformly sampled. To enhance the synthesizability and stability of the designed peptides, we implemented a filter to prevent the emergence of any aggregation-prone regions. Specifically, we applied three different sequence-based aggregation predictors: Waltz^56^, TANGO^57^, and AGGRESCAN^58^ (Fig. 1B). While the incorporation of these filters into the computational pipeline excludes a subset of potential peptides, the large design space still allows for the design of a highly diverse set of candidates. This strategy led to 300 initial sequences, without introducing any bias towards existing biological protein sequences.

Next, the sequences were introduced into the optimization loop (Fig. 1C), where we aimed to simultaneously maximize the interface partitioning of the single peptide and minimize homotypic interactions. This is because strong self-association may lead to aggregation, reducing partitioning at the interface at higher concentrations. Moreover, the minimization of homotypic interactions serves as an additional safeguard against generating highly aggregation-prone or insoluble sequences that may not be identified by existing aggregation predictors. We performed two separate coarse-grained simulations for each peptide: in the first simulation, we quantified the probability of a single peptide to be localized at the interface of a minimalistic condensate formed by the protein of interest (*p*_int_); in the second simulation, we included two copies of the peptide to measure homotypic interactions, quantified by the second virial coefficient (*B*_2_).

In the next step, we trained a surrogate model (multi-output neural network) on the simulation results to predict both interface partitioning and homotypic interactions. The network uses engineered features, obtained through a linear transformation of the peptide sequence, as inputs, and ReLU as the activation function. Both aspects were crucial for the next step: the bi-objective optimization problem with the trained neural network embedded was reformulated as an MILP problem^54,55^ and solved to maximize simultaneously *p*_int_ and *B*_2_. This led to Pareto solutions^59^—a set of globally optimal, non-dominated sequences that cannot improve one objective function without compromising the other one—with mathematical guarantees within the massive sequence design space of size 20^30^. In contrast with, for instance, genetic algorithms, this approach avoids stagnation in local optima, enabling the construction of the true Pareto front of the surrogate model within the desired optimality gap. Combined with an exploration strategy, this process typically selects 100 new sequences for further simulations, restarting the optimization cycle. Following convergence of the cycle, we applied final aggregation filters and selected a sequence for experimental validation (Fig. 1D). A fluorescently tagged variant of the peptide was then synthesized, and its localization within the condensate system was analyzed in vitro using confocal microscopy.

### Simulation, machine learning, and optimization setup

**Fig. 2:**
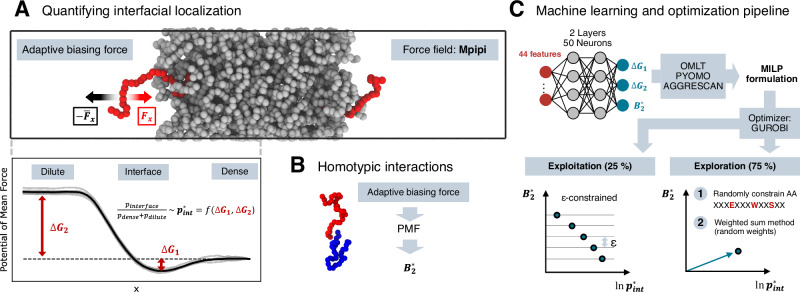
Simulation, machine learning, and optimization setup. Molecular simulations utilizing the coarse-grained force field Mpipi, in conjunction with the ABF method, were employed to quantify interfacial localization (**A**) and homotypic interactions (**B**). An ideal peptide demonstrates high interface partitioning (${p}_{\,{{{\rm{int}}}}}^{*}$), which corresponds to high values of both *ΔG*_1_ and *ΔG*_2_, while exhibiting self-repulsion, reflected by a high second virial coefficient ${B}_{2}^{*}$. A multi-output neural network was trained on the simulation results (**C**) and translated into an MILP formulation, which was then optimized with distinct strategies for exploration and exploitation.

Although the molecular simulations were performed at a coarse-grained level, computational requirements were still significant. Simulations were therefore designed to balance accuracy and throughput. We chose the Mpipi force field^39^, a one-bead per residue model, due to its physics-based parametrization strategy and ability to explain experimental data of disordered proteins. We developed a custom simulation procedure to quantify the interfacial localization of peptides, modeling the condensate as a minimalistic slab containing 16 protein copies. Using the adaptive biasing force (ABF) method^60,61^, we mapped the potential of mean force (PMF) as a function of peptide position—dilute, interface, or dense phase—as illustrated in Fig. 2A. To accelerate convergence, we employed a stratification strategy (see “Methods” section), which also enabled the inclusion of two peptides per simulation without interaction, further increasing simulation throughput. The free energy differences between the dense phase and the interface (*ΔG*_1_) and between the dense and dilute phases (*ΔG*_2_) were extracted from the PMFs and used to compute ${p}_{\,{{{\rm{int}}}}}^{*}$, our objective for interface partitioning. ${p}_{\,{{{\rm{int}}}}}^{*}$ can be approximated as proportional to the probability that the molecule localizes at the interface (*p*_interface_) relative to the bulk of the dilute and dense phase (a detailed derivation is provided in the Supplementary Information):

1 $${p}_{\,{{{\rm{int}}}}}^{*}={\left[\exp \left(-\frac{\Delta {G}_{1}}{{k}_{{{{\rm{B}}}}}T}\right)+\phi \exp \left(-\frac{\Delta {G}_{2}}{{k}_{{{{\rm{B}}}}}T}\right)\right]}^{-1} \sim \,\frac{{p}_{{{{\rm{interface}}}}}}{{p}_{{{{\rm{dense}}}}}+{p}_{{{{\rm{dilute}}}}}}$$

Here, *ϕ* represents the volume ratio between the dilute and dense phases. A peptide with high interface partitioning would exhibit large values of both *ΔG*_1_ and *ΔG*_2_.

To quantify homotypic interactions, we also used the ABF method in a separate simulation to construct the PMF between two peptide copies, employing the center of mass distance as the collective variable (Fig. 2B). The PMF was then converted into the second virial coefficient *B*_2_, a key parameter directly linked to homotypic interactions and also phase separation behavior^37,51^. To address the highly skewed distribution of values, we applied a bi-symmetric log transformation^62^, finally resulting in the second objective ${B}_{2}^{*}$. Positive values of ${B}_{2}^{*}$ indicate net repulsion, whereas negative values suggest attraction and potential colocalization of the peptide with itself.

In order to identify sequences that maximize both ${p}_{\,{{{\rm{int}}}}}^{*}$ and ${B}_{2}^{*}$, in the next step, we trained a surrogate model predicting simulation outcomes (Fig. 2C). For each design case, we typically had simulation data only for a few hundred peptide sequences. This small number prevents training a predictor directly on a sequence. Instead, we used descriptors, which have been shown to often outperform end-to-end representation learning^48,63^. We engineered a set of 44 features based on overall composition, as well as spatial distribution of charged, aromatic, and strongly self-interacting residues. Additionally, we included features connected to the sequence charge and hydropathy decoration, which play a pivotal role in governing the behavior of disordered proteins^64,65^. It is important to emphasize that all features can be derived as linear transformations of the one-hot encoded amino acid sequence, which was crucial for later steps. Based on the initial 300 simulations for our first condensate target, which was the disordered domain of hnRNPA1, we compared the predictive performance of multiple models to predict *ΔG*_1_, *ΔG*_2_, and ${B}_{2}^{*}$ (Supplementary Fig. 1). We concluded that a multi-output neural network outperformed elastic net, support vector machine, and gradient-boosted decision tree models. Two hidden layers of 50 neurons each were chosen because larger networks did not provide further improvements in predictive performance (Supplementary Fig. 1).

To address the inverse design problem of identifying optimal sequences, we employed OMLT^55^ to embed the trained neural network into the algebraic modeling framework Pyomo^66,67^. The piecewise linear nature of the ReLU activation function allowed it to be incorporated into an MILP problem using the big-M reformulation^54^. To linearize equation (1), which converts the neural network outputs *ΔG*_1_ and *ΔG*_2_ into the objective ${p}_{\,{{{\rm{int}}}}}^{*}$, we employed supporting hyperplanes (Supplementary Methods). Together, these steps enabled the formulation of the final MILP optimization problem, linking the amino acid sequence inputs to the two objectives via the trained surrogate model. Furthermore, we also used the big-M reformulation to integrate the AGGRESCAN predictor as a constraint, avoiding the emergence of any aggregation-prone regions. The other, more complicated predictors (Waltz, TANGO) were only applied at the end of the optimization cycle, as illustrated in Fig. 1. The MILP problem could be solved to global optimality using the optimizer Gurobi^68^. We utilized the *ε*-constrained method^59^ to generate a Pareto front comprising 25 optimal sequences for the next round of simulations (Fig. 2C). In the various iterations, it is important to balance exploitation with exploration. To achieve this, 75 sequences per iteration were allocated to exploration, resulting in a total of 100 sequences per iteration. For each exploration sequence, we selected between 2 and 20 positions in the peptide sequence at random and constrained them to randomly chosen amino acids. We then optimized the remaining positions using a weighted-sum approach with randomly generated weights for the two objectives, thereby removing the computational burden of computing *ε*-constraints for each exploration point. This approach aimed to strike a balance between promoting exploration and sampling potentially relevant regions of the sequence space. The explore/exploit balance, as well as the exploration strategy, were fixed a priori. Extensive optimization could potentially further increase pipeline efficiency. The entire optimization loop was stopped as soon as the hypervolume of the Pareto front stagnated.

### Peptides targeting the interface of hnRNPA1-LCD condensates

**Fig. 3:**
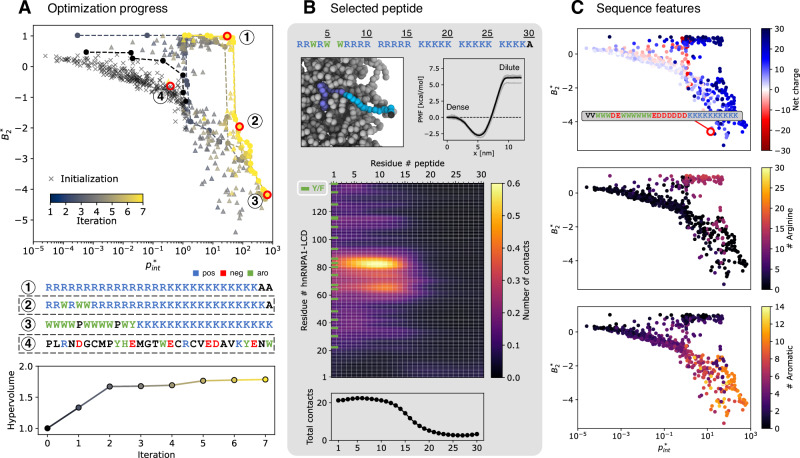
Peptide sequence optimization with hnRNPA1-LCD as the condensate target. **A** Over seven iterations, the primary objective ${p}_{\,{{{\rm{int}}}}}^{*}$, which quantifies interface partitioning, improved by approximately three orders of magnitude. Peptide 2 was later selected for experimental validation of interfacial partitioning, with peptide 4 serving as a control. **B** The selected peptide exhibits surfactant-like behavior: its arginine- and tryptophan-rich end engages in favorable *π*–*π* and cation–*π* interactions with hnRNPA1-LCD, while the positively charged polylysine tail is preferentially excluded from the also positively charged condensate phase. **C** All Pareto-optimal sequences exhibit a high net positive charge, which the algorithm identified as superior to more neutral variants containing aspartic or glutamic acid. Along the Pareto front, aromatic content increases while arginine content decreases with increasing homotypic interactions (low ${B}_{2}^{*}$).

We first applied our approach to design peptides targeting the interface of condensates formed by the low complexity domain (LCD) of hnRNPA1. The optimization pipeline successfully identified peptides exhibiting high interface partitioning and low homotypic interaction, as quantified by coarse-grained simulations. The primary objective ${p}_{\,{{{\rm{int}}}}}^{*}$, which measures interface partitioning, improved by approximately three orders of magnitude compared to the initialization (Fig. 3A). The algorithm achieved substantial advancement of the Pareto front in the first two iterations, after which the hypervolume plateaued. Optimization was stopped after seven iterations due to stagnation in hypervolume and the similarity of newly suggested sequences to those already simulated. We also compared the construction of the Pareto front using our MILP-based approach with the commonly used genetic algorithm NSGA-II^69^ for the first two iterations. Despite converging on hypervolume, the GA can fail to capture a substantial portion of the true Pareto front (Supplementary Fig. 2). Furthermore, using MILP provides a formal guarantee of convergence to the global optimum of the optimization problem with the ANN embedded, eliminating any uncertainty that can arise with a GA. This highlights the advantage of employing MILP over GA when optimizing with the trained neural network.

As optimization progressed, the Pareto-optimal peptide sequences became increasingly homogeneous in composition, containing only a subset of the 20 amino acids (Supplementary Fig. 3). This trend is quantitatively reflected in the decreasing average Shannon entropy of the optimal sequences (Supplementary Fig. 4). When sampling sequences from the Pareto front (Fig. 3A), it is visible that they exhibit a surfactant-like architecture, as well as a positive net charge. One tail of the peptides is typically enriched in tryptophans and arginines, while the other mainly consists of lysines. We selected a peptide from the center of the Pareto front for a closer investigation of the mechanism behind the interfacial localization (Fig. 3B). As indicated by the PMF, this peptide preferentially localizes in the dense rather than the dilute phase, and exhibits even greater affinity for the interface, with the free energy minimum about 2.5 kcal/mol lower than the value in the dense phase. An unbiased simulation (see “Methods” section) was run to analyze the conformational ensemble of the peptide and the per-residue contacts with hnRNPA1-LCD. It is visible that the tail rich in arginines and tryptophans heavily interacts with the dense phase formed by the disordered proteins, while the polylysine end remains largely outside the condensate, leading to overall low contact probabilities. This behavior is physically reasonable, as both tryptophan and arginine engage in strong *π*–*π* and cation–*π* interactions, which are known drivers of protein phase separation, including in hnRNPA1-LCD^70–72^. In fact, the strongest interactions, as identified by the intermolecular contact map, occur with aromatic-rich segments of hnRNPA1 (Fig. 3B). Tryptophan is likely favored by the algorithm over other aromatic residues because its interactions with both aromatics and arginine tend to be stronger, a feature that is reflected in the Mpipi force field^39^. By contrast, cation–*π* interactions involving lysine are weaker than those with arginine. Moreover, hnRNPA1-LCD has a net charge of +8, matching the positive charge of the peptide and thereby promoting the exclusion of the polylysine tail from the condensate. We performed additional simulations to investigate how interfacial composition varies with peptide concentration (see “Methods” section). Increasing peptide concentration enriched aromatic and negatively charged protein residues at the interface, consistent with the contact map and suggesting that the peptide may not only localize but also induce structural rearrangements in the interfacial region (Supplementary Figs. 5 and 6).

The positive net charge remains a consistent sequence feature across the entire Pareto front (Fig. 3C). Peptides with interaction-prone tails containing negatively charged residues, such as aspartic and glutamic acid, were also explored but proved less effective in maximizing the objective functions and were consequently dropped by the algorithm in later iterations. Along the Pareto front, as interface partitioning increases (high ${p}_{\,{{{\rm{int}}}}}^{*}$, low ${B}_{2}^{*}$), the number of arginines in the sequence decreases while the aromatic fraction increases. At the highest ${p}_{\,{{{\rm{int}}}}}^{*}$ values, sequences no longer contain arginines but are primarily composed of tryptophans and lysines. Aromatic patches are interrupted by prolines (Fig. 3A), a consequence of the AGGRESCAN constraint, which prevents the emergence of aggregation-prone regions by incorporating proline as a beta-sheet-breaking residue^73^. Notably, Fig. 3 shows the direct output of the optimization procedure before the Waltz and TANGO aggregation filters were applied. Once these additional filters are imposed, many peptides with high aromatic content were excluded (Supplementary Fig. 7). Consequently, we selected sequences 2 and 4 (Fig. 3A) for experimental validation, as both passed the additional aggregation filters and exhibit different levels of predicted interface partitioning.

**Fig. 4:**
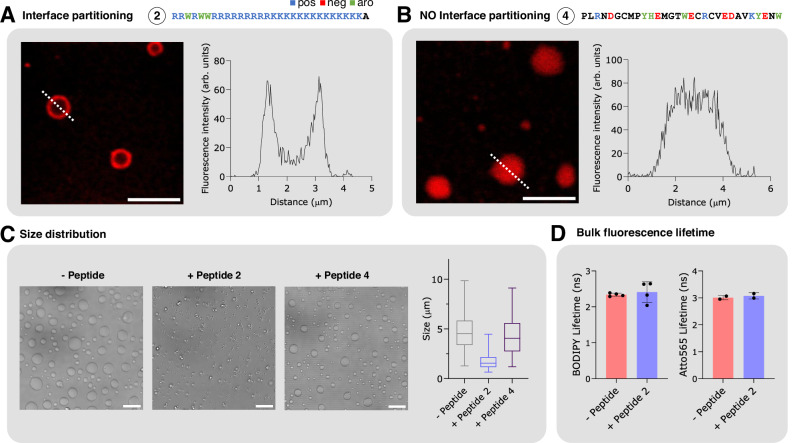
Experimental validation of interface partitioning at hnRNPA1-LCD condensates. Representative confocal microscopy images of the Cy5-labeled (**A**) selected peptide, with a high predicted propensity to localize at the interface, and (**B)** control peptide, which is not expected to localize at the interface. The fluorescent rim in (**A**) confirms the interfacial localization of the selected peptide as predicted by simulations. The amino acid sequences of both peptides are shown. Scale bar: 5 *μ*m. **C** Confocal microscopy images and corresponding size distributions (*n* = 50), which were also evaluated considering bright field microscopy images acquired using a different protein and peptide batch (Supplementary Fig. 10, *n* = 50). The addition of peptide 2 decreased the size of the condensate, while the addition of peptide 4 had a negligible impact. Box plots show median (line), 25th–75th percentiles (box), and minimum–maximum values (whiskers), based on pooled data (*n* = 100). Scale bar: 10 *μ*m. **D** Fluorescence lifetime analysis indicates that addition of peptide 2 does not significantly alter condensate bulk properties (mean ± SD, technical replicates).

We conjugated the synthesized peptides with the fluorescent dye Cy5, before incubation with hnRNPA1-LCD condensates at a protein-to-peptide molar ratio of 10:1. Analysis by fluorescence confocal microscopy confirmed that peptide 2 exhibits the strong predicted interface partitioning (Fig. 4A). Interfacial localization was consistent across different batches of protein and peptide, leading to full interfacial coverage of 91 % of the condensates (Supplementary Fig. 8). Formation of a fluorescent rim was also consistent over 5 h of incubation (Supplementary Fig. 9), and independent of the order in which protein and peptide were added, ruling out mass transfer limitations and confirming that the interfacial localization is thermodynamically driven. In contrast, the control peptide (peptide 4) with a much lower value for ${p}_{\,{{{\rm{int}}}}}^{*}$ distributed uniformly throughout the dense phase without forming a fluorescent rim (Fig. 4B). Interestingly, consistent with its interfacial localization, peptide 2 also caused a significant decrease in average condensate diameter (from 4.7 ± 1.8 *μ*m to 1.7 ± 0.8 *μ*m, mean ± SD, *n* = 100), as analyzed by confocal microscopy and bright field microscopy (Fig. 4C and Supplementary Fig. 10). Dynamic light scattering (DLS) analysis indicated that peptide 2 also promoted formation of clusters in the size range 500–1000 nm, below the limit of detection of microscopy (Supplementary Fig. 11), which would grow into larger micron-size condensates in the absence of the peptide. We further confirmed that these clusters did not form in the absence of the protein, as shown by fluorescence correlation spectroscopy (FCS) (Supplementary Fig. 12). This observation confirms that homotypic peptide interactions are minimized for this specific example, as no larger assemblies are produced by the peptide alone. The addition of control peptide 4 did not substantially affect the condensate diameter, which remained nearly unchanged at 4.2 ± 1.8 *μ*m (mean ± SD, *n* = 100). We further analyzed how the interface partitioning of peptide 2 alters the interface and the bulk of the hnRNPA1-LCD condensates. Supplementary Fig. 13 shows the number of contacts between condensates in the absence and presence of peptide 2 after 30 min of incubation, demonstrating a higher number of contacts for the sample with peptide 2 and, therefore, inhibited coalescence, consistent with the shift in size distribution. The properties in the bulk of the condensates were analyzed by quantifying the fluorescence lifetime of two small molecules (Atto-565-NHS ester, and BODIPY) using Fluorescence Lifetime Imaging Microscopy (FLIM). Fluorescence lifetime is sensitive to the dye’s local environment and, for BODIPY specifically, is associated with local viscosity^74^. As shown in Fig. 4D, the lifetime of both molecules in the interior of the condensates does not change upon peptide addition, suggesting a negligible effect of the peptide on the bulk.

### Designing peptides targeting other protein condensates

**Fig. 5:**
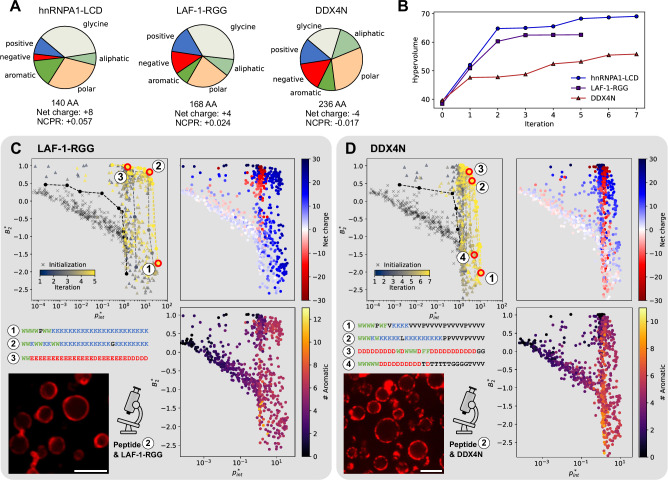
Expanding peptide design to other condensate targets: LAF-1-RGG and DDX4N. **A** Comparison of sequence composition among the three proteins of interest. hnRNPA1-LCD has the highest NCPR and the largest fraction of aromatics. In contrast, LAF-1-RGG and DDX4N both have fewer aromatic residues and lower net charges, with DDX4N showing the lowest NCPR. **B** Hypervolume progression for the peptide optimizations. **C** Peptide sequence optimization for LAF-1-RGG leads to surfactant-like peptides, mainly composed of tryptophans and lysines, where the overall composition remains nearly constant, but the sequence patterning varies along the Pareto front. Interfacial localization of a selected peptide could be confirmed using confocal microscopy. **D** Peptide sequence optimization for DDX4N mostly yielded sequences featuring valine-rich tails and polylysine patches, resulting in a net charge opposite to that of the condensate. Experiments also confirmed interfacial localization in vitro. Scale bar: 5 *μ*m.

We next applied our approach to other phase-separating proteins, to further understand and generalize the guiding principles behind interfacial localization for distinct condensate targets. We repeated the optimization procedure for condensates based on the LAF-1 RGG domain and the N-terminal disordered domain of DDX4. The sequence compositions of the three targeted proteins are clearly distinct, as illustrated in Fig. 5A, which shows the relative fraction of each amino acid category^45^. Notably, hnRNPA1-LCD has the highest net charge among the three disordered proteins, with a net charge per residue (NCPR) of +0.057, although it contains relatively few charged residues and a higher proportion of aromatic residues compared to the other two cases. In contrast, LAF-1-RGG and DDX4N have fewer aromatic residues and lower net charges, with NCPR values of +0.024 and −0.017, respectively. The progression of the optimization targeting LAF-1-RGG was comparable to that of hnRNPA1-LCD, as measured by hypervolume improvement (Fig. 5B). The majority of the gains occurred in the first two iterations, after which the hypervolume reached a plateau. In contrast, the optimization for DDX4N was more challenging, as the Pareto front progressed more slowly and the hypervolume stagnated at a lower level.

Similarly to the results obtained for hnRNPA1-LCD, the LAF-1-RGG peptide sequences exhibited a surfactant-like architecture (Fig. 5C), with one end enriched in tryptophans and the other in lysines. The lysine-rich region was excluded from the condensate phase in the simulations, as visible in the contact map (Supplementary Fig. 14). An important difference from the hnRNPA1-LCD case is that the peptides do not contain any arginine residues, potentially due to the low fraction of aromatic residues in LAF-1-RGG and the consequently lower relevance of cation–*π* interactions. Furthermore, the highest values for ${p}_{\,{{{\rm{int}}}}}^{*}$ are lower, and the polylysine tail is longer than for hnRNPA1-LCD, which could be a consequence of the lower net charge density of LAF-1-RGG condensates and thus a weaker driving force to exclude lysine residues. Another interesting observation is that the aromatic fraction and net charge remained almost constant along the main section of the Pareto front, covering a wide range of homotypic interaction strengths. The sequences mainly differed in patterning, as the aromatic patches become increasingly interrupted by self-repulsing lysines, which can be seen by, for instance, comparing peptides 1 and 2 in Fig. 5C. Splitting up patches of strongly interacting “sticker" residues and therefore decreasing their valency is known to decrease interaction strength^75^. Additionally, some sequences with high negative charge appeared in the Pareto front, exhibiting a slight preference for the interface over the dense phase (Supplementary Table 3). This could be due to a combination of favorable long-range electrostatic interactions but insufficient short-range attraction, leading to the observed behavior. As previously done with the hnRNPA1-LCD condensates, we assessed the spatial localization of a selected peptide in LAF-1-RGG condensates using fluorescence confocal microscopy. Consistent with the hnRNPA1-LCD case, we selected a Pareto-optimal peptide with a value of the primary ${p}_{int}^{*}$ objective close to the maximum observed, leading to an efficient tradeoff with low homotypic interactions. The peptide also localized at the interface, as indicated by the fluorescent rim (Fig. 5C). In contrast, a control peptide from the initialization did not partition at the interface and was instead homogeneously distributed within the condensate (Supplementary Fig. 15). The effect on the size distribution was similar to the hnRNPA1-LCD case, with the condensates being smaller in presence of the peptide partitioning at the interface (Supplementary Fig. 16). Also, coalescence was again inhibited (Supplementary Fig. 13), as observed by condensates more frequently being in contact without fusing, and addition of the peptide did not affect fluorescence lifetime of Atto565 and BODIPY in the bulk (Supplementary Fig. 17).

In the case of DDX4N (Fig. 5D), the optimization results differed significantly from the other two cases. Most Pareto-optimal sequences did not exhibit a tail matching the condensate’s net charge, as observed in the previous cases, even though such architectures were explored during the optimization process. One possible explanation for this behavior is the minimal net charge of DDX4N. Instead, the peptide sequences often featured valine-rich tails—which interact only weakly with most amino acids according to the Mpipi force field and are excluded from the dense phase (Supplementary Fig. 14)—as well as polylysine patches. While lysine exhibits favorable long-range electrostatic interactions with the condensate, its cation–*π* interactions are substantially weaker than those involving arginine, possibly aiding interfacial localization. This strategy appeared to perform better compared to variants matching the condensate’s net charge, such as peptide 4, which is not Pareto-optimal. Again, in agreement with in silico predictions, a peptide selected among the Pareto-optimal sequences did exhibit preferential interfacial partitioning in vitro (Fig. 5D), while a control peptide spread uniformly in the condensate (Supplementary Fig. 15). For DDX4N, and to some extent also for the other systems, interfacial localization is not perfectly uniform and results in multilayer adsorption or formation of a second phase wetting the condensates. This cannot be fully captured by our simulation pipeline, which optimizes adsorption in the low-concentration limit and only implicitly accounts for finite-concentration behavior by limiting homotypic peptide interactions. Nevertheless, the enrichment of peptides at the interface indicates that low-concentration design correlates with finite-concentration behavior and supports the applicability of our designs in this regime. As in the previous cases, interfacial partitioning also led to a decrease in DDX4N condensate size (Supplementary Fig. 16) and condensates in contact without fusing were more abundant in the presence of the peptide (Supplementary Fig. 13). In this case, however, the fluorescence lifetime of Atto565 and BODIPY appeared altered (Supplementary Fig. 17), suggesting that the peptide also affects bulk properties to some extent. While the peptide still shows a preference for the interface, its partial impact on bulk properties is consistent with the lowest hypervolume progress and the lowest predicted interface partitioning among the three optimization cases.

It is important to note that the optimality of the design depends on the accuracy of the Mpipi force field used. This may introduce potential bias during the optimization procedure, for example, due to the lack of secondary structure elements, explicit solvation, or the representation of the dominant role of *π*-based interactions in Mpipi. To evaluate this, we conducted an additional set of simulations using the CALVADOS 2^76^ and the more detailed Martini3-IDP^77^ force fields to examine the interface partitioning of the three experimentally validated peptides. This approach provided an orthogonal perspective on our designs. CALVADOS 2 simulations were performed at a slightly lower temperature (see “Methods” section), yielding comparable slab densities to those obtained with Mpipi. These simulations also indicated a preference for the peptides to localize in the interfacial region rather than in either the dense or dilute phases across all three cases. The interaction patterns observed in the contact maps were similar to those from Mpipi, exhibiting surfactant-like behavior (Supplementary Figs. 18 and 19).

In the case of Martini3-IDP, preference for the interfacial region over the condensate bulk was confirmed for the DDX4N and hnRNPA1-LCD systems, although in the latter case, the peptide mainly localized just beneath the condensate interface. The contact maps revealed still evident surfactant-like interaction profiles that share common features with the Mpipi results, although weaker and with some differences compared to Mpipi (Supplementary Figs. 20 and 21).

In conclusion, although as expected the optimal designs are likely to vary to some extent across different force fields, a certain degree of transferability exists. Continued advancements in force field development will further enhance the reliability of peptide designs generated by our computational pipeline.

## Discussion

In this study, we developed and applied a computational pipeline that integrates coarse-grained molecular simulations, machine learning, and MILP to de novo design peptides that partition at the interface of biomolecular condensates. The pipeline quantifies both the interfacial localization of isolated peptides and their homotypic interactions using coarse-grained simulations, followed by the training of a neural network and the identification of globally optimal sequences via bi-objective MILP. This approach led to sets of self-repulsive peptides with a high propensity to partition at the interface of three distinct condensates based on hnRNPA1-LCD, LAF-1-RGG, and DDX4N. Interfacial accumulation of the designed peptides was experimentally confirmed in vitro for all three cases, validating the design approach. Furthermore, at sub-stoichiometric concentrations, the peptides shifted the condensate size distribution toward smaller sizes, an effect that could be leveraged to tune condensate behavior^78^. This is consistent with findings showing that interface-localizing molecules can influence both the coalescence behavior and the surface tension of biomolecular condensates^28,34^.

**Fig. 6:**
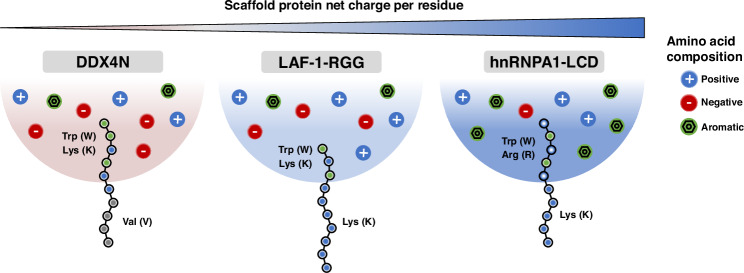
Schematic illustration of the design outcome for interface-partitioning peptides based on distinct condensate targets. The peptides exhibit surfactant-like architectures: one tail inserts into the condensate and is enriched in aromatic residues, while the opposite tail is excluded from the dense phase, with its sequence varying according to the scaffold’s net charge.

The peptide sequences generally exhibited surfactant-like architectures with two tails, one interacting with the scaffold molecule of the condensate and a second one excluded from the dense phase (Fig. 6). This architecture is similar to the amphiphilic, sometimes also referred to as condensate-amphiphilic, nature of larger proteins, polymers, and PEG-modified peptides previously shown to partition at the interface of biomolecular condensates^8,11–13,23–28,34–36^. In all cases, the tail of the peptide entering the dense phase was enriched in aromatic residues (and also in arginine for the hnRNPA1-LCD case), to promote attractive interactions with the scaffold molecule of the condensates. In contrast, the tail that was excluded from the condensates was different for the distinct IDRs. In the case of hnRNPA1-LCD and LAF-1-RGG, the tail primarily comprised lysine residues and matched the net charge of the condensate-forming protein, therefore promoting electrostatic repulsive interactions with the scaffold molecule of the condensates. This polylysine tail was longer for LAF-1-RGG, likely due to the lower positive net charge density of the protein. In the case of DDX4N, which has the lowest net charge density of the three protein targets, the mechanism behind the exclusion of the tail from the condensates cannot be based on electrostatic repulsion. Instead, interactions with the scaffold molecules were minimized by introducing uncharged valine residues in the excluded tail, which exhibit negligible interactions with the scaffold protein. These findings indicate that the net charge of the condensate-forming protein is a key physicochemical feature for designing peptides exhibiting specific interfacial localization.

While exhibiting surfactant-like architecture, our designed peptides also have key differences from classical surfactant molecules^79,80^. Instead of relying on alkyl tails to drive exclusion from the aqueous phase through hydrophobic interactions, our peptide designs primarily depend on cation–*π* and *π*–*π* interactions involving aromatic residues. Moreover, unlike classical surfactants, the optimization allocates a significant fraction, if not the majority, of the peptide molecule to residues that preferentially localize in the dilute aqueous phase, thereby preventing complete recruitment into the dense phase.

Overall, these findings and the developed pipeline provide a promising strategy for the rational design of interface-localizing peptides or protein sequences tailored to a specific condensate. It is important to note that the simulation setup used to quantify interfacial partitioning assesses the behavior of individual peptides. Therefore, the pipeline optimizes interfacial localization at low concentrations. A natural extension of this computational pipeline would involve conducting simulations at varying concentrations, accounting for lateral peptide interactions within the interfacial region. It should also be noted that the optimality of the design depends on the accuracy of the force field used, although we observed a reasonable agreement across different force fields for our selected peptides. Furthermore, the active learning framework using neural networks and MILP for sequence optimization can be applied to other design targets, avoiding getting trapped in local optima. In the future, the approach could also be expanded to more complex condensate compositions, also involving globular domains and client molecules, more closely mimicking the environment of cellular biomolecular condensates.

## Methods

### Molecular simulations

Generally, molecular simulations were carried out using the Mpipi force field^39^ in an NVT ensemble applying a Langevin thermostat^81^ at 300 K with a relaxation time of 100 ps. Simulations were performed using the LAMMPS Molecular Dynamics package (version 2nd of August 2023)^82^ and the ABF method was implemented using the Colvars module^83^.

#### Interface partitioning

The condensate dense phase was modeled using 16 protein copies within a periodic box, with dimensions 71 × 71 × 343 Å^3^ (hnRNPA1-LCD), 76 × 76 × 450 Å^3^ (LAF-1-RGG), and 88 × 88 × 510 Å^3^ (DDX4N), originating from a 2 × 2 × 4 starting configuration with density 0.5 g/cm^3^. The dense phase was loosely restrained in the *z* direction using harmonic walls (force constant: 5 kcal mol^−1^ Å^−^^2^) to a maximum width of 145 Å(hnRNPA1-LCD), 160 Å(LAF-1-RGG), and 170 Å(DDX4N), preventing strong fluctuations of the interface or dissociation of proteins into the dilute phase. The small system size and harmonic walls enable faster simulations with acceptable statistical error, for instance, allowing resolution of the effects of single amino-acid substitutions that remove or add aromatic residues, as tested in initial trial runs with the hnRNPA1-LCD system. The z-component of the center-of-mass distance between the peptide and dense phase (hnRNPA1-LCD: 0–12 nm, LAF-1-RGG/DDX4N: 0–12.8 nm) served as the collective variable for the ABF method. This range was divided into four sections, resulting in four separate simulations whose PMFs were later combined. Two PMFs were generated per simulation, with one peptide positioned on each side of the slab, ensuring the peptides remained in separate sections where interactions are avoided. This process was replicated three times, leading to a total of 6 PMFs obtained from 12 independent simulations for every peptide. The PMF was constructed using a total number of 64 bins, applying the biasing force after collecting 5000 samples per bin. Each simulation began with energy minimization and 5 ns of equilibration, allowing the system to relax, as assessed by the deviation from initial coordinates and the total potential energy (Supplementary Fig. 22), followed by a 250 ns production run. *ΔG*_1_ was determined as the mean difference between the PMF value at 0 nm (dense phase) and the global minimum, while *ΔG*_2_ was calculated as the free energy difference between the values at 0 nm (dense phase) and 12/12.8 nm (dilute phase). To validate that the small system size does not significantly affect our results, we repeated the simulation protocol for a larger system containing 128 protein copies (COM distance: 0–18 nm, harmonic walls: 250 Å, 10 replicas) for the experimentally validated hnRNPA1-LCD/peptide system, and observed highly similar results (Supplementary Fig. 23).

#### Homotypic interactions

Homotypic interactions were quantified by constructing the PMF as a function of the center-of-mass distance between two peptide copies, ranging from 0 to 70 Å, using the ABF method in a 140 × 140 × 140 Å^3^ simulation box with periodic boundary conditions. The bin width was set to 0.5 Å and the biasing force was applied after collecting 500 samples per bin. Energy minimization and 1 ns of equilibration were followed by 1 *μ*s production simulation, repeated eight times for each peptide. The PMF was converted into the second virial coefficient *B*_2_ according to:^37,51^

2 $${B}_{2}=2\pi {\int }_{0}^{{r}_{\max }}\left[1-\exp \left(-\frac{\,{{{\rm{PMF}}}}(r)}{{k}_{{{{\rm{B}}}}}T}\right)\right]\,{r}^{2}dr$$

A bi-symmetric log transformation^62^ was applied to the average *B*_2_ for every peptide, preventing a highly skewed distribution of data and the associated difficulties with training a machine learning model (*C* = 20 nm^3^).

3 $${B}_{2}^{*}=\,{{{\rm{sign}}}}\,({B}_{2}){\log }_{10}\left(1+\left|\frac{{B}_{2}}{C}\right|\right)$$

#### Contact maps and interfacial organization

The simulation setup for generating the contact maps was identical to the interface partitioning simulations, except without harmonic walls and ABF. Energy minimization and 30 ns of equilibration were followed by a 300 ns production simulation with coordinates saved every 50 ps. The contacts were analyzed using PLUMED^84^ (version 2.8.0) with a cutoff of 1.0 nm. For analyzing interfacial composition, simulations were repeated with different numbers of peptide copies using five independent replicas per condition, and the distribution of residues was analyzed using the MDAnalysis package^85,86^. At high peptide concentrations, occasional rupture of the protein slab occurred; the corresponding frames were identified using wavelet transformation-based detection of multiple density peaks^87^ and excluded from the analysis.

#### MARTINI simulations

Slab coexistence simulations were carried out in GROMACS 2021.4^88^ using the Martini3-IDP force field^77^, in the NPT ensemble applying a velocity-rescaling thermostat^89^ (300 K, 1 ps) and a Parrinello-Rahman barostat^90^ (1 bar, 12 ps). Initial peptide and IDP geometries and topology files were generated using Polyply^91^, followed by energy minimization and a 100 ps simulation, leading to compact conformations. The condensate slab was generated by random insertion of a total of 16 protein copies into the center of the periodic box (same box dimensions as in LAMMPS simulations), and two peptides were inserted into the dilute phase. Coarse-grained water (9595–25442 beads) and ions corresponding to an ionic strength of 150 mM were added, followed by energy minimization and short equilibration, yielding the initial configuration for the production runs. Each system was simulated in 16 independent replicas of 60 *μ*s; the final 20 *μ*s were used for analysis. Convergence was assessed based on the mean center-of-mass distance between the slab and the peptide, as well as the peptide concentration in the dense phase. Coordinates were saved every 10 ns, and the contact map was constructed identically to the LAMMPS simulations, using the positions of the backbone beads.

#### CALVADOS simulations

Slab coexistence simulations were carried out in OpenMM 8.2.0^92^ using the CALVADOS 2 force field at an ionic strength of 150 mM^76^, applying a Langevin thermostat at 260 K with a relaxation time of 100 ps. The simulation temperature was slightly lower than in the Mpipi runs, resulting in similar slab densities. Initial topology and coordinate files were generated using the CALVADOS Python package^93^. Sixteen proteins and two peptides were inserted into a simulation box with the same dimensions as in the LAMMPS simulations. After 100 ns of equilibration, a 300 ns production run was performed, and contact maps were constructed analogously to the other cases.

### Machine learning and optimization

After each round of simulations, a multi-output neural network with two fully connected hidden layers of 50 neurons each was trained on Δ*G*_1_, *ΔG*_2_, and ${B}_{2}^{*}$ with PyTorch^94^ (version 2.3.1) and scikit-learn^95^ (version 1.5.1) using a set of engineered sequence features (Supplementary Methods). All features and objectives were normalized to the range [−1, 1], and the Smooth L1 loss function was minimized with the Adam optimizer^96^. A multi-step learning rate scheduler was employed, decaying the learning rate by 0.9 at epochs 200, 400, 600, and 800. A batch size of 32 was used for training. The 10% of the training data was set aside as a validation set, and the model exhibiting the lowest validation loss was selected. In every iteration, an 80/20 random train-test split was used to optimize hyperparameters (initial learning rate and weight decay). The performance of the best model was then evaluated on a separate random holdout set (20%). The final model used for optimization was trained on the entire available dataset, after which pruning was applied: all weights and biases of the neural network with absolute values below 0.001 were set to zero, mitigating potential numerical issues when solving the MILP. A MILP formulation of the trained neural network was incorporated into the algebraic modeling framework Pyomo^66,67^ (version 6.7.3) using OMLT^55^ (version 1.1). In the first step, the two objectives, $\ln({p}_{int}^{*})$ and ${B}_{2}^{*}$, were separately optimized with Gurobi^68^, using the one-hot encoded amino acid sequence as the input. Details on integrating the AGGRESCAN^58^ predictor as a constraint and on converting *ΔG*_1_ and *ΔG*_2_ into $\ln({p}_{int}^{*})$ are provided in the Supplementary Methods. After determining the bounds, the *ε*-constrained method was applied by constraining ${B}_{2}^{*}$, resulting in 50 Pareto-optimal peptide sequences (exploitation). 25 well-spaced points were selected for simulation in the next round. For the exploration part, the weighted-sum method described in the main text was used to generate 150 sequences, from which a well-spaced subset of 75 sequences was selected. The weighted-sum method defines a combined objective using random weights assigned to the individual objectives, which differ for each exploration point. Furthermore, between 2 and 20 positions (chosen uniformly at random) were constrained to randomly selected amino acids. The remaining positions were then optimized. This approach strikes a balance between exploration and sampling relevant regions of the design space. In the first optimization with hnRNPA1-LCD as the condensate target, we reduced the number of exploration points to 25 for iterations 3 and 4, and to zero for the last three iterations, fully focusing on the exploitation component to limit computational cost.

### Protein and peptide production

Genes encoding the different LCDs were codon optimized for expression in *E*. *coli*, synthesized, and cloned between NdeI and BamHI restriction sites into the pET-15b vector by Genewiz (NJ, US). Recombinant proteins were produced in *E.* *coli* BL21(DE3) GOLD cells. Bacterial cultures were induced at OD 0.7 with 0.5 mM isopropyl D-thiogalactopyranoside and grown for an additional 16 h at 20 °C (DDX4N) or 37 °C (LAF-1-RGG and hnRNPA1-LCD). Cells expressing the proteins were centrifuged, re-suspended in lysis buffer (LAF-1-RGG/hnRNPA1-LCD: 8 M urea, 1 M NaCl, 10 mM imidazole, 50 mM Tris at pH 7.5, 2 mM *β*-mercaptoethanol; DDX4N: 1 M NaCl, 10 mM imidazole, 50 mM Tris at pH 8.5, 2 mM *β*-mercaptoethanol), lysed by sonication, and centrifuged at 18,300×*g* for 20 min. The supernatant was further used for purification of the proteins using immobilized-metal affinity chromatography (elution buffers: 1 M NaCl, 500 mM imidazole, 2 M urea; 50 mM Tris at pH 7.5, 2 mM *β*-mercaptoethanol for LAF-1-RGG/hnRNPA1-LCD and 1 M NaCl, 500 mM imidazole, 50 mM Tris at pH 8.5, 2 mM *β*-mercaptoethanol for DDX4N). Size exclusion chromatography using a Superdex 75 16/600 column (GE Healthcare) in 50 mM Tris at pH 7.5, 500 mM NaCl, 2 M urea, 10% glycerol for hnRNPA1-LCD; 50 mM Tris at pH 7.5, 500 mM NaCl, 2 M urea for LAF-1-RGG, and 50 mM Tris at pH 8.5, 1 M NaCl, 10% glycerol for DDX4N was used as the final purification step. The quality of the purified proteins was checked by SDS-PAGE electrophoresis. Peptides were purchased from GenScript with a purity of ≥95%. The Cy5 dye was conjugated to the N-terminus, while the C-terminus was amidated.

### Brightfield and confocal microscopy

The different LCDs (10 *μ*M) were incubated with either the corresponding interface peptide or a control peptide at a protein-to-peptide ratio of 10:1. For hnRNPA1-LCD, the buffer composition was 20 mM Tris, 150 mM NaCl, pH 7.5, whereas for LAF-1-RGG and DDX4N, the buffer consisted of 20 mM Tris, 25 mM NaCl, pH 7.5. Interface partitioning was assessed by measuring the intrinsic fluorescence of Cy5, excited with a 633 nm laser, with emission recorded at 670–700 nm, using a confocal microscope (Leica TCS SP8) equipped with a 63× NA 1.4 oil objective (Leica). Imaging was performed with the Leica Application Suite X (LAS X) software, version 1.0. The fluorescence intensity profiles across the condensates were extracted using ImageJ (version 1.53t)^97^.

Brightfield microscopy was used to estimate the size of protein condensates in the absence and presence of the different interface peptides. The different LCDs were incubated at 10 *μ*M, with and without the corresponding interface peptides, at a protein-to-peptide ratio of 10:1 in the corresponding buffer. After sedimentation, the condensates were visualized using a brightfield microscope (Eclipse Ti-E, Nikon) using a 60× oil objective (FI Plan Apo Lambda NA 1.4, Nikon). Their size distribution was analyzed using ImageJ. All samples were analyzed in 384-well plates (MatriPlate 384-Well Plate, Glass Bottom, Brooks).

### DLS

The size distribution of hnRNPA1-LCD condensates in the dilute phase, both in the absence and presence of peptide, was estimated using a Zetasizer Nano-ZS (Malvern, software version 7.13) at 25 °C. hnRNPA1-LCD at 10 *μ*M was incubated with and without the interface peptide at a protein-to-peptide ratio of 10:1 in 20 mM Tris, 150 mM NaCl, pH 7.5. After 1 h of incubation, the samples were centrifuged at 8,000×*g* for 30 min to separate the dilute and dense phases.

### FCS

FCS experiments were performed using an inverted confocal fluorescence microscope (Leica SP8 STED) equipped with an HC PL APO CS2 63 × 1.2 NA water immersion objective and a hybrid detector for single molecule detection (HyD SMD). Data acquisition and analysis were performed using LAS X software (version 1.0). For the analysis of the self-association behavior of peptide 2 targeting the interface of hnRNPA1-LCD condensates, 500 nM Cy5-labeled peptide was incubated with 500 nM unlabeled peptide, reaching a final peptide concentration of 1 *μ*M in 20 mM Tris, 150 mM NaCl, pH 7.5 (*n* = 3). Samples were excited with a 640 nm laser (from a White Light Laser at 80 MHz repetition frequency), and the fluorescence emission was collected at a wavelength range of 660–700 nm. The confocal volume was calibrated using 20 nM Alexa 647 NHS Ester (Diffusion coefficient *D* = 330 *μ*m^2^/s), and the autocorrelation curves were fitted based on a model assuming a single diffusing component.

### FLIM

FLIM experiments were performed using an inverted confocal fluorescence microscope (Leica SP8 STED, LAS X software, version 1.0) equipped with a HC PL APO CS2 63 × 1.2 NA water immersion objective and a hybrid detector for single molecule detection (HyD SMD).

The different LCDs were incubated at 10 *μ*M in the absence or presence of 1 *μ*M interface peptide, together with 1 nM Atto-565 NHS Ester or 1 *μ*M BODIPY in the corresponding buffer. The lifetime of Atto-565 NHS Ester inside condensates was measured by exciting the sample at 555 nm and collecting the fluorescence emission at 575–610 nm. For BODIPY, samples were excited at 506 nm, and the emission was recorded at 520–550 nm. Images were fitted pixel by pixel with a two-component exponential reconvolution model, and the calculated mean lifetime intensity weighted was used for comparison between the different samples. At least 25 condensates were analyzed per image.

### Reporting summary

Further information on research design is available in the Nature Portfolio Reporting Summary linked to this article.

## Supplementary information

Supplementary Information

Reporting Summary

Transparent Peer Review file

## Source data

Source Data

## Data Availability

Unless otherwise stated, all data supporting the results of this study can be found in the article, supplementary, and source data files. Source data are provided with this paper.

## Code availability

The developed code, molecular dynamics input files, and final configurations are available on Zenodo at [10.5281/zenodo.19387479].
