# Supplementary material for: De novo design of peptides localizing at the interface of biomolecular condensates
Source: Nat Commun. 2026 May 16;17:6497. doi: 10.1038/s41467-026-73099-9 (PMC13376736; doi:10.1038/s41467-026-73099-9)
Supplement: Supplementary file 1 — Supplementary Information [file 41467_2026_73099_MOESM1_ESM.pdf]

Supplementary Figures

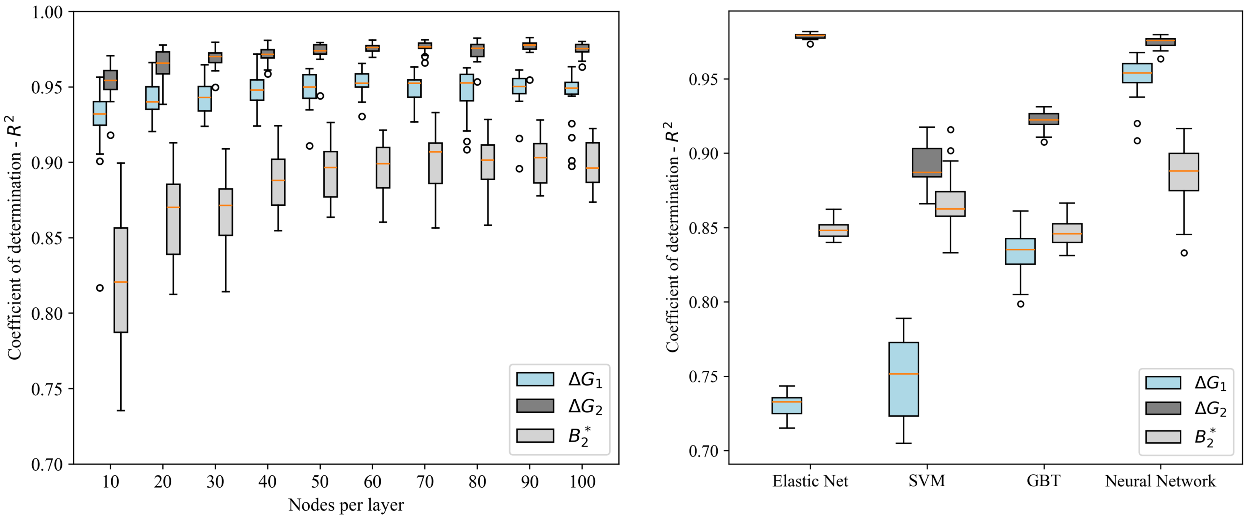

Supplementary Figure 1: Benchmarking the performance of a two-layer neural network with varying layer widths against alternative models (elastic net, support vector machines, and gradient-boosted trees), using the initialization data for the hnRNPA1-LCD condensate target. Based on these results, a neural network width of 50 was chosen. Box plots: median (line), 25th–75th percentiles (box; IQR $= Q_3 - Q_1$), whiskers to $\pm 1.5 \times$ IQR, n=20.

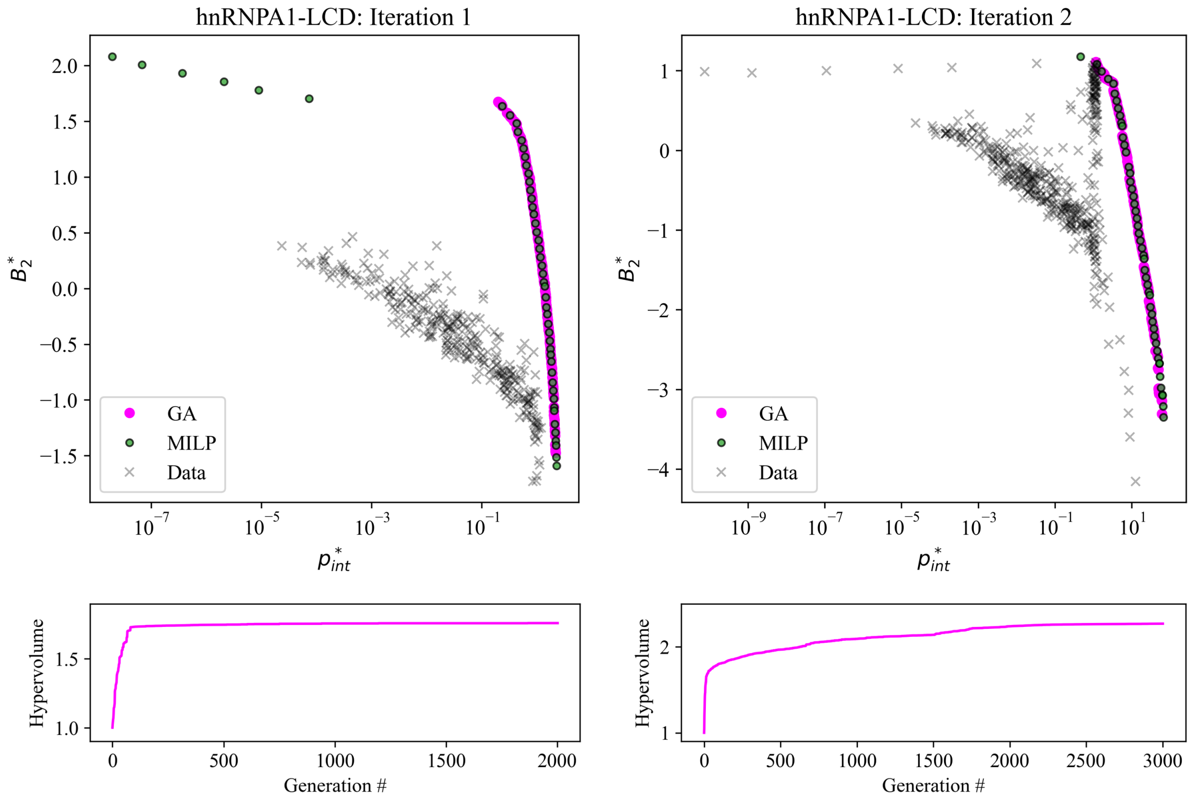

Supplementary Figure 2: Comparison of the genetic algorithm (GA) and mixed-integer linear programming (MILP) in constructing Pareto fronts of the first two trained surrogate models in the hnRNPA1 optimization case. While the GA captured most of the true Pareto front identified by MILP for both models, it failed to recover a significant portion in the iteration 1 model, despite hypervolume convergence.

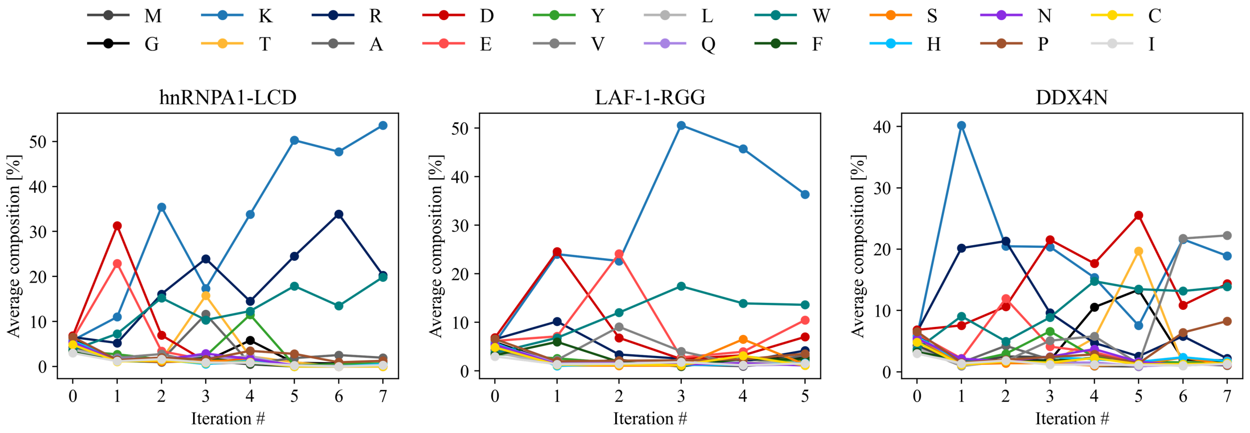

Supplementary Figure 3: Average peptide composition across iterations for all optimization runs.

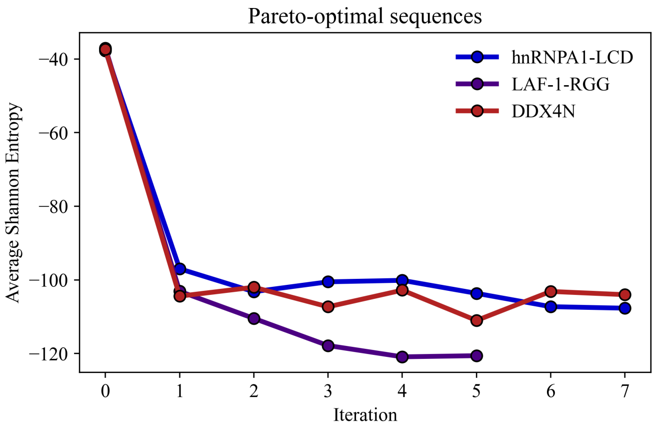

Supplementary Figure 4: Average Shannon entropy[1] of Pareto-optimal sequences across iterations for all optimization cases. The decreasing entropy indicates that the final optimal sequences were composed of only a subset of amino acids.

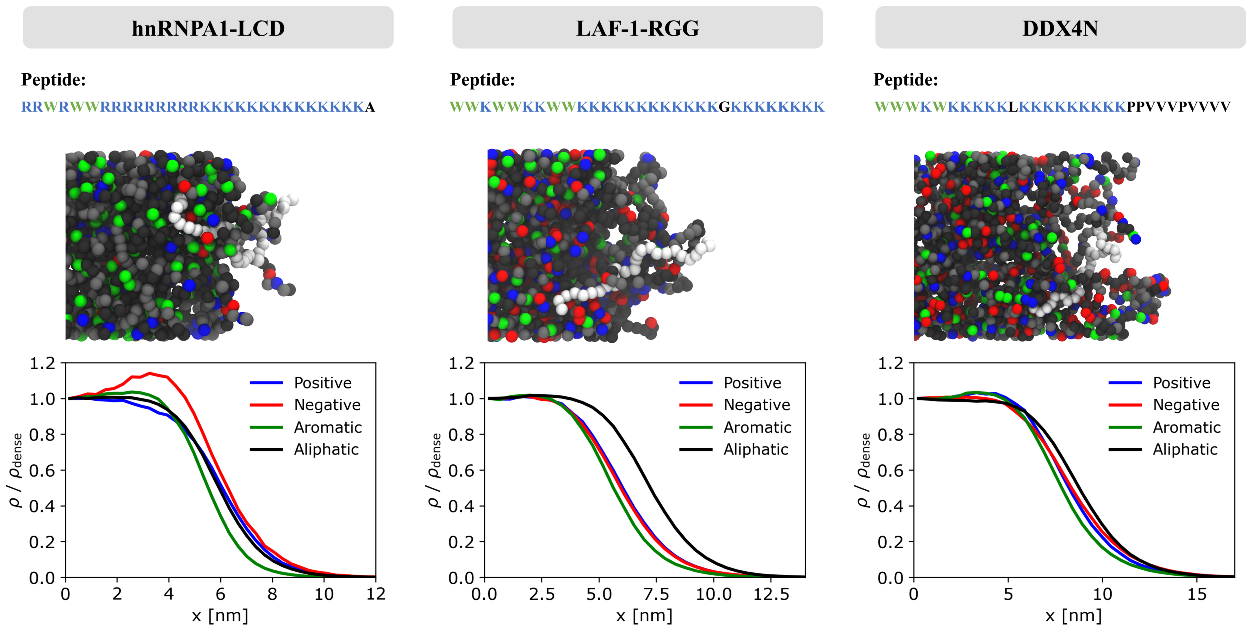

Supplementary Figure 5: Distribution of different protein residue types (positive: K/R, negative: D/E, aromatic: Y/W/F, aliphatic: G, A, V, I, L, M, P) within the dense phase of the simulation box containing 16 protein copies and 2 peptides. x is the distance from the slab center of mass.

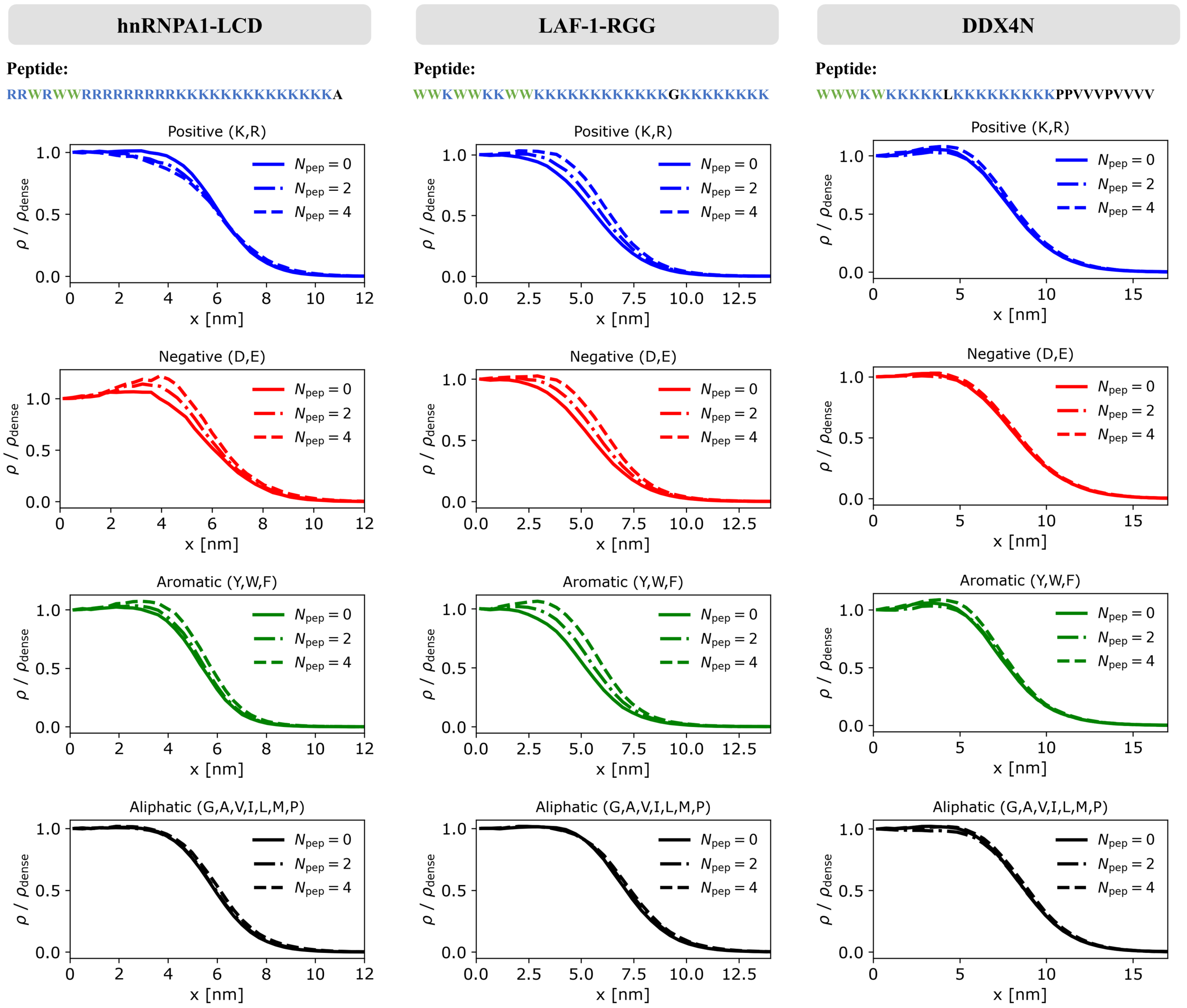

Supplementary Figure 6: Distribution of different protein residue types within the dense phase as a function of the distance from the slab center of mass for varying numbers of peptides in the simulation box.

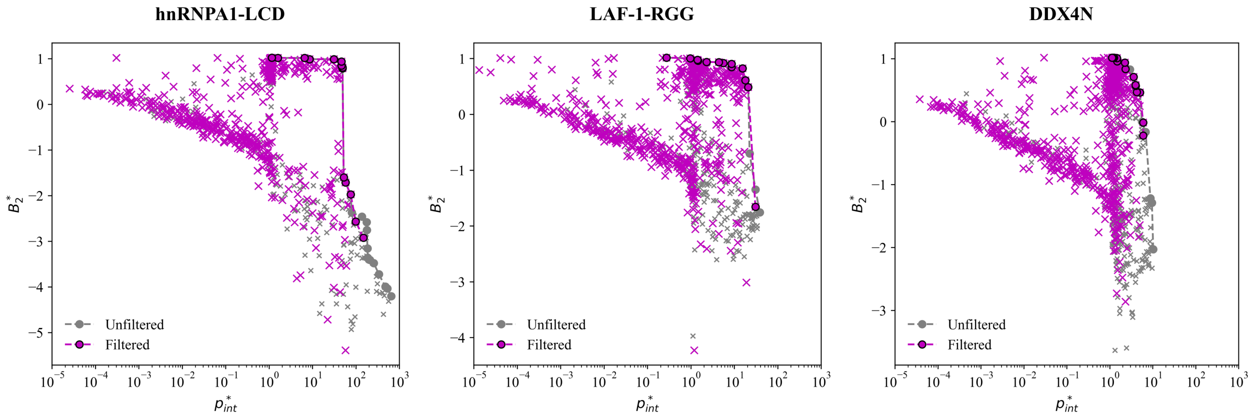

Supplementary Figure 7: Filtered optimization results for all design cases. The final filtering was performed by applying the TANGO[2] and Waltz[3] (threshold=85) aggregation predictors, as described in the main text.

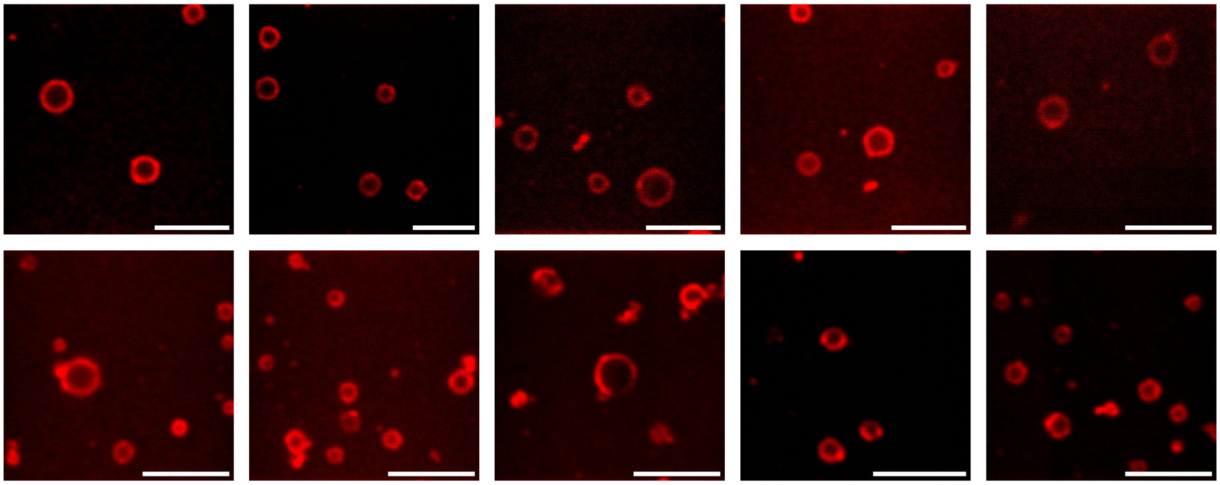

Supplementary Figure 8: Representative confocal microscopy images showing the interfacial localization of peptide 2 in hnRNPA1-LCD condensates, at 10 μM protein and a protein-to-peptide molar ratio of 10:1. Different samples were prepared from two independent protein and peptide batches. The fraction of condensates (diameter > 1 μm) exhibiting complete interfacial coverage is 91 % (n = 34). Scale bar: 5 μm.

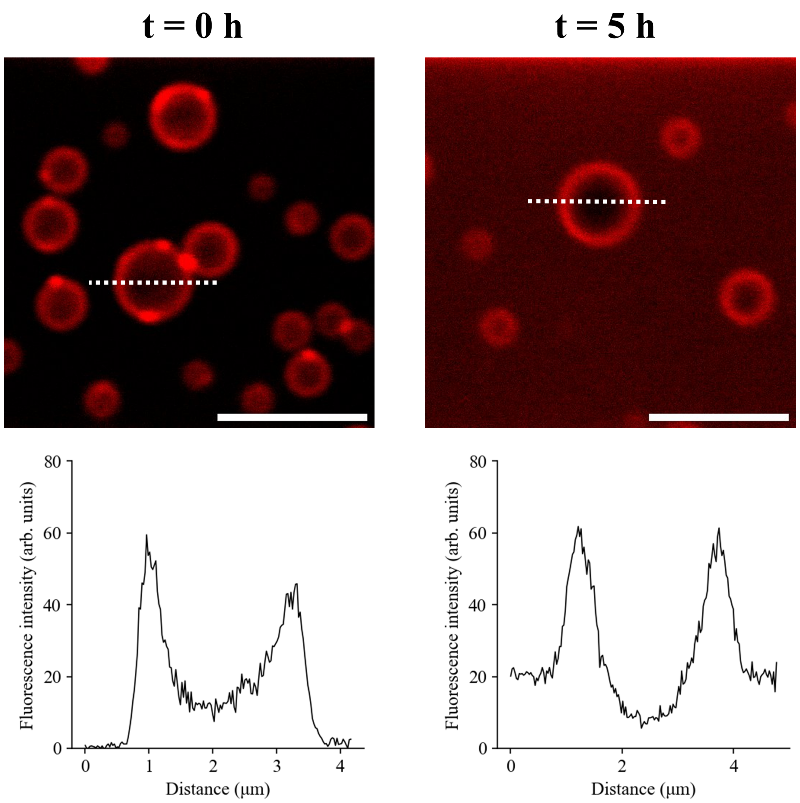

Supplementary Figure 9: Interfacial localization of designed peptide at the condensate formed by hnRNPA1-LCD (10 μM protein/peptide, 20 mM Tris (pH 7.5) and 150 mM NaCl) is consistent over 5 hours of incubation. Scale bar: 5 μm.

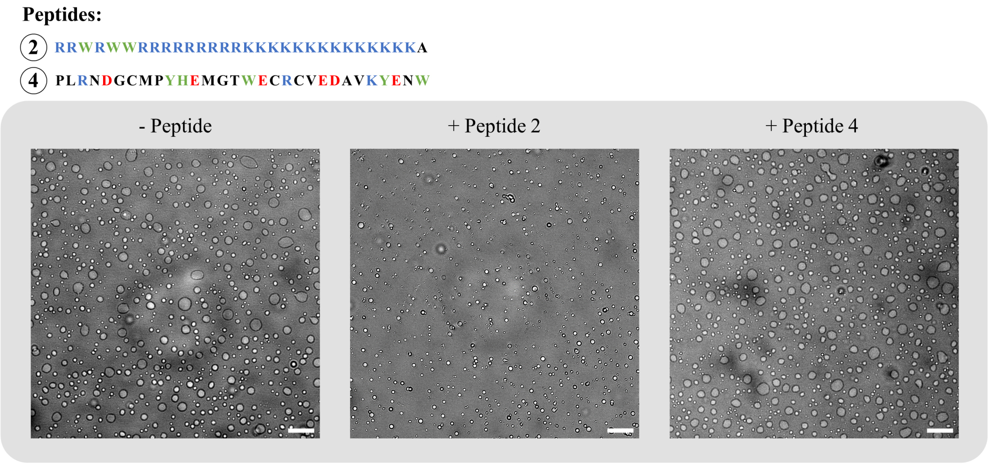

Supplementary Figure 10: Bright field microscopy images of hnRNPA1-LCD condensates in the absence and presence of peptide 2 or peptide 4. Scale bar: 20 μm.

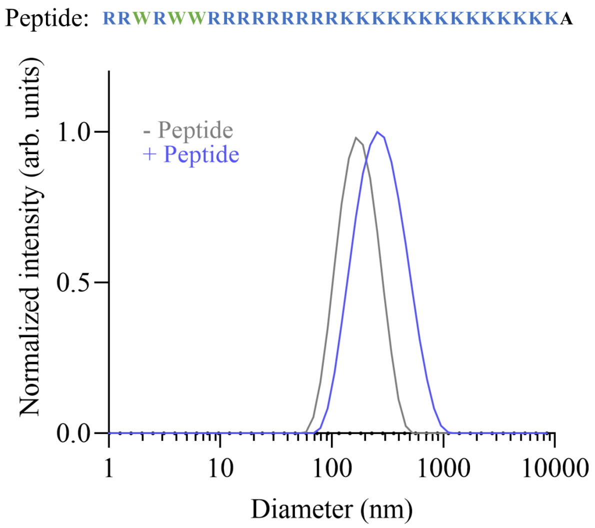

Supplementary Figure 11: Dynamic light scattering (DLS) analysis of the supernatant size distribution of hnRNPA1-LCD condensates, with and without peptide 2.

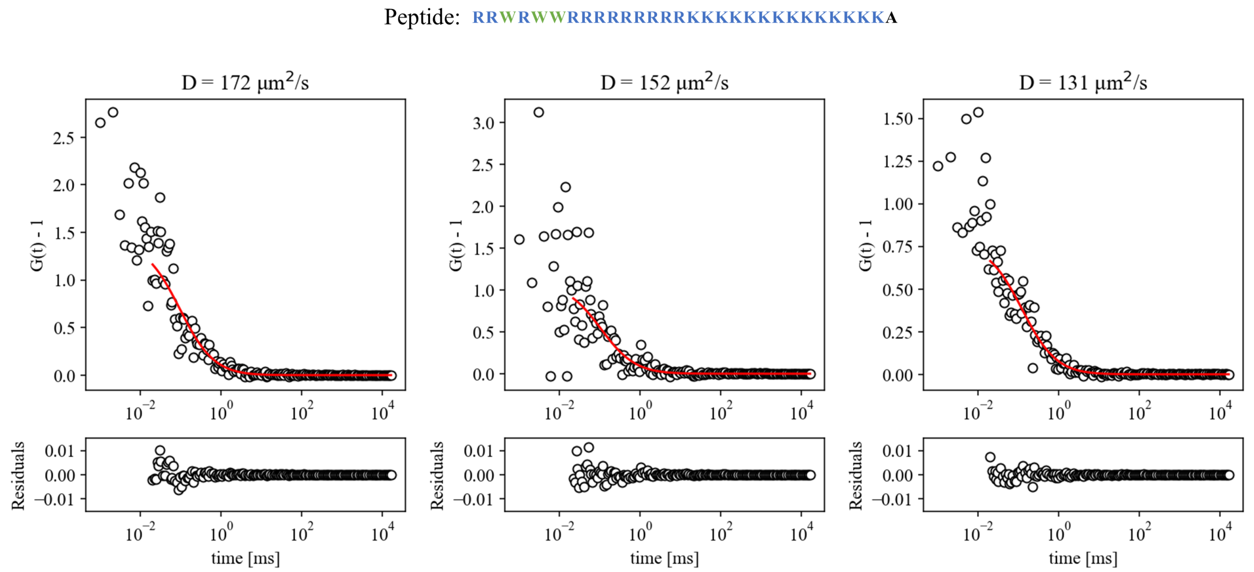

Supplementary Figure 12: FCS results (3 replicas) of the peptide 2 alone (1 μM, 50% labeled) in 20 mM Tris (pH 7.5) and 150 mM NaCl. The fitted diffusion coefficient was 152 $\pm$ 20 $\mu m^2/s$, corresponding to a hydrodynamic radius of 1.41 $\pm$ 0.19 nm calculated using the Stokes–Einstein equation (mean $\pm$ SD, $n = 3$, technical replicates). The theoretical radius of gyration of a monomer, assuming a C-α separation distance of 0.381 nm, is estimated to be between 0.9 nm (ideal chain: $R_g = \sqrt{N}b/\sqrt{6}$) and 3.3 nm (rod: $R_g = Nb/\sqrt{12}$).[4]

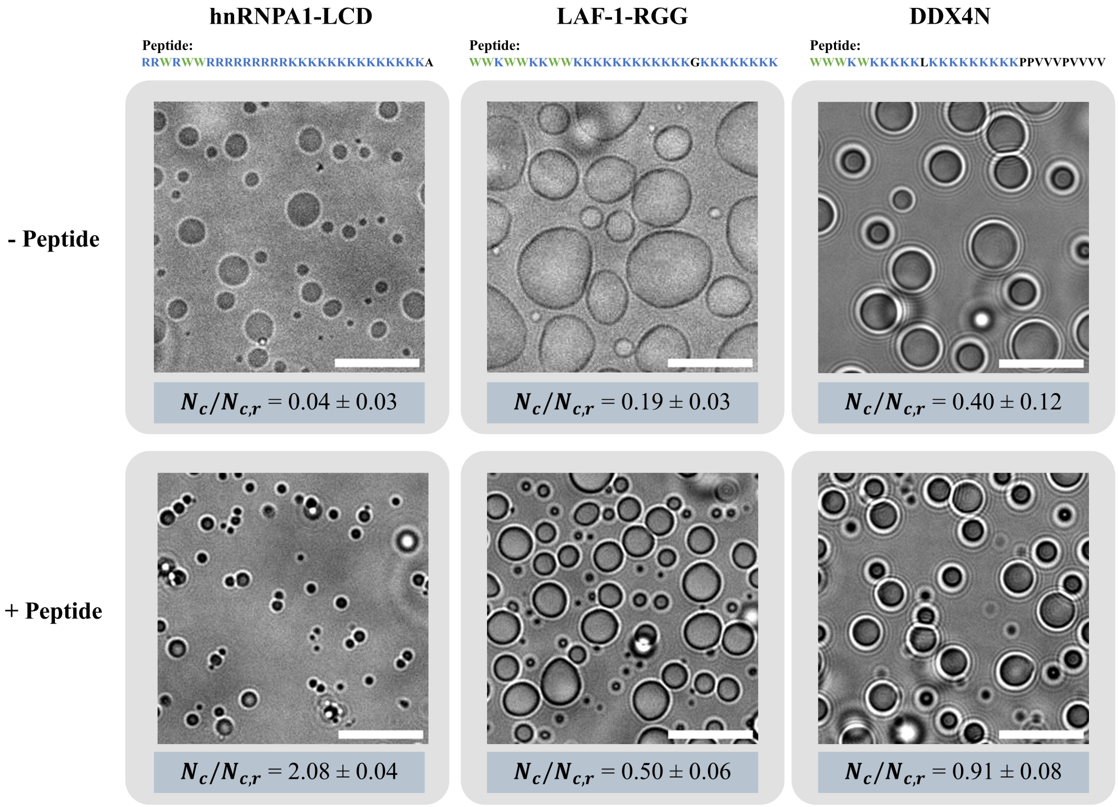

Supplementary Figure 13: Analysis of the number of contacts N_c between condensates formed under different conditions after 30 minutes of incubation. To account for differences in size distribution, we normalized by the number of contacts expected from randomly redistributing the condensates within the observation window, $N_{c,r}$. Assuming circular geometry, independent placement, and neglecting border effects, the overlap probability between two condensates is given by $p_c(A, B) = \frac{(r_A+r_B)^2\pi}{A_{\text{tot}}}$, leading to an expected number of contacts $N_{c,r} = \frac{\pi}{A_{\text{tot}}} \sum_{i>j}(r_i + r_j)^2$. Results show that contacts between condensates with the interface-localizing peptides present are generally more likely, demonstrating inhibited coalescence and therefore altered surface properties. Three windows of 128×128 μm^2 were analyzed for each condition (mean $\pm$ SD, n=3). Scale bar: 20 μm.

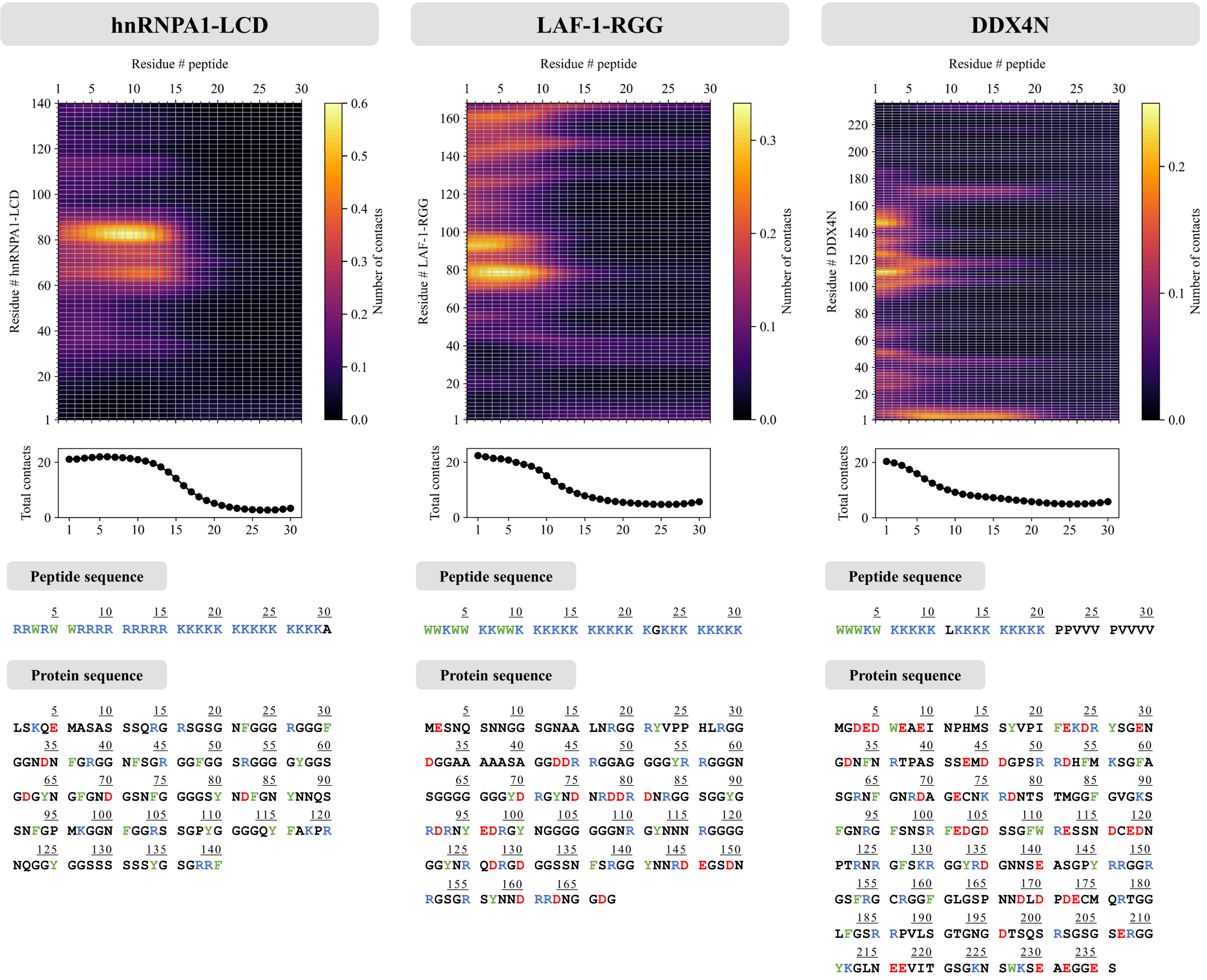

Supplementary Figure 14: Protein–peptide contact maps for the experimentally validated cases. The hnRNPA1-LCD case is also shown in Figure 3.

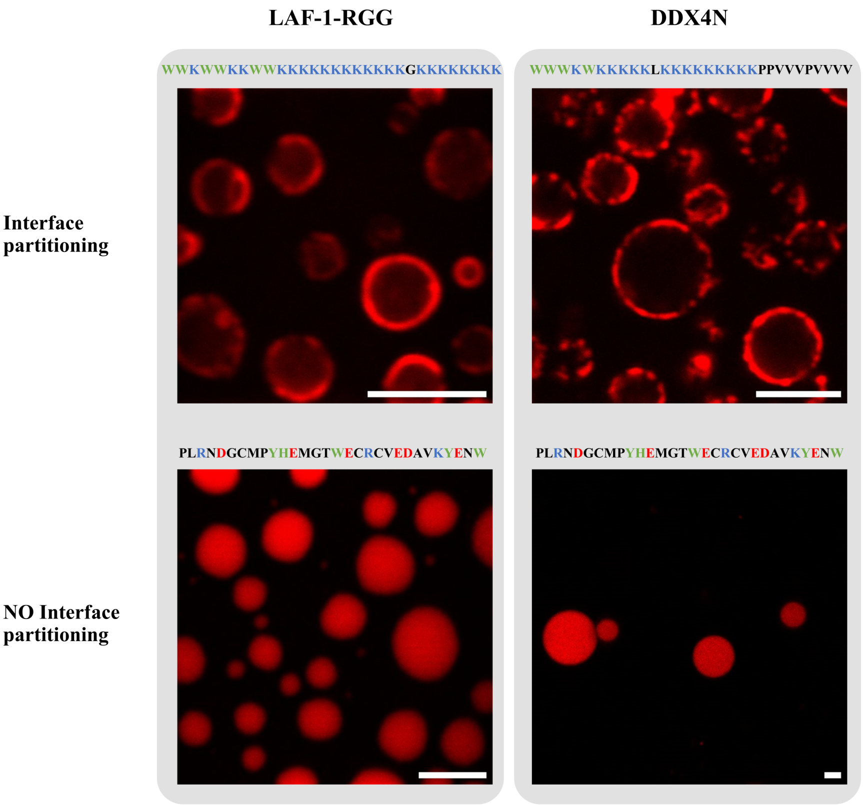

Supplementary Figure 15: Experimental validation of interface partitioning at condensates formed by the proteins LAF-1-RGG and DDX4N. Fluorescence confocal microscopy confirmed that peptides with high predicted preference for the interface accumulated at the condensate interface (top), while the control peptide spread uniformly (bottom). Scale bar: 5 μm.

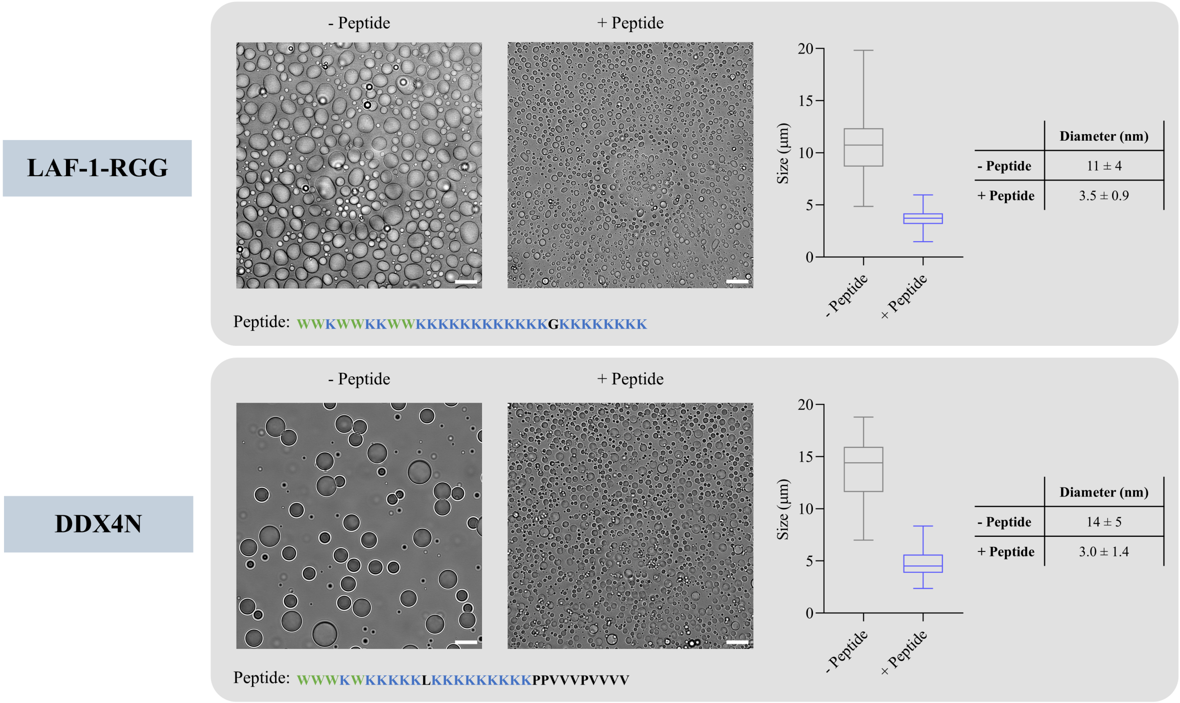

Supplementary Figure 16: Impact of interface-partitioning peptides on condensate size distribution. In both cases, a decrease in average droplet diameter is observed. The diameters reported in the tables correspond to the mean ± standard deviation (n = 50). Box plots show median (line), 25th–75th percentiles (box), and minimum–maximum values (whiskers). Scale bar: 20 μm.

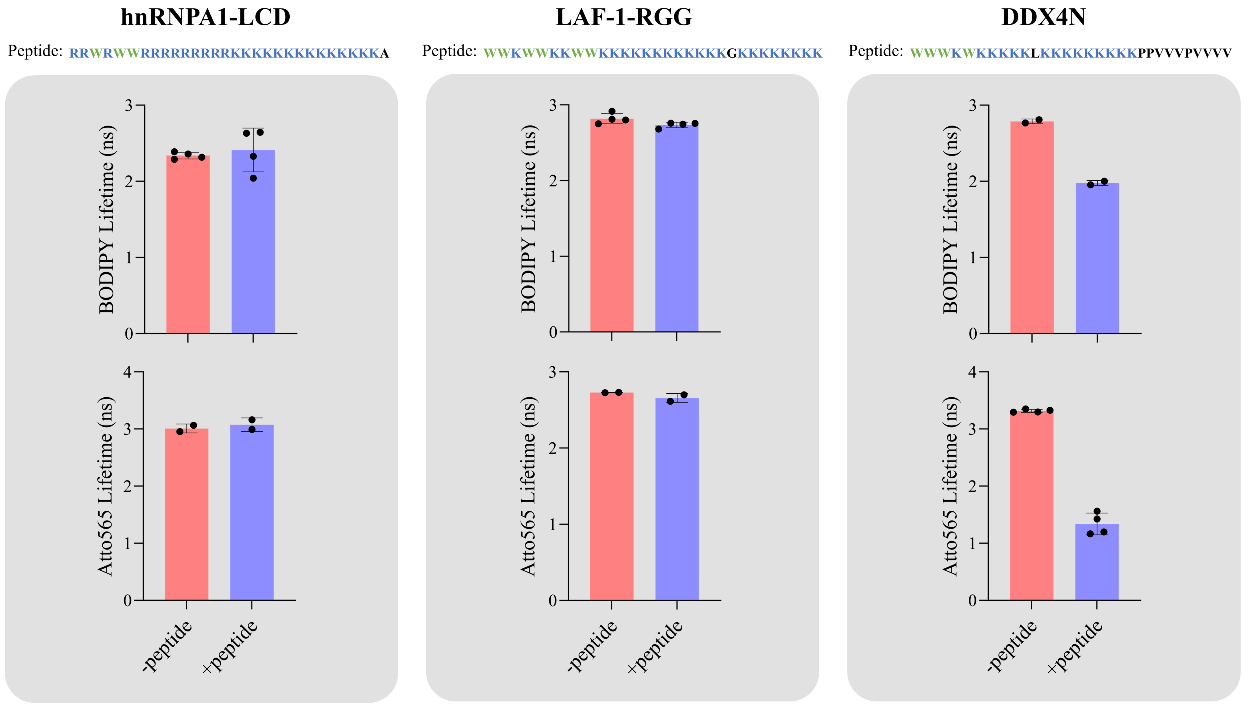

Supplementary Figure 17: Impact of interface-partitioning peptides on the fluorescence lifetime of two different dyes in the condensate bulk (mean ± SD, technical replicates). In two out of three cases, the lifetime is unaffected.

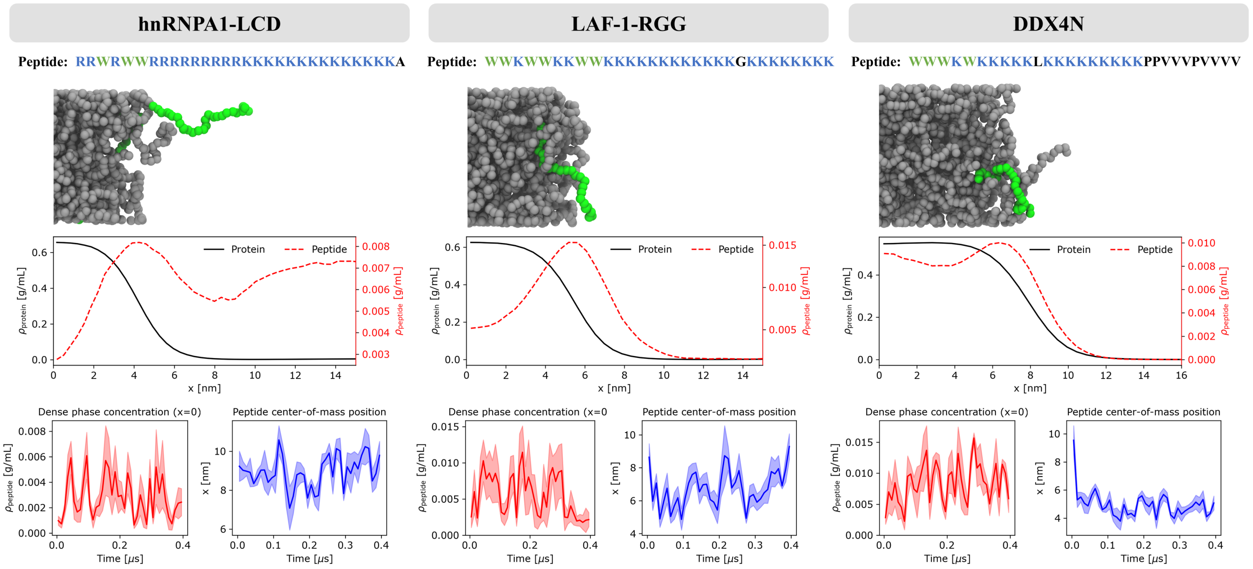

Supplementary Figure 18: Results of CALVADOS 2^5 slab coexistence simulations. Convergence was assessed from the peptide dense-phase concentration and center-of-mass position. The final 300 ns were used to construct density profiles and contact maps. Uncertainties correspond to standard errors from independent simulations (n=5).

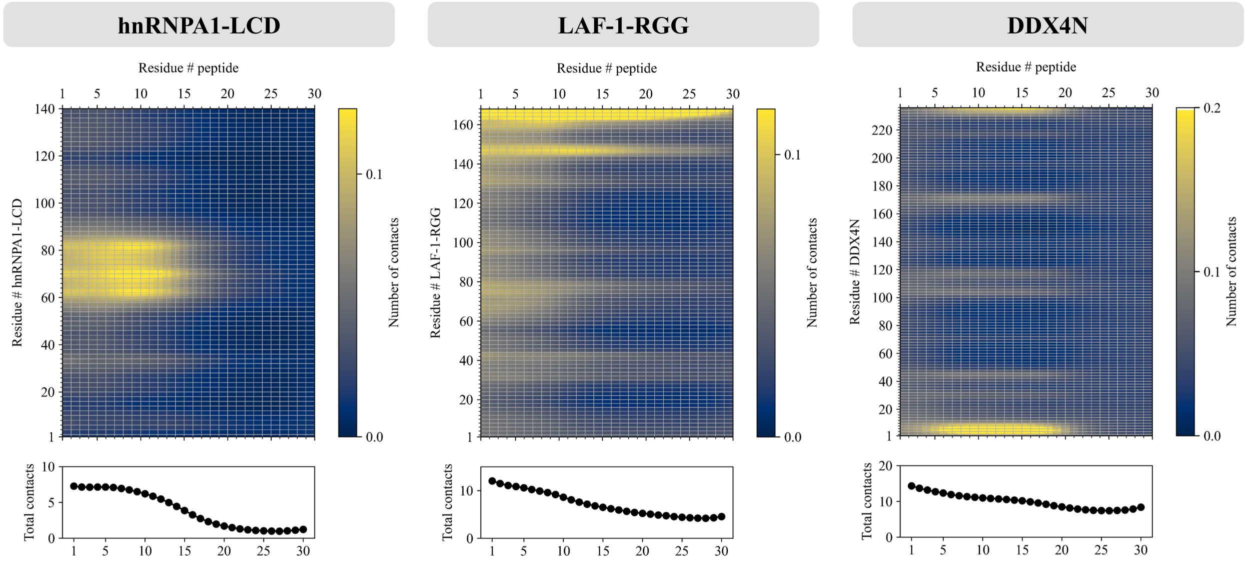

hnRNPA1-LCD

Peptide sequence

RRWRW WRRRR RRRRR KKKKK KKKKK KKKKA

Protein sequence

LSKQE MASAS SSQRG RSGSG NFGGG RGGGF
GGNDN FGRGG NFSGR GGFGG SRGGG GYGGS
GDGYN GFGND GSNFG GGGSY NDFGN YNNQS
SNFGP MKGGN FGGRS SGPYG GGGQY FAKPR
NQGGY GGSSS SSSYG SGRRF

LAF-1-RGG

Peptide sequence

WWKWW KKWWK KKKKK KKKKK KGKKK KKKKK

Protein sequence

MESNQ SNNGG SGNAA LNRGG RYVPP HLRGG
DGGAA AAASA GGDDR RGGAG GGGYR RGGGN
SGGGG GGGYD RGYND NRDDR DNRGG SGGYG
RDRNY EDRGY NGGGG GGGNR GYNNN RGGGG
GGYNR QDRGD GGSSN FSRGG YNNRD EGSDN
RGSGR SYNND RRDNG GDG

DDX4N

Peptide sequence

WWWKW KKKKK LKKKK KKKKK PPVVV PVVVV

Protein sequence

MGDED WEAEI NPHMS SYVPI FEKDR YSGEN
GDNFN RTPAS SSEMD DGPSR RDHFM KSGFA
SGRNF GNRDA GECNK RDNTS TMGGF GVGKS
FGNRG FSNSR FEDGD SSGFW RESSN DCEDN
PTRNR GFSKR GGYRD GNNSE ASGPY RRGGR
GSFRG CRGGF GLGSP NNDLD PDECM QRTGG
LFGSR RPVLS GTGNG DTSQS RSGSG SERGG
YKGLN EEVIT GSGKN SWKSE AEGGE S

Supplementary Figure 19: Protein–peptide contact maps for the experimentally validated cases, generated using the CALVADOS 2[5] force field.

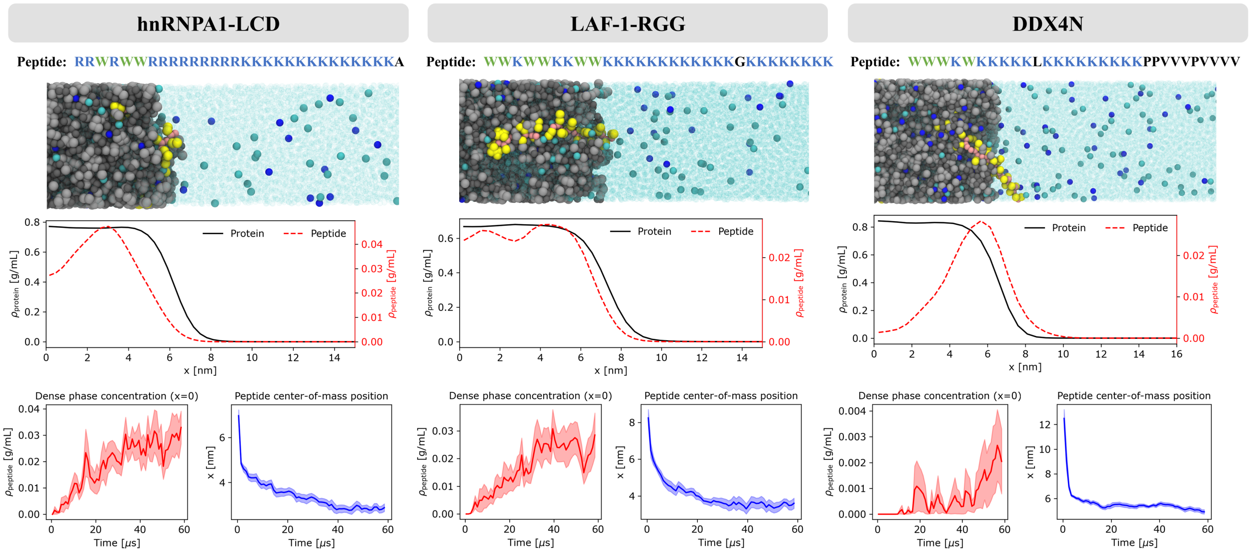

Supplementary Figure 20: Results of Martini3-IDP[6] slab coexistence simulations. Convergence was assessed from the peptide dense-phase concentration and center-of-mass position. The final 20 μs were used to construct density profiles and contact maps. Uncertainties correspond to standard errors from independent simulations (n=16).

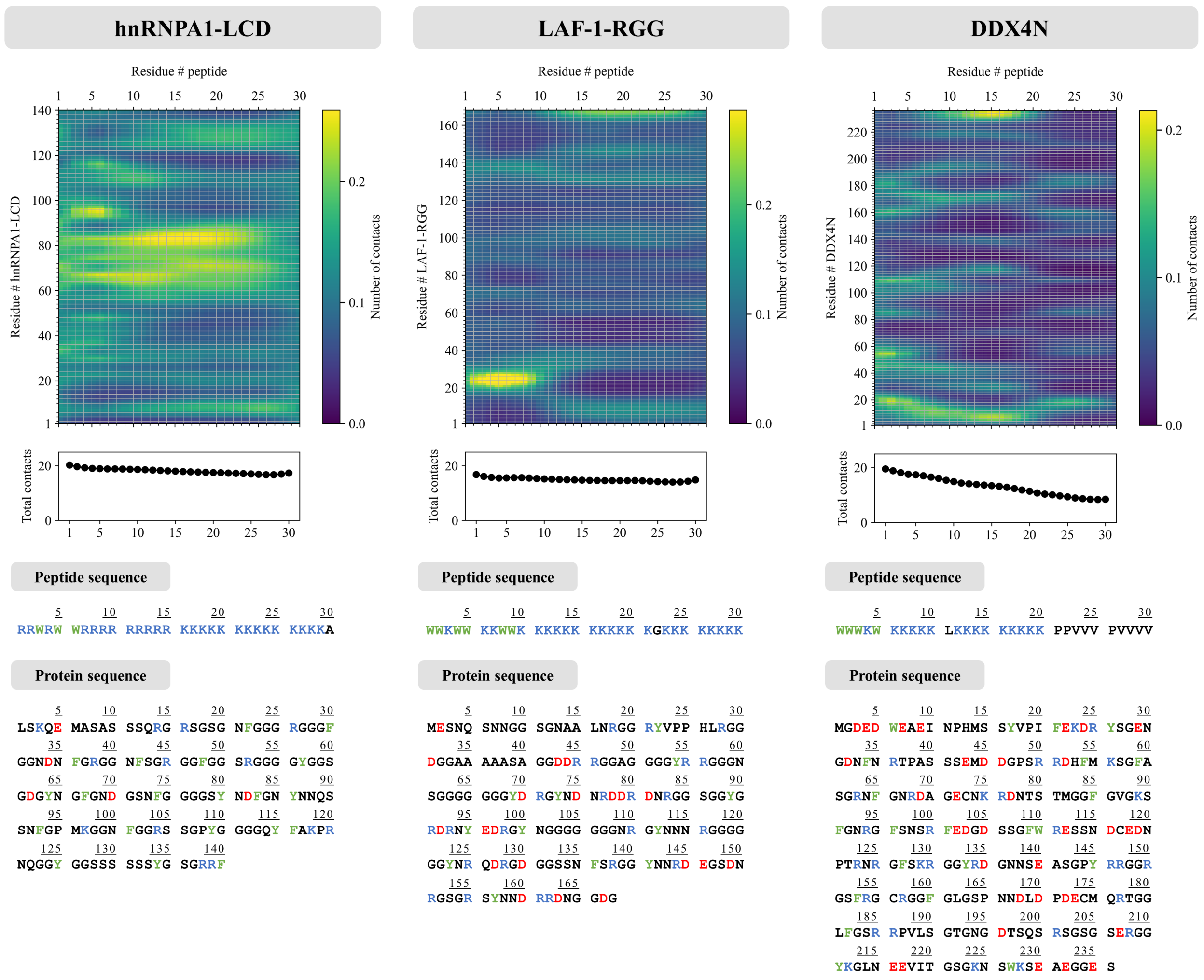

Supplementary Figure 21: Protein–peptide contact maps for the experimentally validated cases, generated using the Martini3-IDP[6] force field.

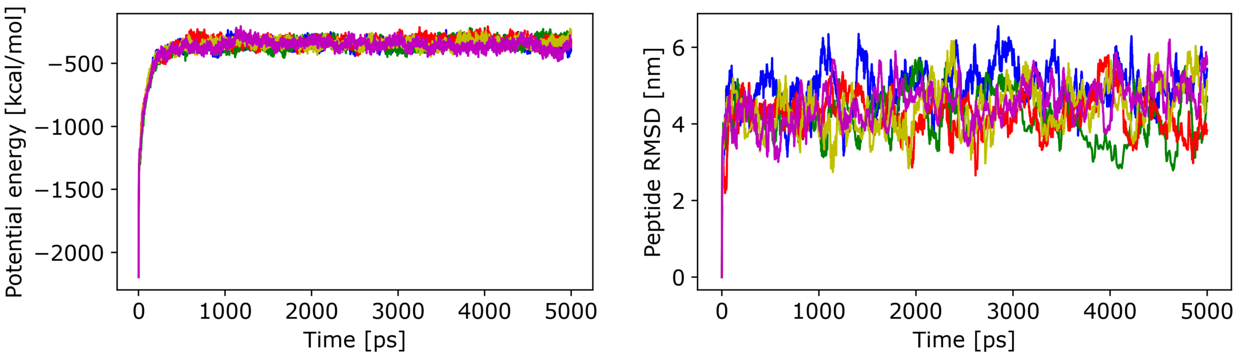

Supplementary Figure 22: Equilibration metrics for 5 examples from the hnRNPA1-LCD initialization, based on total potential energy and deviation of peptide position from initial coordinates.

Protein: hnRNPA1-LCD

Peptide: RRWRWWRRRRRRRRRRKKKKKKKKKKKKKKA

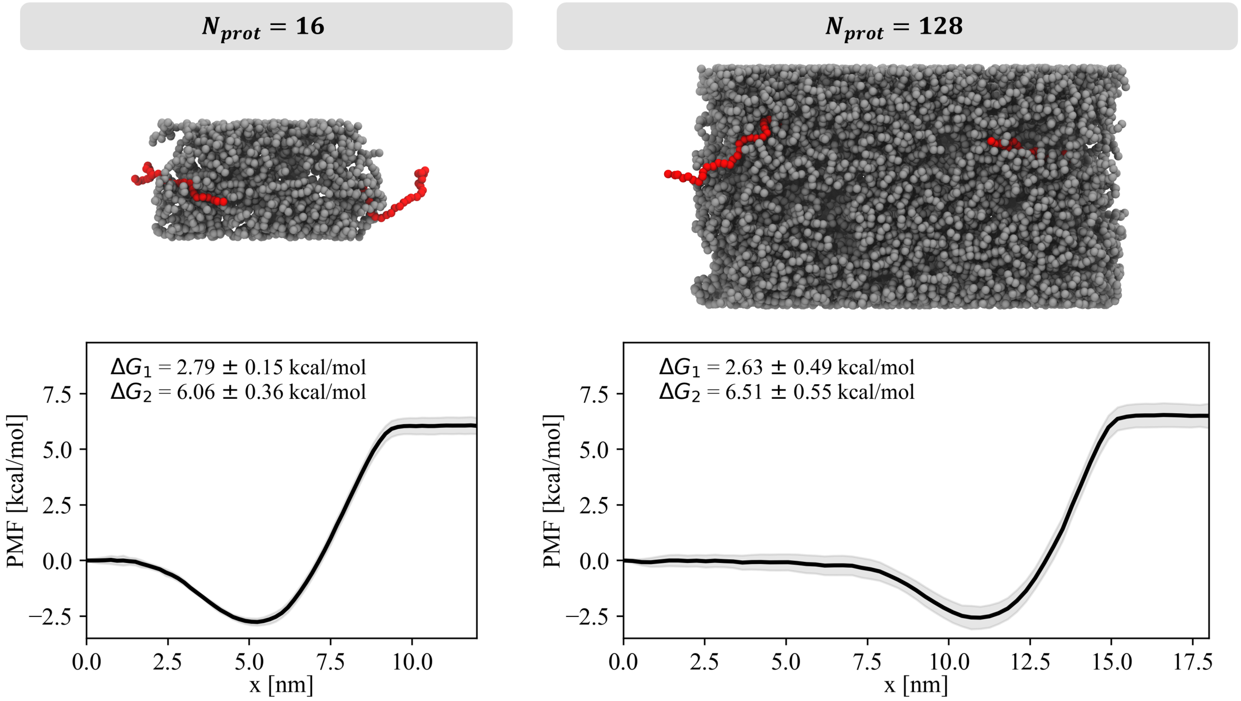

Supplementary Figure 23: Interface partitioning adaptive biasing force simulation results for two different system sizes. x is the distance from the slab center of mass, and the indicated errors correspond to standard deviations (small system: n=3, large system: n=10).

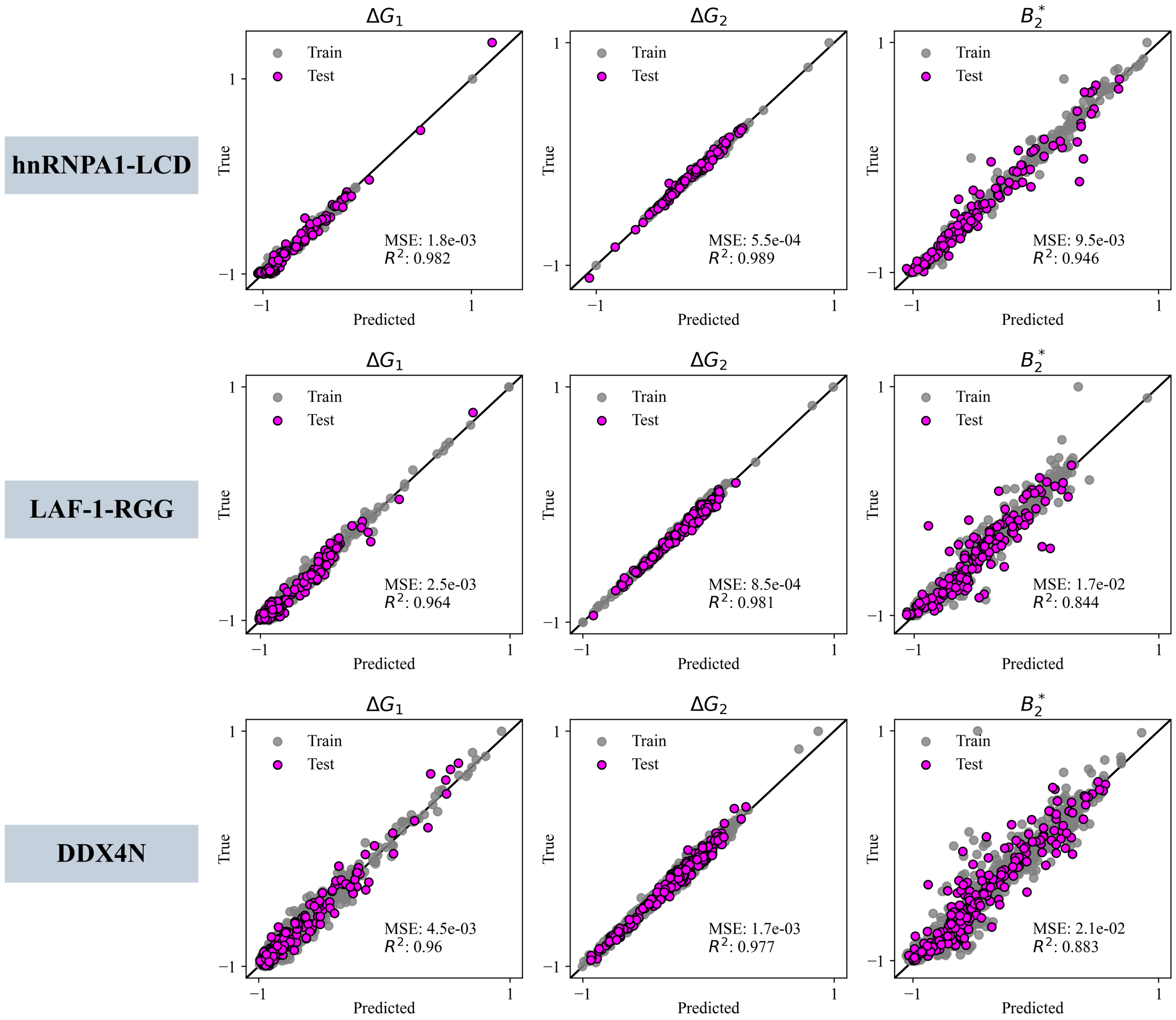

Supplementary Figure 24: Performance of the final model on a 20% test set, evaluated after completing all iterations in each optimization.

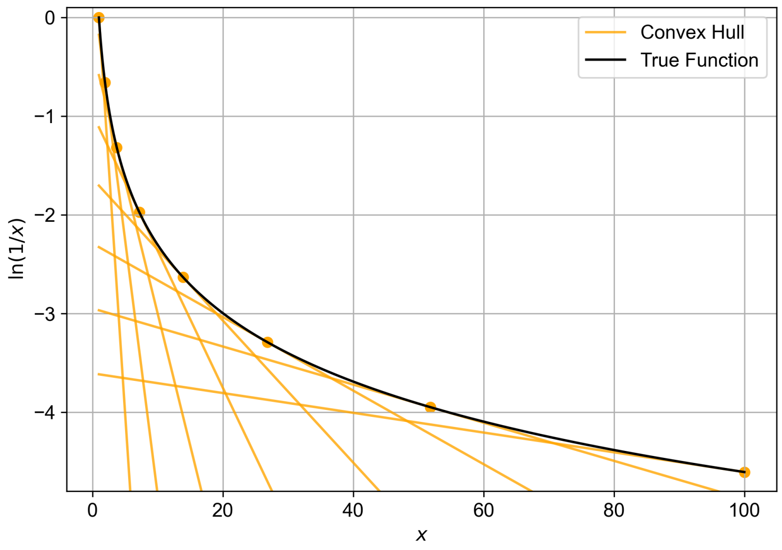

Supplementary Figure 25: Illustration of the convex hull approach.

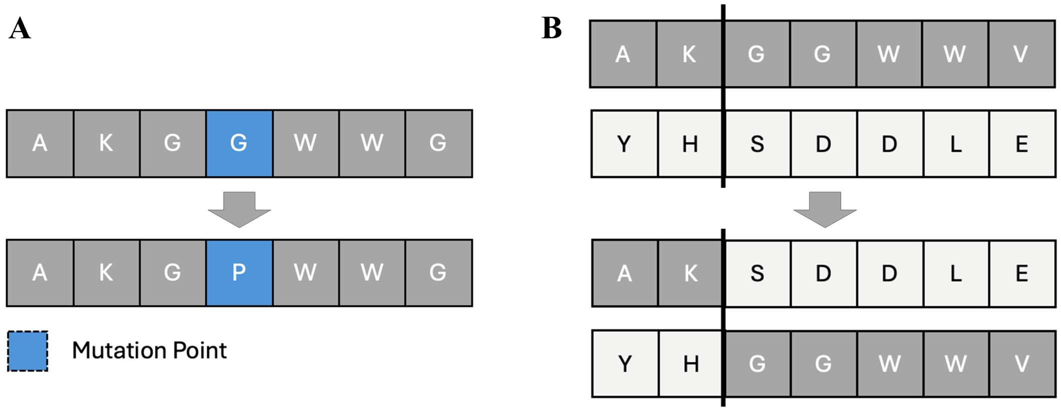

Supplementary Figure 26: Genetic operations employed in the GA were point mutations (A) and crossover events (B).

Supplementary Tables

Supplementary Table 1: Final Pareto front for the hnRNPA1-LCD condensate target.

Sequence	ΔG_1 $[k_{\mathrm{B}}T]$	$\Delta G_2 [k_{\mathrm{B}}T]$	$B_2[\mathrm{nm}^3]$	p^*_{int}	B^*_2
WWWWPWWWWWPWYKKKKKKKKKKKKKKKKK	6.69 ± 0.15	12.75 ± 0.26	-3.2E+05 ± 6.7E+04	6.50E+02	-4.20
WWWWPWWWWYPWWKKKKKKKKKKKKKKKKK	6.40 ± 0.50	12.93 ± 0.66	-2.2E+05 ± 3.1E+04	5.26E+02	-4.03
WWWPWWWWWPYWWKKKKKKKKKKKKKKKKK	6.33 ± 0.46	12.63 ± 0.59	-2.0E+05 ± 2.1E+04	4.74E+02	-3.99
WWWWWDWEWWWWKKKKQKKKKKKKKKKKKK	6.51 ± 0.27	11.13 ± 0.17	-1.1E+05 ± 1.8E+04	3.37E+02	-3.72
WWWYPYWYYPWYWYKKKKKKKKKKKKKKKK	5.66 ± 0.46	12.37 ± 0.41	-6.0E+04 ± 1.2E+04	2.56E+02	-3.48
WWWWPWWWWWKKKWVVKKKKVKKKKKKKKK	6.21 ± 0.22	10.48 ± 0.27	-5.1E+04 ± 1.2E+04	2.08E+02	-3.41
WWWWPWWWWWKKKWVVKKKKKKKKKKKKKK	6.35 ± 0.34	10.31 ± 0.63	-5.0E+04 ± 8.4E+03	1.97E+02	-3.40
WWWWPWWWWWKKKWVVKKKKKKKKKKKKIK	5.99 ± 0.20	10.46 ± 0.54	-4.6E+04 ± 1.0E+04	1.86E+02	-3.36
WWWWPWWWWGWKKWKWKKKKKKKKKKKKKK	5.44 ± 0.32	11.42 ± 0.47	-2.9E+04 ± 7.8E+03	1.84E+02	-3.16
WWWWPWWKWWKKWWKKKKKKKKKKKKKKKK	5.45 ± 0.48	11.25 ± 0.56	-1.1E+04 ± 1.1E+03	1.78E+02	-2.75
WWWWPWWKWKWKWWKKKKKKKKKKKKKKKK	5.40 ± 0.20	11.32 ± 0.41	-7.6E+03 ± 1.0E+03	1.75E+02	-2.58
WWWPWWWKWKKWWWKKKKKKKKKKKKKKKK	5.55 ± 0.37	10.30 ± 0.51	-5.8E+03 ± 5.0E+02	1.38E+02	-2.46
WWWWPWGWWKKKWWWKKKKKKKKKKKKKKK	4.80 ± 0.26	10.06 ± 0.59	-4.7E+03 ± 6.9E+02	7.98E+01	-2.37
RRWRWWRRRRRRRRRKKKKKKKKKKKKKKA	4.67 ± 0.24	10.16 ± 0.61	-1.9E+03 ± 5.7E+02	7.56E+01	-1.97
WRWWRRRRRRRRRRRTKKKKKKKKKKKKKK	4.48 ± 0.45	9.70 ± 0.66	-1.0E+03 ± 2.5E+02	5.72E+01	-1.71
RWRRWWRRRRRRRRRKKKKKKKKKKKKKKA	4.03 ± 0.19	11.16 ± 0.45	-7.8E+02 ± 1.2E+02	5.21E+01	-1.60
WWRRRRRRRRRRRRRKKKKKKKKKKKKKKA	4.10 ± 0.63	10.19 ± 0.65	1.0E+02 ± 6.1E+00	4.90E+01	0.79
RRRRWWRRRRRRRRRKKKKKKKKKKKKAKA	4.26 ± 0.43	9.67 ± 0.43	1.1E+02 ± 8.4E+00	4.88E+01	0.81
WRWRRRRRRRRRRRRRKKKKKKKKKKKKKA	3.92 ± 0.24	10.95 ± 0.34	1.2E+02 ± 2.5E+00	4.62E+01	0.85
WRRRRRWRRRRRRRRKKKKTKKKKKKKKKA	4.41 ± 0.50	9.25 ± 0.74	1.5E+02 ± 4.0E+00	4.61E+01	0.94
RRRRRRRRRRRRRRRRKKKKKKKKKKKKAA	3.69 ± 0.79	9.49 ± 1.43	1.7E+02 ± 2.2E+00	3.08E+01	0.99
RKRRKRRRRRRRRRRRRRKKKKKKKKKKAA	2.42 ± 0.47	8.19 ± 0.78	1.7E+02 ± 2.0E+00	8.54E+00	0.99
RRRRRKKRKRRRRRRRRRRKKKKKKKKKKK	2.42 ± 0.30	7.35 ± 0.45	1.9E+02 ± 3.6E+00	6.52E+00	1.02
RRRRRKKKKKRRRRRRRRRKRRRKKKKKKK	0.67 ± 0.44	6.74 ± 0.62	1.9E+02 ± 1.7E+00	1.59E+00	1.02
RRRRRRRRRRRRRRRRRRRRRRRRRRRRRR	0.15 ± 0.16	35.50 ± 0.71	1.9E+02 ± 2.4E+00	1.16E+00	1.02

Supplementary Table 2: Final Pareto front for the hnRNPA1-LCD condensate target, after applying TANGO[2] and Waltz[3] (threshold=85) aggregation filters.

Sequence	ΔG_1 [$k_\mathrm{B}T$]	ΔG_2[$k_\mathrm{B}T$]	B_2[nm^3]	p^*_{int}	B^*_2
WWWWKKKWWWWWPWWKKKKKKKKKKKKKKKK	5.60 ± 0.24	10.41 ± 0.45	-1.7E+04 ± 2.3E+03	1.49E+02	-2.92
WWWWKKKWWWWWPWKWKKKKKKKKKKKKKKK	5.36 ± 0.51	9.82 ± 0.47	-7.4E+03 ± 1.2E+03	9.86E+01	-2.57
RRWRWWRRRRRRRRRKKKKKKKKKKKKKKA	4.67 ± 0.24	10.16 ± 0.61	-1.9E+03 ± 5.7E+02	7.56E+01	-1.97
WRWWRRRRRRRRRRRTKKKKKKKKKKKKKK	4.48 ± 0.45	9.70 ± 0.66	-1.0E+03 ± 2.5E+02	5.72E+01	-1.71
RWRRWWRRRRRRRRRKKKKKKKKKKKKKKA	4.03 ± 0.19	11.16 ± 0.45	-7.8E+02 ± 1.2E+02	5.21E+01	-1.60
WWRRRRRRRRRRRRRKKKKKKKKKKKKKKA	4.10 ± 0.63	10.19 ± 0.65	1.0E+02 ± 6.1E+00	4.90E+01	0.79
RRRRWWRRRRRRRRRKKKKKKKKKKKKAKA	4.26 ± 0.43	9.67 ± 0.43	1.1E+02 ± 8.4E+00	4.88E+01	0.81
WRWRRRRRRRRRRRRKKKKKKKKKKKKKKA	3.92 ± 0.24	10.95 ± 0.34	1.2E+02 ± 2.5E+00	4.62E+01	0.85
WRRRRRWRRRRRRRRKKKKTKKKKKKKKKA	4.41 ± 0.50	9.25 ± 0.74	1.5E+02 ± 4.0E+00	4.61E+01	0.94
RRRRRRRRRRRRRRRKKKKKKKKKKKKKAA	3.69 ± 0.79	9.49 ± 1.43	1.7E+02 ± 2.2E+00	3.08E+01	0.99
RKRRKRRRRRRRRRRRRRKKKKKKKKKKAA	2.42 ± 0.47	8.19 ± 0.78	1.7E+02 ± 2.0E+00	8.54E+00	0.99
RRRRRKKRKRRRRRRRRRRRKKKKKKKKKK	2.42 ± 0.30	7.35 ± 0.45	1.9E+02 ± 3.6E+00	6.52E+00	1.02
RRRRRKKKKKKRRRRRRRRKRRRKKKKKKK	0.67 ± 0.44	6.74 ± 0.62	1.9E+02 ± 1.7E+00	1.59E+00	1.02
RRRRRRRRRRRRRRRRRRRRRRRRRRRRRR	0.15 ± 0.16	35.50 ± 0.71	1.9E+02 ± 2.4E+00	1.16E+00	1.02

Supplementary Table 3: Final Pareto front for the LAF-1-RGG condensate target.

Sequence	ΔG_1 [$k_\mathrm{B}T$]	ΔG_2[$k_\mathrm{B}T$]	B_2[nm^3]	p^*_{int}	B^*_2
WWWWPWWKKKKKKKKKKKKKKKKKKKKKKK	3.70 ± 0.48	11.36 ± 0.70	-1.1E+03 ± 9.9E+01	3.86E+01	-1.76
WWWWPWKWKKKKKKKKKKKKKKKKKKKKKK	3.47 ± 0.16	11.02 ± 0.33	-4.2E+02 ± 3.5E+01	3.06E+01	-1.35
WWWWPKWKWKKKKKKKKKKKKKKKKKKKGK	3.23 ± 0.37	9.92 ± 0.39	-8.0E+01 ± 1.6E+01	2.25E+01	-0.70
WWWWKKKWWKKVKKVKKVKKKKKKKKKKKK	3.48 ± 0.21	8.68 ± 0.41	4.1E+01 ± 7.6E+00	2.09E+01	0.49
WWWWKKKWWKKKKCKKKKKKKKKKKKKKKK	3.07 ± 0.57	9.29 ± 0.65	6.2E+01 ± 8.0E+00	1.79E+01	0.61
WWKWWKKWWKKKKKKKKKKKKGKKKKKKKK	2.99 ± 0.60	8.87 ± 0.66	1.1E+02 ± 4.4E+00	1.55E+01	0.83
WWKWKKWWWKKKHKKKKKKKKKKKKKKKKK	2.21 ± 0.54	9.72 ± 0.81	1.2E+02 ± 2.7E+00	8.67E+00	0.85
WKWKKKKWWWWKKKKKKKKKKKKKKKKKKK	2.48 ± 0.38	8.06 ± 0.61	1.4E+02 ± 3.1E+00	8.65E+00	0.91
KKKWWKWWKKWWKKKKKKKKKKKKKKKKKK	2.04 ± 0.43	7.65 ± 0.71	1.5E+02 ± 3.5E+00	5.61E+00	0.92
KKKKKWWWKWKKWWKKKKKKKKKKKKKKKK	1.73 ± 0.39	7.60 ± 0.27	1.5E+02 ± 2.9E+00	4.39E+00	0.94
KKKKKKKWWKWKWKWKWKKKKKKKKKKKKK	1.35 ± 0.55	6.33 ± 0.46	1.5E+02 ± 2.3E+00	2.29E+00	0.94
WWEEEEEEEEEEEEEEEDEEEEEEEDDDDD	0.46 ± 0.25	7.51 ± 0.30	1.6E+02 ± 1.7E+00	1.46E+00	0.96
WEEEEEEEEEEEEEEEEEEEEEEEWEEEEE	0.54 ± 0.30	6.71 ± 0.35	1.7E+02 ± 1.9E+00	1.42E+00	0.98
RRRRRRRRRRRRRRKKKKKKRKRRRKKKYK	0.13 ± 0.15	6.41 ± 0.58	1.8E+02 ± 1.4E+00	9.61E-01	1.00
RRRRRRRRKKKKKKKKKKRRRRRRRRRKKK	0.21 ± 0.18	3.55 ± 0.55	1.9E+02 ± 3.1E+00	2.72E-01	1.02

Supplementary Table 4: Final Pareto front for the LAF-1-RGG condensate target, after applying TANGO[2] and Waltz[3] (threshold=85) aggregation filters.

Sequence	ΔG_1 [$k_\mathrm{B}T$]	ΔG_2[$k_\mathrm{B}T$]	B_2[nm^3]	p^*_{int}	B^*_2
WWWPWWWKKKKKKKKKKKKKKKSKKKGKKK	3.46 ± 0.48	11.28 ± 0.43	-8.9E+02 ± 1.3E+02	3.05E+01	-1.66
WWWWKKKWWKKVKKVKKVKKKKKKKKKKKK	3.48 ± 0.21	8.68 ± 0.41	4.1E+01 ± 7.6E+00	2.09E+01	0.49
WWWWKKKWWKKKKCKKKKKKKKKKKKKKKK	3.07 ± 0.57	9.29 ± 0.65	6.2E+01 ± 8.0E+00	1.79E+01	0.61
WWKWWKKWWKKKKKKKKKKKKGKKKKKKKK	2.99 ± 0.60	8.87 ± 0.66	1.1E+02 ± 4.4E+00	1.55E+01	0.83
WWKWKKWWWKKKHKKKKKKKKKKKKKKKKK	2.21 ± 0.54	9.72 ± 0.81	1.2E+02 ± 2.7E+00	8.67E+00	0.85
WKWKKKKWWWWKKKKKKKKKKKKKKKKKKK	2.48 ± 0.38	8.06 ± 0.61	1.4E+02 ± 3.1E+00	8.65E+00	0.91
KKKWWKWWKKWWKKKKKKKKKKKKKKKKKK	2.04 ± 0.43	7.65 ± 0.71	1.5E+02 ± 3.5E+00	5.61E+00	0.92
KKKKKWWWKWKKWWKKKKKKKKKKKKKKKK	1.73 ± 0.39	7.60 ± 0.27	1.5E+02 ± 2.9E+00	4.39E+00	0.94
KKKKKKKWWKWKWKWKWKKKKKKKKKKKKK	1.35 ± 0.55	6.33 ± 0.46	1.5E+02 ± 2.3E+00	2.29E+00	0.94
WWEEEEEEEEEEEEEEEDEEEEEEEDDDDD	0.46 ± 0.25	7.51 ± 0.30	1.6E+02 ± 1.7E+00	1.46E+00	0.96
WEEEEEEEEEEEEEEEEEEEEEEEWEEEEE	0.54 ± 0.30	6.71 ± 0.35	1.7E+02 ± 1.9E+00	1.42E+00	0.98
RRRRRRRRRRRRRRKKKKKKRKRRRKKKYK	0.13 ± 0.15	6.41 ± 0.58	1.8E+02 ± 1.4E+00	9.61E-01	1.00
RRRRRRRRKKKKKKKKKKRRRRRRRRRKKK	0.21 ± 0.18	3.55 ± 0.55	1.9E+02 ± 3.1E+00	2.72E-01	1.02

Supplementary Table 5: Final Pareto front for the DDX4N condensate target.

Sequence	ΔG_1 [$k_\mathrm{B}T$]	ΔG_2[$k_\mathrm{B}T$]	B_2[nm^3]	p^*_{int}	B^*_2
WWWWPWFVKKKKVVVPVVVVPVVVVPVVVV	2.48 ± 0.39	8.89 ± 0.43	-2.1E+03 ± 2.8E+02	1.02E+01	-2.03
WWWWPWKKKKKKKKKPVVVVPVVVVPVVVV	2.36 ± 0.37	9.24 ± 0.29	-3.7E+02 ± 4.5E+01	9.56E+00	-1.29
WWWWPWKKKKKKVKKVKKVVPVVVVPVVVV	2.26 ± 0.44	9.28 ± 0.53	-3.1E+02 ± 3.6E+01	8.81E+00	-1.22
WWWWKKKWKKKKKVKKKKVVPVVVVPVVVV	2.05 ± 0.41	8.63 ± 0.64	-9.2E+00 ± 5.8E+00	6.82E+00	-0.17
WWWWKKKFKKKKKKKKKVKVPVVVVPVVVV	1.96 ± 0.25	8.27 ± 0.50	-6.4E-01 ± 5.1E+00	5.99E+00	-0.01
WWWWKKKKWKKKKKKKKKVVPVVVVPVVVS	1.77 ± 0.23	9.18 ± 0.33	-1.4E-01 ± 7.0E+00	5.56E+00	0.00
WWWWKKKKKKKKKKKKKKKKKPPVVPVVVV	1.68 ± 0.27	8.96 ± 0.44	3.8E+01 ± 4.3E+00	5.03E+00	0.46
WWWWKKKKKKKKKKKKKKKKKVVPVVVSSS	1.50 ± 0.39	9.16 ± 0.57	3.9E+01 ± 3.8E+00	4.26E+00	0.47
WWWKWKKKKKLKKKKKKKKKPPVVVPVVVV	1.80 ± 0.42	7.07 ± 0.32	5.4E+01 ± 2.9E+00	3.99E+00	0.57
WWWWKKKKKKKKKKKKKKPVVVVPVVKVKK	1.43 ± 0.36	9.01 ± 0.58	5.6E+01 ± 4.3E+00	3.97E+00	0.58
WWYWKKKKKKKKKKKKKKPPVVPVVVKKKK	1.47 ± 0.27	7.58 ± 0.43	8.2E+01 ± 3.2E+00	3.56E+00	0.71
DDDDDDDDDWDWWWDFFDDDDDDDDDDDGG	1.09 ± 0.23	11.77 ± 0.63	1.1E+02 ± 1.8E+00	2.96E+00	0.83
DDDDDDDDDDDDWWWWDDDDDDDDDGGGGG	0.87 ± 0.33	9.14 ± 0.50	1.2E+02 ± 2.6E+00	2.33E+00	0.83
DDDDDDDDDWWDWWDDDDDDDDDDDDDDDG	0.83 ± 0.42	9.25 ± 0.55	1.5E+02 ± 2.1E+00	2.24E+00	0.94
EDDDDEDDDDDDDDDDDDYWDYWDDDDDDD	0.46 ± 0.42	7.79 ± 0.94	1.6E+02 ± 3.4E+00	1.48E+00	0.96
KKKKKKKKHKRKKKRRRRRRKKKKKKKKKK	0.48 ± 0.32	7.42 ± 0.44	1.8E+02 ± 2.2E+00	1.47E+00	1.01
RRRRRKKKKKKKKKKKKRRRRRRRKKKKKK	0.23 ± 0.29	13.34 ± 0.54	1.9E+02 ± 4.0E+00	1.26E+00	1.02
RRRRKKKKKKKKKKKRRRRRRRRRRRRKRK	0.20 ± 0.25	17.50 ± 0.77	1.9E+02 ± 2.2E+00	1.22E+00	1.02
RRRRRRRKKKKKKKKKKKKKRKRKRRRRRR	0.18 ± 0.13	15.48 ± 0.32	1.9E+02 ± 2.3E+00	1.19E+00	1.02
RRRRRRRKKKKKKKKKKKKKKKRKRRKRKK	0.13 ± 0.08	10.44 ± 0.42	1.9E+02 ± 2.0E+00	1.13E+00	1.02

Supplementary Table 6: Final Pareto front for the DDX4N condensate target, after applying TANGO[2] and Waltz[3] (threshold=85) aggregation filters.

Sequence	ΔG_1 $[k_{\rm B}T]$	$\Delta G_2 [k_{\rm B}T]$	$B_2[\rm nm^3]$	p^*_{int}	B_2^*
WWWWKKKWKKVKVKKKVKVVPKVVVPVVVV	2.01 ± 0.42	8.04 ± 0.51	-1.3E+01 ± 6.3E+00	6.01E+00	-0.22
WWWWKKKFKKKKKKKKKVKVPVVVVPVVVV	1.96 ± 0.25	8.27 ± 0.50	-6.4E-01 ± 5.1E+00	5.99E+00	-0.01
WWWWKKKKKKKKKKKKKKKKKPPVVPVVVV	1.68 ± 0.27	8.96 ± 0.44	3.8E+01 ± 4.3E+00	5.03E+00	0.46
WWWWKKKKKKKKKKKKKKKKKVVPVVVSSS	1.50 ± 0.39	9.16 ± 0.57	3.9E+01 ± 3.8E+00	4.26E+00	0.47
WWWKWKKKKKLKKKKKKKKPPVVVPVVVV	1.80 ± 0.42	7.07 ± 0.32	5.4E+01 ± 2.9E+00	3.99E+00	0.57
WWWWKKKKKKKKKKKKKKPVVVVPVVKVKK	1.43 ± 0.36	9.01 ± 0.58	5.6E+01 ± 4.3E+00	3.97E+00	0.58
WWYWKKKKKKKKKKKKKKPPVVPVVVKKKK	1.47 ± 0.27	7.58 ± 0.43	8.2E+01 ± 3.2E+00	3.56E+00	0.71
DDDDDDDDDDDDWWWWDDDDDDDDDGGGGG	0.87 ± 0.33	9.14 ± 0.50	1.2E+02 ± 2.6E+00	2.33E+00	0.83
DDDDDDDDDWWDWWDDDDDDDDDDDDDDDG	0.83 ± 0.42	9.25 ± 0.55	1.5E+02 ± 2.1E+00	2.24E+00	0.94
EDDDDEDDDDDDDDDDDDYWDYWDDDDDDD	0.46 ± 0.42	7.79 ± 0.94	1.6E+02 ± 3.4E+00	1.48E+00	0.96
KKKKKKKKHKRKKKRRRRRRKKKKKKKKKK	0.48 ± 0.32	7.42 ± 0.44	1.8E+02 ± 2.2E+00	1.47E+00	1.01
RRRRRKKKKKKKKKKKKKRRRRRRRRKKKKK	0.23 ± 0.29	13.34 ± 0.54	1.9E+02 ± 4.0E+00	1.26E+00	1.02
RRRRKKKKKKKKKKKKRRRRRRRRRRRKRK	0.20 ± 0.25	17.50 ± 0.77	1.9E+02 ± 2.2E+00	1.22E+00	1.02
RRRRRRRKKKKKKKKKKKKKRKRKRRRRRR	0.18 ± 0.13	15.48 ± 0.32	1.9E+02 ± 2.3E+00	1.19E+00	1.02
RRRRRRRKKKKKKKKKKKKKKKKRKRRKRKK	0.13 ± 0.08	10.44 ± 0.42	1.9E+02 ± 2.0E+00	1.13E+00	1.02

Supplementary Methods

Featurization of peptide sequences

Given the relatively limited amount of data (300+ sequences) compared to the massive design space, utilizing engineered descriptors has proven to be an effective approach to represent polymer or protein sequences.[7,8] For the optimization to work with an MILP solver, the featurization has to be a linear mapping of the amino acid sequence (30x20 one-hot matrix) to the feature representation. 44 handcrafted features were used to represent the amino acid sequence in a numerical fashion. These features were not obtained through filtering or feature selection, but were kept as initially defined based on satisfactory performance in initial ML benchmarks (Figure S1). These features were constructed as follows:

- 20 features are designated to represent the composition by quantifying the count of amino acids of type X within the sequence.
- To represent positive and negative charge patterning we utilize zeroth to third moment of the positive and negative charge distribution along the sequence.

$$P_n = \sum_{i=1}^{N} \text{charge}_i^+ * d_i^n \qquad n \in [0, 3] \tag{1}$$

$$N_n = \sum_{i=1}^{N} \text{charge}_i^- * d_i^n \qquad n \in [0, 3] \tag{2}$$

  where d represents the positional distance of residue i from the center of the amino acid sequence, $d_i \in [-14.5, 14.5]$ for the case of a 30 amino acid long peptide. The zeroth moments ($n = 0$) represent the total number of positive and negative charges, respectively. The same concept is used for the distribution of aromatic residues and molecular weight:

$$A_n = \sum_{i=1}^{N} \text{aromatic}_i * d_i^n \qquad n \in [0, 3] \tag{3}$$

$$MW_n = \sum_{i=1}^{N} MW_i * d_i^n \qquad n \in [0, 3] \tag{4}$$

where aromatic$_i$ is a binary variable we define as one for phenylalanine, tryptophan and tyrosine and as zero for all other residues. We also assign the parameter ϵ to each residue quantifying homotypic van der Waals interactions, inspired from the original Mpipi paper:[9]

$$\epsilon = \int_{\sigma}^{3\sigma} \phi(r) dr \tag{5}$$

where $\phi(r)$ is the Wang-Frenkel potential[10] with residue-specific parameters. We then use ϵ to define four more features:

$$E_n = \sum_{i=1}^{N} \epsilon_i * d_i^n \qquad n \in [0, 3] \tag{6}$$

- We also include an adapted sequence hydropathy decoration, SHD, which was adapted from the original SHD[11] due to the absence of a hydropathy value λ in the Mpipi force field:

$$SHD = \sum_{i=1}^{N} \sum_{j=i+1}^{N} (\epsilon_i + \epsilon_j)(j - i)^{-1} \tag{7}$$

- The final features originate from the sequence charge decoration SCD.[12] Due to the non-linear nature of the original version, we defined three similar features that can be utilized in a MILP formulation:

$$SPD = \sum_{i=1}^{N} \sum_{j=i+1}^{N} (\text{charge}_i^+ + \text{charge}_j^+)(j - i)^{-1} \tag{8}$$

$$SND = \sum_{i=1}^{N} \sum_{j=i+1}^{N} (\text{charge}_i^- + \text{charge}_j^-)(j - i)^{-1} \tag{9}$$

$$SPND = \sum_{i=1}^{N} \sum_{j \neq i}^{N} (\text{charge}_i^+ + \text{charge}_j^-)|j - i|^{-1} \tag{10}$$

It is important to note that the one-bead-per-residue representation in Mpipi[9] does not include an explicit peptide backbone or side-chain stereochemistry, meaning that chain directionality is not encoded. Therefore, the simulations are invariant to sequence flipping. To account for this invariance, a preprocessing step was implemented: if the third moment of the molecular weight (MW_3) was negative, the sequence was flipped before computing the descriptor set. In the MILP optimization, a constraint $MW_3 \geq 0$ was added to enforce this condition. This procedure effectively reduced the design space to $\sim 20^{30}/2$. In the final Pareto front, the sequences were again flipped to ensure that the N-terminal aromatic Cy5 dye was attached to the tail prone to condensate interactions.

Benchmarking machine learning models

Using the initial data from the hnRNPA1-LCD optimization, we evaluated the performance of a fully connected two-layer multi-output neural network with varying layer widths and compared it against alternative models, including elastic net (EN), support vector machines (SVM), and gradient-boosted trees (GBT) (Supplementary Figure 1). The predictive performance was evaluated by computing the coefficient of determination (R^2) on 20 randomly generated train-test splits, using an 80/20 split ratio. The neural network training procedure followed the methods described in the main text, with the only difference being the use of fixed values for the initial learning rate (10^{-3}) and weight decay (10^{-3}). A grid search was performed to tune hyperparameters for the SVM and EN models (EN: L1 ratio; SVM: C, ϵ), with each combination evaluated using 5-fold cross-validation. For the gradient boosting regressor, a randomized search over 200 models was conducted to optimize hyperparameters (learning rate, number of estimators, loss function, splitting criterion, minimum samples for splitting, maximum tree depth, and minimum samples per leaf), also assessed through 5-fold cross-validation. All models were implemented using scikit-learn[13] and PyTorch.[14] We observed that the neural network's performance plateaued above 40 nodes per layer and that it generally outperformed elastic net, support vector machines, and gradient-boosted trees. To maintain some flexibility for future data, we chose a layer width of 50 to go forward.

Objective function interface partitioning

We defined the objective of maximizing interface partitioning as maximizing the ratio of the peptide's probability of localizing at the interface p_{int} to the probabilities of localizing in dense p_{den} or dilute phase p_{dil}:

$$\max\left[\frac{p_{\text{int}}}{p_{\text{den}}+p_{\text{dil}}}\right] = \max\left[\frac{p_{\text{den}}}{p_{\text{int}}}+\frac{p_{\text{dil}}}{p_{\text{int}}}\right]^{-1} \tag{11}$$

which can be reformulated using the potential of mean force W as

$$\max\left[\frac{\int_{\text{den}}\exp\left(-\frac{W(q)}{k_{\text{B}}T}\right)dq}{\int_{\text{int}}\exp\left(-\frac{W(q)}{k_{\text{B}}T}\right)dq}+\frac{\int_{\text{dil}}\exp\left(-\frac{W(q)}{k_{\text{B}}T}\right)dq}{\int_{\text{int}}\exp\left(-\frac{W(q)}{k_{\text{B}}T}\right)dq}\right]^{-1} \tag{12}$$

When approximating constant W for dilute and dense phase and interface, and that W_{int} is given by the minimum in the PMF, we can simplify to

$$\begin{aligned}\max\left[\frac{V_{\text{den}}}{V_{\text{int}}}\exp\left(-\frac{W_{\text{den}}-W_{\text{int}}}{k_{\text{B}}T}\right) + \frac{V_{\text{dil}}}{V_{\text{int}}}\exp\left(-\frac{W_{\text{dil}}-W_{\text{int}}}{k_{\text{B}}T}\right)\right]^{-1} \equiv \\ \equiv \max\left[\exp\left(-\frac{\Delta G_1}{k_{\text{B}}T}\right) + \phi\exp\left(-\frac{\Delta G_1+\Delta G_2}{k_{\text{B}}T}\right)\right]^{-1}\end{aligned} \tag{13}$$

where $\phi = V_{\text{dil}}/V_{\text{den}}$ denotes the volume ratio of the dilute to dense phases, which we approximate as 100. This corresponds to a rough estimate and therefore carries high uncertainty. Initialization results for the hnRNPA1-LCD system suggested that the first term containing ΔG_1 is more challenging to minimize, as in the top 5 % of initialization sequences the first term accounts for 94 ± 5 % (mean ± SD, n=30) of the sum. Given the high uncertainty in the ϕ parameter, we intentionally overestimated the second term containing ΔG_2 as a safeguard against underestimating ϕ, leading to the final objective to be maximized:

$$p^*_{\text{int}} = \left[\exp\left(-\frac{\Delta G_1}{k_{\text{B}}T}\right) + \phi\exp\left(-\frac{\Delta G_2}{k_{\text{B}}T}\right)\right]^{-1} \tag{14}$$

After this approximation, the first term still accounts for 93 ± 6 % of the sum for the top 5 % (measured by p^*_{int}) of initialization sequences. In order to reduce skewedness and associated numerical issues, we minimized $\ln(1/p^*_{\text{int}})$, which is an equivalent optimization problem to maximizing p^*_{int}. $\ln(1/p^*_{\text{int}})$ is also still a convex function with respect to the variables ΔG_1 and ΔG_2, which can be demonstrated by applying Sylvester's criterion.[15] In other words, let the objective function $f : \mathbb{R}^2 \to \mathbb{R}$ with variables x and y be

$$f(x, y) = \ln\left(\exp(-x) + \phi \exp(-y)\right) \quad x, y \in \mathbb{R},\ \phi \in \mathbb{R}_{>0}$$

We can then calculate the Hessian matrix (H), all principal minors (a_{11} and a_{22}), and the determinant ($\det(H)$):

$$\begin{aligned}
H &= \begin{bmatrix} \frac{\exp(-x)}{\exp(-x)+\phi\exp(-y)} - \frac{\exp(-2x)}{(\exp(-x)+\phi\exp(-y))^2} & -\frac{\phi\exp(-x-y)}{(\exp(-x)+\phi\exp(-y))^2} \\ -\frac{\phi\exp(-x-y)}{(\exp(-x)+\phi\exp(-y))^2} & \frac{\phi\exp(-y)}{\exp(-x)+\phi\exp(-y)} - \frac{\phi^2\exp(-2y)}{(\exp(-x)+\phi\exp(-y))^2} \end{bmatrix} \\
&= \begin{bmatrix} a_{11} & a_{12} \\ a_{21} & a_{22} \end{bmatrix} \\
a_{11} \geq 0 &\iff \frac{\exp(-x)}{\exp(-x) + \phi\exp(-y)} \geq \frac{\exp(-2x)}{(\exp(-x) + \phi\exp(-y))^2} \\
&\iff \exp(-y) \geq 0 \quad \forall\{x, y\} \in \mathbb{R} \\
a_{22} \geq 0 &\iff \exp(-x) \geq 0 \quad \forall\{x, y\} \in \mathbb{R} \\
\det(H) &= a_{11}a_{22} - a_{12}a_{21} = 0 \quad \forall\{x, y\} \in \mathbb{R}
\end{aligned}$$

Because all principal minors and the determinant of the Hessian are non-negative, the matrix is positive semi-definite, and therefore the objective function f is convex. This convexity allowed to approximate the objective function by adding linear first order Taylor approximations to the MILP, the principle is illustrated in Supplementary Figure 25 for a univariate function.[16] The linearization was performed in a gridwise fashion for 30 points in both ΔG_1 and ΔG_2 (S_1 and S_2, respectively), ranging from the smallest value to the largest value

obtained in the training data, resulting in a total of 900 supporting planes. For a single point $s_1 \in S_1$ and $s_2 \in S_2$, we introduced the following constraint for the linearized interface partitioning objective ($\ell \approx \ln(1/p^*_{\text{int}})$) to be minimized:

$$\begin{aligned}\ell \geq \ln & \left[\exp\left(-\frac{s_1}{k_\text{B}T}\right) \; + \; \phi\exp\left(-\frac{s_2}{k_\text{B}T}\right)\right] \\ & - \; \frac{\exp\left(-\frac{s_1}{k_\text{B}T}\right)}{\exp\left(-\frac{s_1}{k_\text{B}T}\right) \; + \; \phi\exp\left(-\frac{s_2}{k_\text{B}T}\right)}\frac{1}{k_\text{B}T}\left(\Delta G_1 \; - \; s_1\right) \\ & - \; \frac{\phi\exp\left(-\frac{s_2}{k_\text{B}T}\right)}{\exp\left(-\frac{s_1}{k_\text{B}T}\right) \; + \; \phi\exp\left(-\frac{s_2}{k_\text{B}T}\right)}\frac{1}{k_\text{B}T}\left(\Delta G_2 \; - \; s_2\right)\end{aligned} \tag{15}$$

By adding this constraint for all combinations of s_1 and s_2, the nonlinear objective (14) was reformulated to be compatible with mixed-integer linear programming, such that:

$$\max \; p^*_{\text{int}} \; \approx \; \begin{array}{ll} \min & \ell \\ \text{s.t.} & \text{Eq (15)}, \quad \forall s_1 \in S_1, s_2 \in S_2 \end{array} \tag{16}$$

Notice that, minimizing ℓ combined with the direction of the inequality ($\geq$) in (15) favors the constraint to be active and, therefore, enforces the linear approximation without the need for equality constraints.

Integration of AGGRESCAN predictor

We incorporated AGGRESCAN as a constraint in the MILP optimization because its prediction algorithm is based on linear operations, making it suitable for integration into an MILP. For all details about the algorithm, we refer to the original publication.[17] AGGRESCAN calculations are based on aggregation-propensity values per amino acid (aaAV, or a3v). The a3v is averaged with a sliding window of length 5 given the sequence length of 30, resulting in an a4v value assigned to the central residue in each window. If there are 5 or more sequential residues with an a4v larger than the hot spot threshold (HST$= -0.02$) and none of the amino acids inside this window is a proline, the sequence contains an aggregation hot spot and is thus defined as infeasible in the MILP. To formulate this, one has to find a suitable linearization for finding the minimum of a list of 5 values, which should not exceed the HST. This was achieved by introducing big-M constraints. We consider the following equation:

$$X = \min\{x_1, ..., x_n\} \tag{17}$$

For this we have to introduce n binary variables z_i which are equal to 0 if the value at position i is the minimum and 1 otherwise. We also introduce a parameter M which has to be larger than the largest value inside the list, but as small as possible. We can then introduce the following constraints:

$$X \geq x_i - M * z_i \qquad i \in n \tag{18}$$

$$X \leq x_i \qquad i \in n \tag{19}$$

$$\sum_i^n z_i = n - 1 \tag{20}$$

These constraints were implemented for each window combined with $X \leq$ HST, ensuring that the minimal value obtained was less than or equal to the HST. If a window contained

a proline, we ensured that X was lower than the HST by subtracting 7, always leading to $X <$ HST.

Comparing MILP and genetic algorithm

Mixed-integer linear programming (MILP) can generate the true Pareto front, whereas genetic algorithms (GA) risk becoming trapped in local optima. To investigate this in our optimization case, we constructed the Pareto front using trained neural networks for the first two iterations with hnRNPA1-LCD as a condensate target. As a genetic algorithm, we employed NSGA-II,[18] utilizing genetic operations at the sequence level (Supplementary Figure 26):

- Point mutations randomly replace a single amino acid with any other amino acid, each with equal probability.
- Crossover events exchange subsequences between sequences at a randomly chosen split position.

NSGA-II was implemented using a generation size of 200. The probabilities for both point mutation and crossover event were set to 50%. As an initial population, the 300 random sequences from the initialization were used. The AGGRESCAN predictor was implemented as a constraint in the following manner: if a genetic operation lead to a constraint violation, the sequence was discarded and a new sequence is generated. This ensured that no aggregation-prone sequence was produced at any point. The genetic algorithm was run until the hypervolume of the Pareto front stagnated.

To compare the ability of GA and MILP to generate Pareto fronts, we applied both methods to two trained surrogate models: one based on the initialization data (used for iteration 1) and another after one additional iteration. It is important to note that no actual iterations were performed using the GA; only the surrogate models at these stages were optimized. While the genetic algorithm reconstructed most of the true Pareto front identified by MILP, it failed to capture a significant portion with high B_2^* values in the iteration 1 model (Supplementary Figure 2). Although the lack of hypervolume improvement with increasing generations suggested convergence, the GA was in fact trapped in a local optimum. This

highlights the superior performance of MILP for this optimization problem.

References

(1) Shannon, C. E. A mathematical theory of communication. *The Bell System Technical Journal* **1948**, *27*, 379–423.

(2) Fernandez-Escamilla, A. M.; Rousseau, F.; Schymkowitz, J.; Serrano, L. Prediction of sequence-dependent and mutational effects on the aggregation of peptides and proteins. *Nature Biotechnology* **2004**, *22*, 1302–1306.

(3) Maurer-Stroh, S.; Debulpaep, M.; Kuemmerer, N.; Paz, M. L. D. L.; Martins, I. C.; Reumers, J.; Morris, K. L.; Copland, A.; Serpell, L.; Serrano, L.; Schymkowitz, J. W.; Rousseau, F. Exploring the sequence determinants of amyloid structure using position-specific scoring matrices. *Nature Methods* **2010**, *7*, 237–242.

(4) Rubinstein, M.; Colby, R. H. *Polymer Physics*; Oxford University Press, 2003.

(5) Tesei, G.; Lindorff-Larsen, K. Improved predictions of phase behaviour of intrinsically disordered proteins by tuning the interaction range [version 2; peer review: 2 approved]. *Open Research Europe* **2023**, *2*.

(6) Wang, L.; Brasnett, C.; Borges-Araújo, L.; Souza, P. C. T.; Marrink, S. J. Martini3-IDP: improved Martini 3 force field for disordered proteins. *Nature Communications* **2025**, *16*, 2874.

(7) Patel, R. A.; Webb, M. A. Data-Driven Design of Polymer-Based Biomaterials: High-throughput Simulation, Experimentation, and Machine Learning. *ACS Applied Bio Materials* **2024**, *7*, 510–527.

(8) Patel, R. A.; Borca, C. H.; Webb, M. A. Featurization strategies for polymer sequence or composition design by machine learning. *Molecular Systems Design and Engineering* **2022**, *7*, 661–676.

(9) Joseph, J. A.; Reinhardt, A.; Aguirre, A.; Chew, P. Y.; Russell, K. O.; Espinosa, J. R.; Garaizar, A.; Collepardo-Guevara, R. Physics-driven coarse-grained model for biomolecular phase separation with near-quantitative accuracy. *Nature Computational Science* **2021**, *1*, 732–743.

(10) Wang, X.; Ramírez-Hinestrosa, S.; Dobnikar, J.; Frenkel, D. The Lennard-Jones potential: when (not) to use it. *Phys. Chem. Chem. Phys.* **2020**, *22*, 10624–10633.

(11) Zheng, W.; Dignon, G.; Brown, M.; Kim, Y. C.; Mittal, J. Hydropathy Patterning Complements Charge Patterning to Describe Conformational Preferences of Disordered Proteins. *The Journal of Physical Chemistry Letters* **2020**, *11*, 3408–3415.

(12) Firman, T.; Ghosh, K. Sequence charge decoration dictates coil-globule transition in intrinsically disordered proteins. *The Journal of Chemical Physics* **2017**, *148*, 123305.

(13) Pedregosa, F. et al. Scikit-learn: Machine Learning in Python. *Journal of Machine Learning Research* **2011**, *12*, 2825–2830.

(14) Paszke, A. et al. *Advances in Neural Information Processing Systems 32*; Curran Associates, Inc., 2019; pp 8024–8035.

(15) Gilbert, G. T. Positive Definite Matrices and Sylvester's Criterion. *The American Mathematical Monthly* **1991**, *98*, 44–46.

(16) Marler, R. T.; Arora, J. S. Survey of multi-objective optimization methods for engineering. *Structural and Multidisciplinary Optimization* **2004**, *26*, 369–395.

(17) Conchillo-Solé, O.; de Groot, N. S.; Avilés, F. X.; Vendrell, J.; Daura, X.; Ventura, S. AGGRESCAN: A server for the prediction and evaluation of "hot spots" of aggregation in polypeptides. *BMC Bioinformatics* **2007**, *8*.

(18) Deb, K.; Pratap, A.; Agarwal, S.; Meyarivan, T. A fast and elitist multiobjective genetic

algorithm: NSGA-II. *IEEE Transactions on Evolutionary Computation* **2002**, *6*, 182–197.
