# Supplementary material for: De novo design of peptides localizing at the interface of biomolecular condensates
Source: Nat Commun. 2026 May 16;17:6497. doi: 10.1038/s41467-026-73099-9 (PMC13376736; doi:10.1038/s41467-026-73099-9)
Supplement: Supplementary file 3 — Transparent Peer Review file [file 41467_2026_73099_MOESM3_ESM.pdf]

nature portfolio

Peer Review File

De novo design of peptides localizing at the interface of biomolecular condensates

Corresponding Author: Professor Paolo Arosio

This file contains all reviewer reports in order by version, followed by all author rebuttals in order by version.

Version 0:

Reviewer comments:

Reviewer #1

(Remarks to the Author)
Schneider et al. report on an approach to de novo design peptide sequences that seek the interface of biomolecular condensates using a computational loop consisting of coarse-grained simulations, machine learning, mixed-integer linear programming followed by in vitro experimental validation. Interfaces of water-rich, charged and dynamic liquid-liquid phase separating proteins are a challenging target with previous work focusing on identifying proteins that are capable of partitioning to the phase boundary. The ability to select for short-, synthesizable- peptides from a vast design space, is a potential key in understanding these surfaces in depth, as well as controlling the properties and function of the underlying biomolecular condensates. Towards this goal, this manuscript presents an approach rooted in using two distinct classes of CG-MD simulations to identify peptides that (i) have strong tendencies for interfacial localization using slab simulations with adaptive biasing force and (ii) those that demonstrate weak homotypic interactions to disfavor aggregation using a simulation of peptide dimers. Peptide sequences are then subjected to a machine learning and optimization pipeline to maximize the interface seeking propensity while minimizing the self-interactions in a loop with the molecular simulations. Predicted sequences are identified to be amphiphilic in nature, with aromatic rich ends preferring the interface, and charged domains pointing into the bulk. Using fluorescently labelled peptides identified using this approach, the authors demonstrate strong preferential interface seeking behavior of these molecules, with the ability to influence the size/charge of the biomolecular condensates. Although this work presents an interesting approach to design peptides with the ability to seek the biomolecular condensate interface, several conceptual questions arise which should be addressed prior to being considered for publication –
1) The introduction prefaces the manuscript by presenting the idea that water- and ion-rich condensates are challenging to target using traditional surfactant molecules unlike, for example, oil-in-water emulsions. The peptides identified in this work however appear to have strong surfactant-like tendencies with amphiphilic peptide designs. Could the authors discuss why they believe that the peptide designs are distinct from surfactants that seek interfaces?
2) Following up on the previous point, it would be prudent to discuss the enrichment of tryptophans at the C-terminal ends of the designed peptides vs. other aromatics such as phenylalanine, tyrosine or histidine in all three condensate systems evaluated in this manuscript. It has been previously reported that in case of transmembrane proteins, tryptophan residues are preferentially enriched near bilayer interfaces (Commun Chem 6, 154 (2023). https://doi.org/10.1038/s42004-023-00953-y). Furthermore, recent work on short peptides containing lysine and tryptophan residues have been shown to themselves undergo liquid-liquid phase separation and have strong tendencies to stabilize protein interfaces (Nat. Mater. (2025). https://doi.org/10.1038/s41563-025-02300-z). Do the authors propose a similar special role for tryptophan in stabilizing condensate interfaces that is selected for by the computational pipeline?
3) The Mpipi forcefield employs an implicit solvation model to introduce the effects of water. The lack of explicit water may potentially lead to generation of a more distinct interface in the molecular simulations between the condensate protein rich phase and poor phase leading to a strong free energy bias in the adaptive biasing force simulations. Would a more diffuse interface enabled by having explicit solvent influence the peptide sequences identified computationally?
4) The pipeline emphasizes the use of tools such as WALTZ, AGGRESCAN and TANGO to identify and remove sequences with the potential of undergoing aggregation. Several of these tools are biased towards beta-sheet rich sequences derived from or having analogy to amyloid proteins. Could this be a potential drawback as some amyloid like sequences can be amphiphilic and interface seeking but would likely be eliminated by the computational workflow?
5) The adaptive biasing force simulations generate PMF curves using a simulation of one peptide chain from the condensate proteins. Interface seeking peptides would also likely self-interact or be in close proximity at the interface and with charged (lysine rich tails) have high repulsion and therefore lower interface loading. It would therefore be useful in future work, to also

account for lateral interactions between interface seeking peptides and design in some favorable self-interactions.
6) The amphiphilic peptide sequences designed in this work, although having been through a pipeline to eliminate self-aggregation, are likely to undergo self-assembly. The authors also report the presence of nanosized clusters using dynamic light scattering analysis in Figure S10. Is it therefore a strictly necessary condition to minimize and/or eliminate peptide self-aggregation as a criterion for designing biomolecular condensate interface seeking peptides?
7) Could the authors comment on whether any potential reorganization of the condensate forming protein chains is observed upon the interfacial binding of the peptides? The contact map in Figure 3B is interesting and highlights the importance of cation-pi type contacts between the peptide and hnRNPA1-LCD protein condensate. However, it would also be insightful to comment on the distribution of condensate residues at the interface in the absence and presence of the peptide, particularly in the local proximity to the peptide contacts.

(Remarks on code availability)

Reviewer #2

(Remarks to the Author)
In the manuscript "De novo design of peptides localizing at the interface of biomolecular condensates" submitted by T. Schneider et al., a computational workflow is presented with the goal of predicting new peptides at the interface with biomolecular condensates. The work is incredibly well written, making it very readable for a broad audience, particularly those who are interested in interfacial polymer science and peptide/protein design in the modern era of machine learning and artificial intelligence. The work is complemented with experimental validation of a few of the discovered peptides. One of the major claims stated in the abstract and introduction of the manuscript, however, may not be satisfied. The authors state on Line 9, Page1 “The molecular grammar driving the preferential localization of molecules at condensate interfaces remains largely unknown.” It is very possible that the use of the coarse grained force field Mpipi biased the results towards Arginine/Tryptophan rich results as the original paper describing Mpipi states, “ The model is parameterised from both atomistic simulations and bioinformatics data and accounts for the dominant role of π–π and hybrid cation-π/π–π interactions and the much stronger attractive contacts established by arginines than lysines.” If one were to use a more popular coarse grained force field like MARTINI, what would the results look like? Regardless of the bias towards these interactions, the authors have developed a workflow that identifies several peptides with predicted interfacial localization. I would recommend this work to Nature Communications if the authors addressed the issue of molecular biasing with the Mpipi coarse grained force field and refine the identified “molecular grammar” addressing some of the concerns below.

Bulleted comments:
1. The workflow (pp.5-7) is incredibly well described for a broad audience. The definition of the two important parameters utilized (probability of localization at the interface, p(int), and homotypic interactions, B2) are clearly explained. And defining Pareto solutions as “a set of globally optimal, non-dominated sequences that cannot improve one objective function without compromising the other one” is clear and succinct. These clear definitions demonstrate the authors’ excellent communication throughout the presented work.
2. The authors explain Mpipi as their force field choice on p. 7 line 132. “ a one-bead per residue mode, due to its physics-based parametrization strategy and ability to explain experimental data of disordered proteins.” This is well justified for the topic, but this reviewer would be curious to hear the thoughts of the authors regarding the possibility that Mpipi may have skewed the peptide design towards the favorable R/W interactions. If one were to plot a histogram of the amino acid content within the peptides predicted, the bias towards R/W/K would be clear.
3. Please provide the sequences (in the supporting information) of the biomolecular condensates used: hnRNPA1, LAF-1-RGG, and DDX4N. From the simulation results of the condensates, can the authors please present a color-coded model from their simulations indicating the charged, aromatic, and aliphatic amino acids. This would help the reader understand where the potential interactions might occur. For example, are the aliphatic residues buried on the interior of the condensates?
4. The production of the contact map (Figure 3B) highlights that there are favorable R/W interactions between the peptide (“Peptide 2”) and hnRNPA1. These results are further supported by the experiments in Figure 4. However, when presenting the interface of peptides with LAF-1-RGG and DDX4N negatively charged peptides are suggested interfacial peptides. However, the peptides that were chosen for experiments for both LAF-1-RGG and DDX4N are incredibly similar to that investigated with hnRNPA-1, i.e. aromatic + lysine. Therefore, it seems that the authors’ choice biased the “molecular grammar”. The authors conclude on p. 18 that the following physicochemical traits are common for all discovered peptides: surfactant-like, aromatic rich, and matched net charge.
a. Surfactant-like: This seems to be consistent with the findings.
b. Aromatic Rich: this is potentially biased by the use of Mpipi.
c. Matched net charge: not supported by experimental work.
i. No experimental evidence was presented indicating that negatively charged sequences do or do not yield interface partitioning.
ii. The valine rich segments of peptide 2 might embed in the DDX4N interior if the hydrophobic residues of the condensate are buried. The many lysine residues most likely remain outside of the condensate.
5. Contact maps were not generated for these interactions: LAF-1-RGG/Pep2 and DDX4N/Pep2. They would help support some of the “molecular grammar” claims.
6. Contact maps generated from other force fields would strengthen the molecular grammar arguments, but if the use of Mpipi is intentional to force those strong pi-pi interactions as a dominant design feature, then that needs to be clearly stated.

(Remarks on code availability)

Reviewer #3

(Remarks to the Author)
The manuscript “De novo design of peptides localizing at the interface of biomolecular condensates” by Timo Schneider and co-workers presents a computational pipeline starting from coarse-grained MD simulations and an active learning loop to optimize peptide sequences for their ability to localize at the interface of biomolecular condensates. The authors use this pipeline to design interface-localizing peptides for three condensate systems, hnRNPA1, LAF1 and Ddx4. They synthesized one selected Pareto-optimal peptide sequence for each system, and showed that these selected peptides indeed localized to the interface of their respective condensate systems, and that it results in a smaller average condensate size compared to no or a random peptide.

Understanding the interfacial properties of condensates is of significant importance, given the recent examples in which condensate interfaces play an important role: amyloid formation of client proteins and the liquid to solid transition of protein droplets and condensate interface potentials that can promote redox reactions. The manuscript provides a new approach to design peptides that specifically localize to the interface and potentially change condensate interfacial properties, which may also help to gain a better understanding of the (dynamic) nature of the condensate interface. In light of this novel approach to design potentially relevant peptides that may advance our understanding of condensates, and the current interest in condensates from a biological point of view, this manuscript is likely of interest to the community and readership of Nature Communications. However, the authors should address the following serious concerns before the work can be considered for publication.

Major:
1. The novelty of this work in relation to other recent papers on the interfacial localization of proteins or peptides on condensates should be more explicitly mentioned. In particular, the sentence “However, to our knowledge preferential interfacial localization has not been demonstrated for small molecules or peptides.” (p.3) should be revised. There have been several recent manuscripts describing peptide derivatives, rationally designed polymers, and dendrimers that preferentially localize to the interface of condensates or coacervates.
- https://www.nature.com/articles/s41557-025-01800-4
- https://pubs.acs.org/doi/full/10.1021/jacs.5c03740
- https://pubs.acs.org/doi/10.1021/jacs.5c09772
- https://pubs.acs.org/doi/10.1021/acsnano.4c16908
- https://www.sciencedirect.com/science/article/pii/S0021979725020004
- https://pubs.acs.org/doi/10.1021/acs.langmuir.5c03118
Moreover, there is also an entire body of work on stabilization of such droplets by membranes assembled from small amphiphilic molecules, including fatty acids and phospholipids. These should be considered small molecules. Examples include:
- https://www.nature.com/articles/nchem.1921
- https://pubs.acs.org/doi/10.1021/jacs.0c12494
- https://advanced.onlinelibrary.wiley.com/doi/full/10.1002/advs.202412312
- https://www.biorxiv.org/content/10.1101/2021.02.19.432011v3
and many more.
Some of these works have explicitly also looked at the effects of amphiphiles on droplet size.
I do not think all these papers have to be cited, but it is important to place the current work in the right literature context.

2. My main issue with the MD part of the workflow is that the one-bead per residue model is an extremely crude, oversimplified representation of the true interactions between peptides. Combined with the fact that the simulation only contains 16 protein copies, I am doubtful that this model can accurately capture the chemical nature of the different condensate systems, let alone the interface. When looking at the snapshots in Figure 3b for example, every protein is both in the bulk and at the interface, and the simulated peptide will almost always be "at the interface", simply because the system is so small. This point is salient, because the peptides that come out of this pipeline have remarkably low sequence complexity, but the authors derive "key physicochemical design parameters" from the predicted sequences. One may even ask if these sequences could not have been simply designed by hand. In particular, two questions arise here: is the system size large enough to accurately determine the relative size of the aromatic-rich block and the charge-dense block? And secondly, is the one-bead per residue model accurate enough to find realistic and diverse sequences especially for the water-soluble block. For example, why does this block contain exclusively charged residues? Considering the effects of counterion condensation, there should be no hardly any difference between 1 charge per amino acid, or 1 per 2. Oligovalines can easily form beta sheet structures.

3. In addition, the machine learning and optimization pipeline contains many choices that are not justified or adequately explained. For example:
- What is acceptable statistical error (p. 20)? Compared to what?
- How was the equilibration time for the simulations determined (5 ns, 30 ns)?
- How was the set of sequence features chosen?
- What is the balance between exploration and sampling relevant regions of the design space (p.23). How was this

determined?
- What is the justification for choosing 25 exploration points at iterations 3 and 4, and zero for the last three iterations?
- Why are the results of the simulations invariant to sequence flipping due to the coarse-grained representation? (SI p.3)
- Why was the second term intentionally overestimated? (SI p.7). Does this give rise to bias in the sequence?
- In the second line of equation 13 (SI p.7), the prefactor (V_den/V_int) is apparently set to 1. What is the justification for this?

4. The manuscript lacks a functional characterization of the synthesized interfacially localizing peptides. The introduction suggests that this pipeline is aimed at targeted modulation of the interface of condensates without perturbing their interior, but the authors have not quantitatively compared the condensate interior properties (e.g., viscosity, density) with and without the peptide; and the surface tension or surface charge with and without the peptide. An alternative proof of targeted modulation could be a suppression of interfacial aggregation in the case of hnRNPA1, or an altered redox activity.

Minor:
5. “Moreover, a high-throughput experimental strategy for extensive screening is still lacking.” (p.4). This manuscript does not provide a high-throughput experimental strategy.
6. Line 73 and 75: “dynamics” is missing after “molecular” (2x)
7. Figure 4c and Figure S12: what is the blurry dark ring in the center of the field of view in the brightfield images?
8. Figure 5d and Figure S8: why do not all condensates exhibit complete surface coverage? Are the systems not in equilibrium?
9. Figure S10: should the labels be inverted? I would expect the condensates with the peptide to have a smaller average size.

(Remarks on code availability)
Code was not available in the reviewer files.

Version 1:

Reviewer comments:

Reviewer #1

(Remarks to the Author)
In the revised manuscript, Schneider et al. have largely addressed the concerns raised previously. Using dye labelled peptides and FCS analyses, the authors have shown that identified peptide sequences have poor homotypic interactions at evaluated concentrations, making them distinct from typical surfactant architectures.

Using a higher resolution CG FF such as MARTINI3-IDP, the authors have validated some of the previously identified molecular designs for interface localization and demonstrated reasonable agreement with the one-bead-per-residue models. One minor concern I have is regarding response number 3 - "In the case of Martini3-IDP, interfacial localization was clearly confirmed for two peptides." The density profiles reported in Figure S23, appear to show only for the DDX4N case that the peptide is partly in the protein-rich as well as water-rich phases. In the other two, the peptide appears to be sequestered away from the interface. Which two peptides are the authors referring to here?

Additionally, by evaluating simulations with varying number of peptide chains, the authors demonstrate that the distribution of condensate residues at the interface is not a given upon localization. In light of these additional simulations, experiments and analyses, I recommend the manuscript for publication.

(Remarks on code availability)

Reviewer #2

(Remarks to the Author)
The authors have made an exceptional effort addressing all reviewers concerns and criticism. Of particular note the additional experimental validation and further simulation efforts that apply force fields, CALVADOS 2 and MARTINI3 IDP, to address any concerns of excessive biasing.

(Remarks on code availability)

Reviewer #3

(Remarks to the Author)
The authors have done a commendable job to address all the points raised by the reviewers with new experiments, detailed explanations and significant reorganization of their manuscript. I deeply appreciate the efforts that the authors made and the seriousness with which they have addressed most of the points. The authors’ responses and revisions in the manuscript have addressed the most important concerns adequately, and I would be happy to recommend the manuscript for

publication if the authors could take a look at a few remaining points of unclarity.

- Regarding FCS experiments and the authors' response to Point 1. I appreciate the authors' efforts to perform these FCS experiments. However, interpreting FCS data can be complicated, and small fractions of larger clusters are easily overlooked because of the small contribution to the autocorrelation function. If the authors choose to present these data, they should at least show the exponential fit and residuals. From the residuals it is more straightforward to assess the potential presence or absence of larger assemblies. I may be wrong, but it seems that there is a slight "long tail" in the G-1 data starting around 10^0 ms, which could indicate a small fraction of larger clusters. Moreover, of the optimized interfacial peptide sequences, the peptide designs for LAF-1 and Ddx4 condensates seem the most prone to self-assembly in the absence of condensates, but these were not tested by FCS. Related to this point, the authors may want to nuance their statement about "This observation confirms that homotypic peptide interactions are minimized, as no larger assemblies are produced by the peptide alone"

- Regarding the new Figure S13, and the conclusion that interfacial localization does not change over time: this figure should be accompanied by an analysis of the (unadjusted) intensity profiles across the condensate droplets. It is difficult to assess the change in localization from only the image where brightness and contrast settings may differ between experiments. From these images it appears that the peptide actually does leave the interface slowly to dissolve in the surrounding dilute phase, but I may be wrong.

- Regarding the new Figure S15, and the authors' response to Point 3.9. I do not agree with the authors' conclusion, and also find this figure confusing. "Assuming that the total dense phase volume remains approximately constant (a reasonable assumption given the 10:1 protein-to-peptide molar ratio), this results in a higher abundance of smaller (nano)condensates." If microscopy shows that the average size of micron-sized condensates becomes smaller and the total dense phase volume remains constant, this does not say anything about the abundance of nanocondensates. The total number of (smaller) micron-sized condensates may simply be larger, and the number and size of the nanocondensates remains the same. Moreover, Figure S15 does not show that there is a higher abundance of nanocondensates: the distributions are normalized, and only show an increase in the average size of the nanocondensates with addition of peptides. Why is that expected? Since there is no information about the relative amounts of nanocondensates and micron-sized condensates, it cannot be concluded that "indicating a global shift in the size distribution toward smaller values (Figure S15)".

(Remarks on code availability)

Version 2:

Reviewer comments:

Reviewer #3

(Remarks to the Author)
I would like to thank the authors for clarifying the remaining points, and revising their manuscript accordingly. The fact that the average size of the nanometer-sized clusters of hnRNPA1 becomes larger when the interface-localizing peptide is added (Figure S15) remains a surprising finding that probably warrants further investigation, but I appreciate that this is beyond the scope of the current manuscript and agree that it does not affect the revised conclusions. I am happy to recommend the manuscript for publication.

(Remarks on code availability)

Open Access This Peer Review File is licensed under a Creative Commons Attribution 4.0 International License, which permits use, sharing, adaptation, distribution and reproduction in any medium or format, as long as you give appropriate credit to the original author(s) and the source, provide a link to the Creative Commons license, and indicate if changes were made.
In cases where reviewers are anonymous, credit should be given to 'Anonymous Referee' and the source.
The images or other third party material in this Peer Review File are included in the article's Creative Commons license, unless indicated otherwise in a credit line to the material. If material is not included in the article's Creative Commons license and your intended use is not permitted by statutory regulation or exceeds the permitted use, you will need to obtain permission directly from the copyright holder.
To view a copy of this license, visit https://creativecommons.org/licenses/by/4.0/

Revisions - Nature Communications

De novo design of peptides localizing at the interface of biomolecular condensates

Reviewer #1 (Remarks to the Author)

Schneider et al. report on an approach to de novo design peptide sequences that seek the interface of biomolecular condensates using a computational loop consisting of coarse-grained simulations, machine learning, mixed-integer linear programming followed by in vitro experimental validation. Interfaces of water-rich, charged and dynamic liquid-liquid phase separating proteins are a challenging target with previous work focusing on identifying proteins that are capable of partitioning to the phase boundary. The ability to select for short-, synthesizable- peptides from a vast design space, is a potential key in understanding these surfaces in depth, as well as controlling the properties and function of the underlying biomolecular condensates. Towards this goal, this manuscript presents an approach rooted in using two distinct classes of CG-MD simulations to identify peptides that (i) have strong tendencies for interfacial localization using slab simulations with adaptive biasing force and (ii) those that demonstrate weak homotypic interactions to disfavor aggregation using a simulation of peptide dimers. Peptide sequences are then subjected to a machine learning and optimization pipeline to maximize the interface seeking propensity while minimizing the self-interactions in a loop with the molecular simulations. Predicted sequences are identified to be amphiphilic in nature, with aromatic rich ends preferring the interface, and charged domains pointing into the bulk. Using fluorescently labelled peptides identified using this approach, the authors demonstrate strong preferential interface seeking behavior of these molecules, with the ability to influence the size/charge of the biomolecular condensates. Although this work presents an interesting approach to design peptides with the ability to seek the biomolecular condensate interface, several conceptual questions arise which should be addressed prior to being considered for publication –
Response: We appreciate the reviewer's evaluation of our work and the thoughtful comments, which we have addressed in detail below.

1) The introduction prefaces the manuscript by presenting the idea that water- and ion-rich condensates are challenging to target using traditional surfactant molecules unlike, for example, oil-in-water emulsions. The peptides identified in this work however appear to have strong surfactant-like tendencies with amphiphilic peptide designs. Could the authors discuss why they believe that the peptide designs are distinct from surfactants that seek interfaces?
Response:
We thank the reviewer for raising this important point. Indeed, we refer to our peptide designs as surfactant-like because they share some features of traditional surfactants, including their amphiphilic, or also called condensate-amphiphilic (https://doi.org/10.1038/s41557-025-01800-4), architecture. Similar to classical surfactants, different regions of the molecule exhibit distinct preferences for different phases, resulting in preferential localization at the interface. However, there are key distinctions relative to

classical surfactant molecules (https://doi.org/10.1002/9781118228920, http://dx.doi.org/10.1039/B208499F):

1. Chemical structure and underlying molecular interactions: In classical surfactant molecules, a hydrophilic head group is typically connected to an alkyl tail, and the hydrophobic effect is the main driving force behind the exclusion of the tail from the aqueous phase. In case of the peptide designs, the tail in the condensate (excluded from the aqueous phase) is mainly composed of aromatic residues (and arginines), which are able to engage via pi-pi and cation-pi interactions with the condensate.

2. Relative size of blocks: For traditional surfactants, the hydrophobic tail is typically much larger than the hydrophilic part. This is not the case for our peptide designs, where optimization revealed that it is often more effective to dedicate a larger fraction of the sequence to residues that are preferentially excluded from the dense phase. In the hnRNPA1-LCD and LAF-1-RGG designs, this results in high positive net charges, exceeding those of classical surfactant molecules.

3. Self-association behavior: Classical surfactants are known to self-associate and form micellar structures, which can significantly influence their behavior at higher concentrations. In this study, we are maximizing interfacial localization while simultaneously minimizing homotypic interactions. This approach results in high net charges of the designs, leading to reduced self-association in silico. To experimentally confirm this, we measured the diffusion coefficient and hydrodynamic radius of the peptide for the hnRNPA1-LCD target in the absence of the protein, as a representative example. Fluorescence correlation spectroscopy (FCS) yielded a diffusion coefficient of 152 ± 20 um2/s and hydrodynamic radius of 1.41 ± 0.19 nm (mean ± SD, n = 3). The theoretical radius of gyration of a monomer, assuming a C-α separation distance of 0.381 nm, is estimated to be between 0.9 nm (ideal chain) and 3.3 nm (rod) (https://doi.org/10.1093/oso/9780198520597.003.0001). The fact that the experimental value falls within this monomeric range supports that the peptide does not form micelles under experimental conditions.

We performed these new fluorescence correlation spectroscopy (FCS) experiments to demonstrate the absence of self-association of the designed peptide. The new data are presented in new Figure S16 and discussed in the following added text, together with the additional considerations described above.

Materials and Methods - Fluorescence correlation spectroscopy (line 616)

"Fluorescence correlation spectroscopy (*FCS) experiments were performed using an inverted confocal fluorescence microscope (Leica SP8 STED) equipped with an HC PL APO CS2 63 × 1.2 NA water immersion objective with and a hybrid detector for single molecule detection (HyD SMD). Data acquisition and analysis were performed using Leica Application Suite X software (version 1.0). For the analysis of the self-association behaviour of peptide 2 targeting the interface of hnRNPA1-LCD condensates, 500 nM Cy5-labeled peptide was incubated with*

500 nM unlabeled peptide, reaching a final peptide concentration of 1 uM in 20 mM Tris, 150 mM NaCl, pH 7.5 (n=3). Samples were excited with a 640 nm laser (from a White Light Laser at 80 MHz repetition frequency), and the fluorescence emission was collected at a wavelength range of 660-700 nm. The confocal volume was calibrated using 20 nM Alexa 647 NHS Ester (Diffusion coefficient D = 330 µm²/s) and the autocorrelation curves were fitted based on a model assuming a single diffusing component."

Results and Discussion - Peptides targeting the interface of hnRNPA1-LCD condensates (line 286)

"In the absence of the protein, however, these clusters did not form. Fluorescence correlation spectroscopy (FCS) measured a hydrodynamic radius for the peptide within the monomeric range (Figure S16). This observation confirms that homotypic peptide interactions are minimized, as no larger assemblies are produced by the peptide alone."

Conclusions (line 436)

"While exhibiting surfactant-like architecture, our designed peptides also have key differences from classical surfactant molecules.[ref1,ref2] Instead of relying on alkyl tails to drive exclusion from the aqueous phase through hydrophobic interactions, our peptide designs primarily depend on cation–pi and pi–pi interactions involving aromatic residues. Moreover, unlike classical surfactants, the optimization allocates a significant fraction, if not the majority, of the peptide molecule to residues that preferentially localize in the dilute aqueous phase, thereby preventing complete recruitment into the dense phase."

New Figure S16

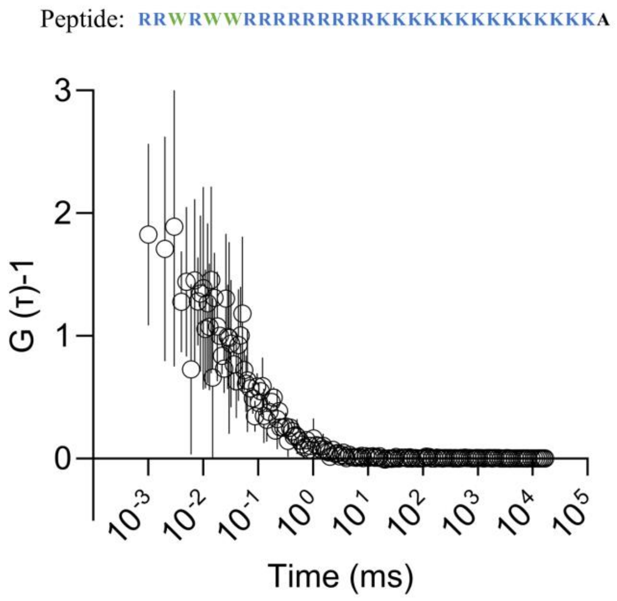

Figure S16: *FCS autocorrelation function of the peptide 2 alone (1 uM, 50% labeled) in 20 mM Tris (pH 7.5) and 150 mM NaCl. The fitted diffusion coefficient was 152 ± 20 um2/s, corresponding to a hydrodynamic radius of 1.41 ± 0.19 nm calculated using the Stokes–Einstein equation (mean ± SD, n = 3). The theoretical radius of gyration of a monomer, assuming a C-alpha separation distance of 0.381 nm, is estimated to be between 0.9 nm (ideal chain: Rg = sqrt{N} b/ sqrt{6}) and 3.3 nm (rod: Rg = N b/ sqrt{12}).[ref]*

2) Following up on the previous point, it would be prudent to discuss the enrichment of tryptophans at the C-terminal ends of the designed peptides vs. other aromatics such as phenylalanine, tyrosine or histidine in all three condensate systems evaluated in this manuscript. It has been previously reported that in case of transmembrane proteins, tryptophan residues are preferentially enriched near bilayer interfaces (Commun Chem 6, 154 (2023). https://doi.org/10.1038/s42004-023-00953-y). Furthermore, recent work on short peptides containing lysine and tryptophan residues have been shown to themselves undergo liquid-liquid phase separation and have strong tendencies to stabilize protein interfaces (Nat. Mater. (2025). https://doi.org/10.1038/s41563-025-02300-z). Do the authors propose a similar special role for tryptophan in stabilizing condensate interfaces that is selected for by the computational pipeline?

Response:

It is indeed an important observation that tryptophan is preferentially selected during peptide sequence optimization compared to other aromatic residues. Regarding the molecular mechanisms underlying its incorporation, we consider that there are common elements shared with the two studies highlighted by the reviewer, as well as key differences.

When constructing the contact map (Figure 3B, Figure S11), we found that the interaction hotspots between the designed peptides and the condensate-forming proteins mainly correspond to aromatic segments within the protein sequences, highlighting the dominant role of aromatic interactions in driving peptide-protein interactions. These strong pi-pi interactions, which are implicitly represented in the Mpipi force field (https://doi.org/10.1038/s43588-021-00155-3), are strongest when tryptophan is involved, compared to other aromatic residues. This trend is also captured in other commonly used force fields for modeling protein phase separation (https://doi.org/10.1002/pro.4094, https://doi.org/10.12688/openreseurope.14967.2). It is therefore most efficient to incorporate tryptophan residues into the parts of the sequence that need to interact with the condensate.

In the study by Dave et al., the authors also highlight the key role of pi-related contacts involving tryptophan residues, for instance in the complexation of GFP variants in solution, which drives recruitment into the dense phase, consistent with the mechanism described above. Regarding localization at bilayer interfaces, Lander et al. demonstrated, through studies of AucA-sulfate and glycerol 3-phosphate interactions (both structurally similar to the head groups of membrane phospholipids), that hydrogen bonding between tryptophan and these ions plays a key role in stabilizing binding and thus likely drives enrichment near bilayer interfaces. This mechanism is distinct from the pi-based interactions mentioned above and can be attributed to the unique heteroaromatic nature of tryptophan. Based on the considerations above, we conclude that in our case tryptophan is incorporated primarily due to the strength of its pi-based interactions rather than its capacity for hydrogen bonding. To clarify this point, we have added the following text to the manuscript.

Results and Discussion - Peptides targeting the interface of hnRNPA1-LCD condensates (line 245)

"Tryptophan is likely favoured by the algorithm over other aromatic residues because its interactions with both aromatics and arginine tend to be stronger, a feature that is reflected in the Mpipi force field.[ref]"

3) The Mpipi forcefield employs an implicit solvation model to introduce the effects of water. The lack of explicit water may potentially lead to generation of a more distinct interface in the molecular simulations between the condensate protein rich phase and poor phase leading to a strong free energy bias in the adaptive biasing force simulations. Would a more diffuse interface enabled by having explicit solvent influence the peptide sequences identified computationally?

Response:

We acknowledge that the accuracy of the predicted behavior is constrained by the accuracy of the force field used. Ideally, the design process should be performed using an all-atom representation, which would capture explicit solvation and generally a much more complex conformational landscape. However, this is computationally intractable, as a large number of peptides must be simulated for an efficient design. The current computational cost per peptide, dominated by the interface partitioning simulation, is approximately 16 CPU days (see Supplementary Software file). For the hnRNPA1-LCD target, ~700 sequences were simulated, resulting in a total computational cost of ~30 CPU years. Even less coarse-grained models such as MARTINI are computationally too demanding for use in our design pipeline as is, not only because they contain more particles per system but also because the more rugged free energy landscape leads to slower system dynamics and therefore requires longer simulations.

To address this reviewer point regarding explicit solvation, we performed simulations of the three interface-partitioning peptides that were experimentally validated, using the Martini3-IDP force field (https://doi.org/10.1038/s41467-025-58199-2), to investigate if they also exhibit a preference for the condensate interface in this less coarse model with explicit solvent. We assessed convergence of the unbiased simulations by monitoring the average peptide density profiles across 16 replicas for all three systems. The total computational cost required for convergence of these simulations was ~40 CPU years for the three peptides only. The results showed a clear preference for the interfacial region over the dense and dilute phase in two out of the three cases. We presented these findings into the manuscript by adding the following text and Figure S23.

Results and Discussion - Designing peptides targeting other protein condensates (line 378)

“It is important to note that the optimality of the design depends on the accuracy of the Mpipi force field used. This may introduce potential bias during the optimization procedure, for example due to the lack of secondary structure elements, explicit solvation, or the representation of the dominant role of pi-based interactions in Mpipi. To evaluate this, we conducted an additional set of simulations using the CALVADOS 2[ref] and the more detailed Martini3-IDP[ref] force fields to examine the interface partitioning of the three experimentally validated peptides. This approach provided an orthogonal perspective on our designs. CALVADOS 2 simulations were performed at a slightly lower temperature (as detailed in Materials and Methods), yielding comparable slab densities to those obtained with Mpipi. These simulations also indicated a preference for the peptides to localize in the interfacial region rather than in either the dense or dilute phases across all three cases. The interaction patterns observed in the contact maps were similar to those from Mpipi, exhibiting surfactant-like behavior (Figures S21-22).

In the case of Martini3-IDP, interfacial localization was clearly confirmed for two peptides. The contact maps revealed still evident surfactant-like interaction profiles that share common

features with the Mpipi results, although weaker and with some differences compared to Mpipi (Figures S23-24).

In conclusion, although as expected the optimal designs are likely to vary to some extent across different force fields, a certain degree of transferability exists. Continued advancements in force field development will further enhance the reliability of peptide designs generated by our computational pipeline."

Conclusions (line 449)

"It should be also noted that the optimality of the design depends on the accuracy of the force field used, although we observed a reasonable agreement across different force fields for our selected peptides."

Materials and Methods (line 515)

"Martini simulations

Slab coexistence simulations were carried out in GROMACS 2021.4[ref] using the Martini3-IDP force field[ref], in the NPT ensemble applying a velocity-rescaling thermostat[ref] (300 K, 1 ps) and a Parrinello-Rahman barostat[ref] (1 bar, 12 ps). Initial peptide and IDP geometries and topology files were generated using Polyply[ref], followed by energy minimization and a 100 ps simulation leading to compact conformations. The condensate slab was generated by random insertion of a total of 16 protein copies into the center of the periodic box (same box dimensions as in LAMMPS simulations), and two peptides were inserted into the dilute phase. Coarse-grained water and ions corresponding to an ionic strength of 150 mM were added, followed by energy minimization and short equilibration, yielding the initial configuration for the production runs. Each system was simulated in 16 independent replicas of 60 us; the final 20 us were used for analysis. Convergence was assessed based on the mean center-of-mass distance between the slab and the peptide, as well as the peptide concentration in the dense phase. Coordinates were saved every 10 ns, and the contact map was constructed identically to the LAMMPS simulations, using the positions of the backbone beads."

New Figure S23

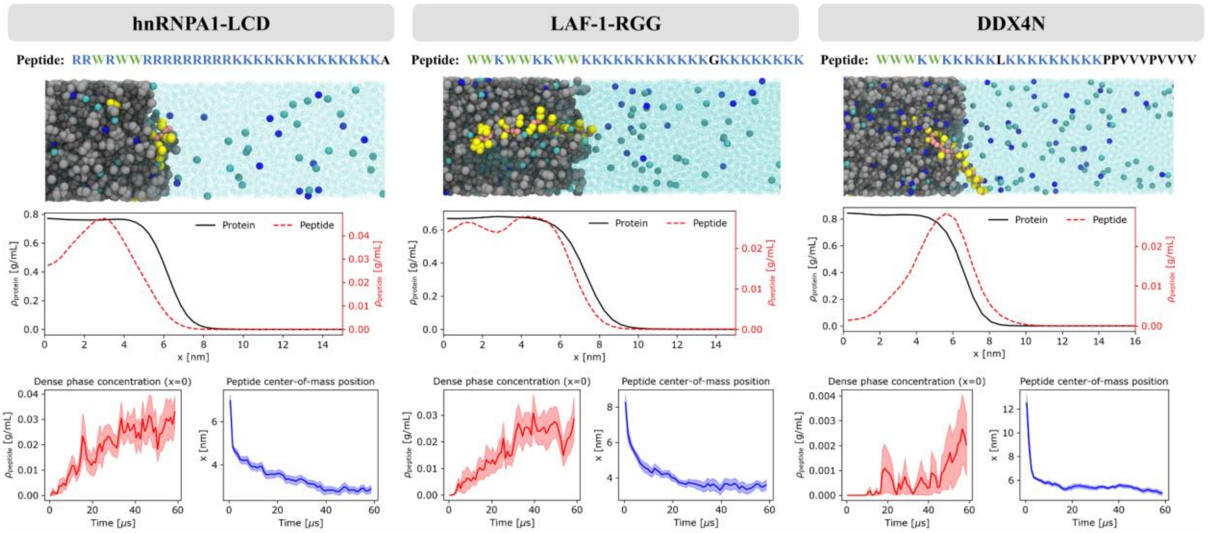

Figure S23: *Results of Martini3-IDP slab coexistence simulations. Convergence was assessed from the peptide dense-phase concentration and center-of-mass position. The*

final 20 us were used to construct density profiles and contact maps. Uncertainties correspond to standard errors from 16 independent simulations.

4) The pipeline emphasizes the use of tools such as WALTZ, AGGRESCAN and TANGO to identify and remove sequences with the potential of undergoing aggregation. Several of these tools are biased towards beta-sheet rich sequences derived from or having analogy to amyloid proteins. Could this be a potential drawback as some amyloid like sequences can be amphiphilic and interface seeking but would likely be eliminated by the computational workflow?

Response:

We agree with the reviewer that incorporating these predictors reduces the design space that can be explored in our pipeline. However, we retain these filters for beta-sheet rich / amyloidogenic sequences to prevent the potential design of experimentally problematic, aggregating peptides. Because secondary-structure formation cannot be modeled in the coarse-grained simulations used to quantify homotypic interactions, including tools that recognize beta-sheet rich sequences provides an orthogonal safeguard against such aggregation-prone peptides. Given that the overall design space is very large (~20^{30} possible sequences), incorporating these filters still allows for substantial diversity and the identification of promising candidates. We acknowledge that the design space is reduced by adding the following statement to the main text:

Results and Discussion - Overall workflow (line 110)

"While the incorporation of these filters into the computational pipeline excludes a subset of potential peptides, the large design space still allows for the design of a highly diverse set of candidates."

5) The adaptive biasing force simulations generate PMF curves using a simulation of one peptide chain from the condensate proteins. Interface seeking peptides would also likely self-interact or be in close proximity at the interface and with charged (lysine rich tails) have high repulsion and therefore lower interface loading. It would therefore be useful in future work, to also account for lateral interactions between interface seeking peptides and design in some favorable self-interactions.

Response:

We thank the reviewer for this comment. We have added a statement to the conclusion:

Conclusions (line 445)

"It is important to note that the simulation setup used to quantify interfacial partitioning assesses the behavior of individual peptides. Therefore, the pipeline optimizes interfacial localization at low concentrations. A natural extension of this computational pipeline would involve conducting simulations at varying concentrations, accounting for lateral peptide interactions within the interfacial region."

6) The amphiphilic peptide sequences designed in this work, although having been through a pipeline to eliminate self-aggregation, are likely to undergo self-assembly. The authors also report the presence of nanosized clusters using dynamic light scattering analysis in Figure S10. Is it therefore a strictly necessary condition to minimize and/or eliminate peptide

self-aggregation as a criterion for designing biomolecular condensate interface seeking peptides?

Response:

The nanosized clusters observed in solutions containing hnRNPA1-LCD were only detected in the presence of the protein. To address this point, we performed fluorescence correlation spectroscopy (FCS) measurements of the peptide alone in solution at the same concentration as in Figure S15 (old Figure S10), which yielded a hydrodynamic radius consistent with a monomer, indicating the absence of larger assemblies. We therefore conclude that minimization of homotypic interactions was successful in this case. Homotypic interactions are minimized in our design pipeline to prevent potential self-association at higher concentrations, where it could become detrimental, but should not be a strict requirement in the low-concentration limit. Because the entire Pareto front is constructed at every iteration, the pipeline also identifies the peptide that is optimal in the interface-partitioning objective only.

The new experimental data and corresponding text modifications are described in the response to **reviewer point 1.1.**

7) Could the authors comment on whether any potential reorganization of the condensate forming protein chains is observed upon the interfacial binding of the peptides? The contact map in Figure 3B is interesting and highlights the importance of cation-pi type contacts between the peptide and hnRNPA1-LCD protein condensate. However, it would also be insightful to comment on the distribution of condensate residues at the interface in the absence and presence of the peptide, particularly in the local proximity to the peptide contacts.

Response:

To address this reviewer point, we performed new Mpipi simulations with varying peptide concentrations for all three experimentally validated cases and analyzed the distribution of different residue types within the protein dense phase. In the case of hnRNPA1-LCD, the presence of the peptide leads to a slight enrichment of negatively charged and aromatic residues in the interfacial region relative to the dense phase. This observation is consistent with the contact map, which shows that the peptide preferentially interacts with these residue types. Positively charged residues appear slightly depleted, most likely due to electrostatic repulsion with the positively charged peptide. The distribution of aliphatic residues remains largely unaffected. For LAF-1-RGG, the outer interface layer primarily consists of aliphatic residues (see new Figure S9). Upon peptide addition, this region becomes more enriched in positive, negative, and aromatic residues. Interestingly, for DDX4N, no significant reorganization is observed upon peptide addition. We therefore conclude that the presence of interface-localizing peptides can, but does not necessarily, alter the distribution of residue types at the condensate interface.

New Figure S9

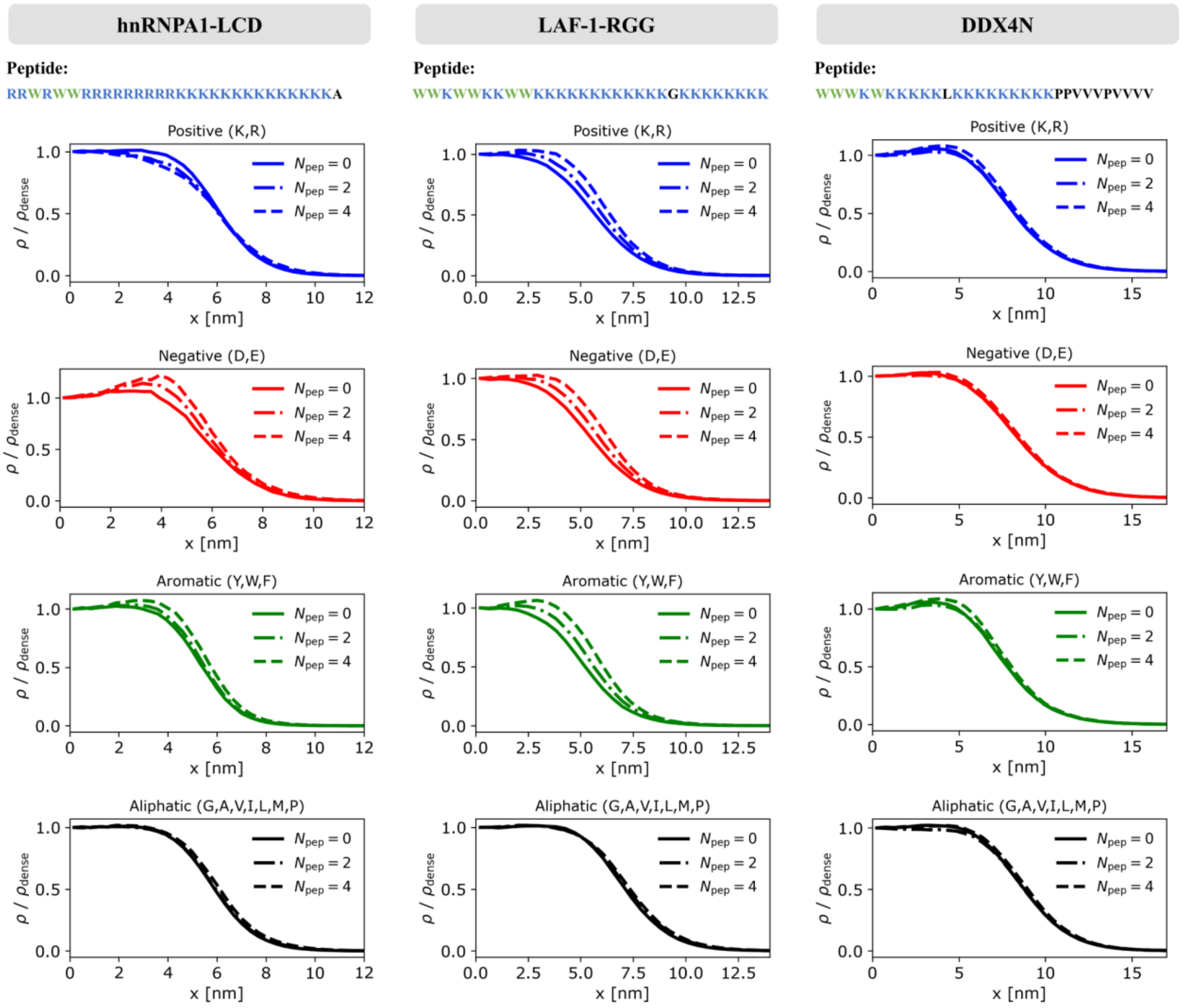

Figure S9: *Distribution of different protein residue types within the dense phase as a function of the distance from the slab center of mass for varying numbers of peptides in the simulation box.*

Results and Discussion - Peptides targeting the interface of hnRNPA1-LCD condensates (line 250)

"We performed additional simulations to investigate how interfacial composition varies with peptide concentration (Materials and Methods). Increasing peptide concentration enriched aromatic and negatively charged protein residues at the interface, consistent with the contact map and suggesting that the peptide may not only localize but also induce structural rearrangements in the interfacial region (Figure S8-9)."

Materials and Methods - Contact maps and interfacial organization (line 508)

"For analyzing interfacial composition, simulations were repeated with different numbers of peptide copies using 5 independent replicas per condition, and the distribution of residues was analyzed using the MDAnalysis package[ref]. At high peptide concentrations, occasional rupture of the protein slab occurred; the corresponding frames were identified using wavelet transformation–based detection of multiple density peaks[ref] and excluded from the analysis."

Reviewer #2 (Remarks to the Author)

In the manuscript "De novo design of peptides localizing at the interface of biomolecular condensates" submitted by T. Schneider et al., a computational workflow is presented with the goal of predicting new peptides at the interface with biomolecular condensates. The work is incredibly well written, making it very readable for a broad audience, particularly those who are interested in interfacial polymer science and peptide/protein design in the modern era of machine learning and artificial intelligence. The work is complemented with experimental validation of a few of the discovered peptides. One of the major claims stated in the abstract and introduction of the manuscript, however, may not be satisfied. The authors state on Line 9, Page1 “The molecular grammar driving the preferential localization of molecules at condensate interfaces remains largely unknown.” It is very possible that the use of the coarse grained force field Mpipi biased the results towards Arginine/Tryptophan rich results as the original paper describing Mpipi states, “ The model is parameterised from both atomistic simulations and bioinformatics data and accounts for the dominant role of π–π and hybrid cation-π/π–π interactions and the much stronger attractive contacts established by arginines than lysines.” If one were to use a more popular coarse grained force field like MARTINI, what would the results look like? Regardless of the bias towards these interactions, the authors have developed a workflow that identifies several peptides with predicted interfacial localization. I would recommend this work to Nature Communications if the authors addressed the issue of molecular biasing with the Mpipi coarse grained force field and refine the identified “molecular grammar” addressing some of the concerns below.
Response: We appreciate the reviewer’s evaluation of our work and the thoughtful comments, which we have addressed in detail below.

1. The workflow (pp.5-7) is incredibly well described for a broad audience. The definition of the two important parameters utilized (probability of localization at the interface, p(int), and homotypic interactions, B2) are clearly explained. And defining Pareto solutions as “a set of globally optimal, non-dominated sequences that cannot improve one objective function without compromising the other one” is clear and succinct. These clear definitions demonstrate the authors’ excellent communication throughout the presented work.
Response: We thank the reviewer for this positive comment.

2. The authors explain Mpipi as their force field choice on p. 7 line 132. “ a one-bead per residue mode, due to its physics-based parametrization strategy and ability to explain experimental data of disordered proteins.” This is well justified for the topic, but this reviewer would be curious to hear the thoughts of the authors regarding the possibility that Mpipi may have skewed the peptide design towards the favorable R/W interactions. If one were to plot a histogram of the amino acid content within the peptides predicted, the bias towards R/W/K would be clear.
Response:
We acknowledge that the performance of the design pipeline is constrained by the accuracy of the force field, and that any inaccuracies may be exploited by the optimization pipeline, for example overly strong R/W interactions as noted by the reviewer. When considering other popular coarse-grained force fields for disordered proteins, such as CALVADOS 2 (https://doi.org/10.12688/openreseurope.14967.2) or HPS-Urry (https://doi.org/10.1002/pro.4094) tough, we find that there are several common elements, that

play a key role in our peptide designs. In both force fields, W/X interactions are stronger compared to other aromatics, and W/R interactions are stronger than W/K interactions, as in Mpipi. Also, interactions involving V are generally the weakest in CALVADOS 2 among all neutral amino acids, which is also key for the DDX4N designs.

To address the reviewers' comments, we ran new simulations for the three peptide designs selected for experimental validation using the Martini3-IDP (see answer to reviewer point 1.3) as well as the CALVADOS 2 force field to assess the transferability of our results. Simulations with the CALVADOS 2 force field at 260K, leading to slab densities comparable to those obtained with the Mpipi force field, resulted in preferential interfacial accumulation of all three peptides. In the case of Martini3-IDP, interfacial localization was confirmed in two out of three cases. We also created contact maps with comparable interaction patterns across force fields (see answer to reviewer point 2.6). Altogether, we acknowledge that the optimal designs are likely to differ to some extent when using different force fields. However, the interfacial localization behavior and comparable contact maps across three different force-fields strongly support our design.

To address this reviewer point, as well as more generally to discuss the dependence of our findings on the chosen force field, we made the following modifications to the main text and supporting information. We also added the analysis requested by the reviewer in this point, where we plot peptide composition as a function of iteration during the optimization. It shows that initially compositions are more diverse, and that W and K content plateaus at a high level for the hnRNPA1-LCD / LAF-1-RGG case, and also R for hnRNPA1-LCD. The average compositions in the case of DDX4N appear to be more heterogeneous, but the high W content in the last couple of iterations is also a consistent feature.

Results and Discussion - Designing peptides targeting other protein condensates (line 378)

"It is important to note that the optimality of the design depends on the accuracy of the Mpipi force field used. This may introduce potential bias during the optimization procedure, for example due to the lack of secondary structure elements, explicit solvation, or the representation of the dominant role of pi-based interactions in Mpipi. To evaluate this, we conducted an additional set of simulations using the CALVADOS 2[ref] and the more detailed Martini3-IDP[ref] force fields to examine the interface partitioning of the three experimentally validated peptides…." (see answer to reviewer point 1.3)

Conclusions (line 449)

"It should be also noted that the optimality of the design depends on the accuracy of the force field used, although we observed a reasonable agreement across different force fields for our selected peptides."

New Figure S21

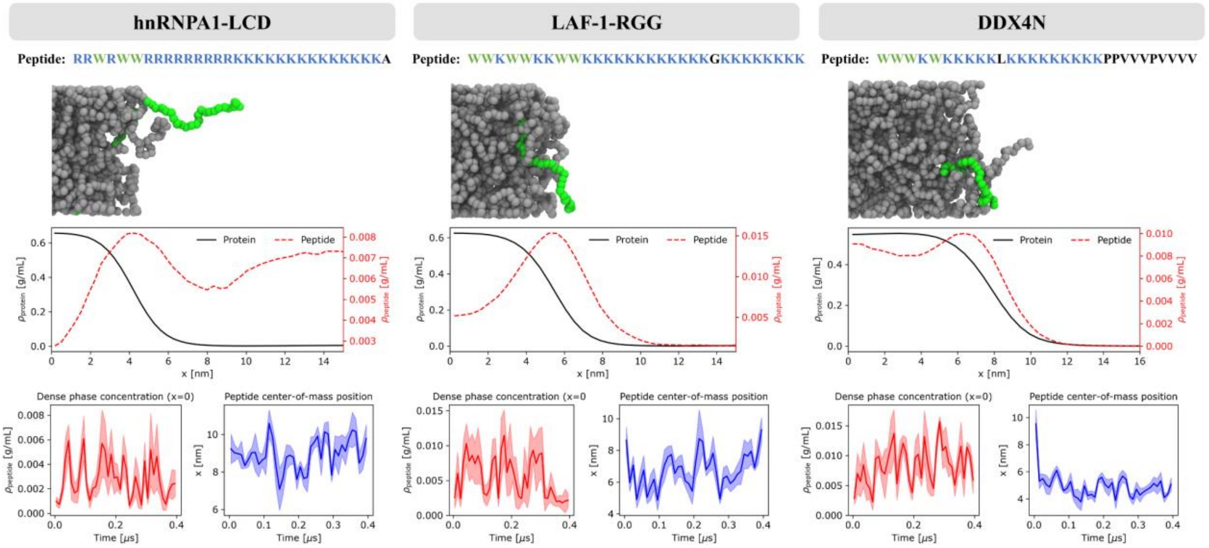

Figure S21: Results of CALVADOS 2 slab coexistence simulations. Convergence was assessed from the peptide dense-phase concentration and center-of-mass position. The final 300 ns were used to construct density profiles and contact maps. Uncertainties correspond to standard errors from 5 independent simulations.

Materials and Methods (line 531) - Calvados simulations

"Slab coexistence simulations were carried out in OpenMM 8.2.0[ref] using the CALVADOS 2 force field at an ionic strength of 150 mM[ref], applying a Langevin thermostat at 260 K with a relaxation time of 100 ps. The simulation temperature was slightly lower than in the Mpipi runs, resulting in similar slab densities. Initial topology and coordinate files were generated using the CALVADOS Python package[ref]. 16 proteins and 2 peptides were inserted into a simulation box with the same dimensions as in the LAMMPS simulations. After 100 ns of equilibration, a 300 ns production run was performed, and contact maps were constructed analogously to the other cases."

New Figure S23 (Martini simulations)

See reviewer point 1.3

Materials and Methods (line 515) - Martini simulations

See reviewer point 1.3

New Figure S6

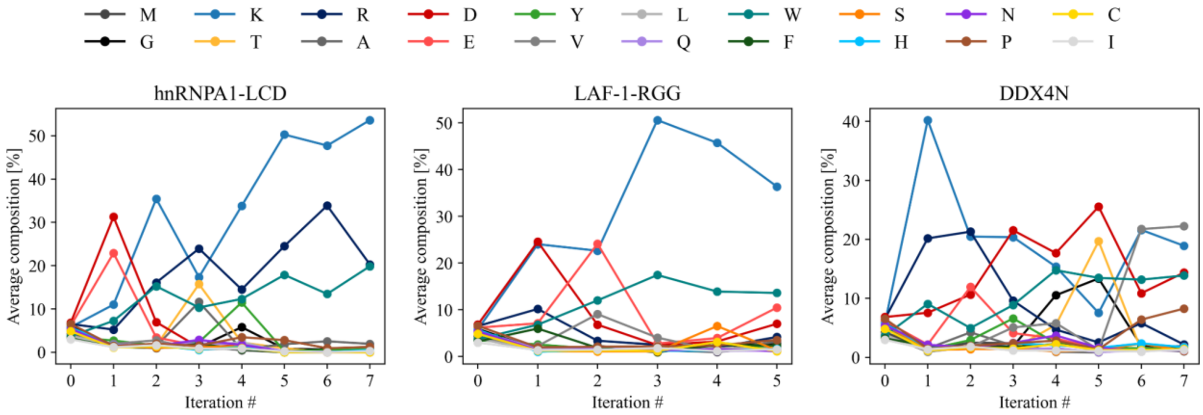

__Figure S6:__ Average peptide composition across iterations for all optimization runs.

3. Please provide the sequences (in the supporting information) of the biomolecular condensates used: hnRNPA1, LAF-1-RGG, and DDX4N. From the simulation results of the condensates, can the authors please present a color-coded model from their simulations indicating the charged, aromatic, and aliphatic amino acids. This would help the reader understand where the potential interactions might occur. For example, are the aliphatic residues buried on the interior of the condensates?

Response:

We have added the protein sequences and the color-coded snapshots requested by the reviewer. We also analyzed the distribution of positive, negative, aromatic, and aliphatic residues within the condensate for all three cases, in the presence of the corresponding peptides. The reorganization of the condensate upon peptide addition is discussed in more detail in the answer to reviewer point 1.7. Generally, the aliphatic residues do not appear to be particularly more buried within the condensate. In the case of LAF-1-RGG, the opposite appears to be the case.

New Figure S11

Protein sequences (see reviewer point 2.5)

New Figure S8

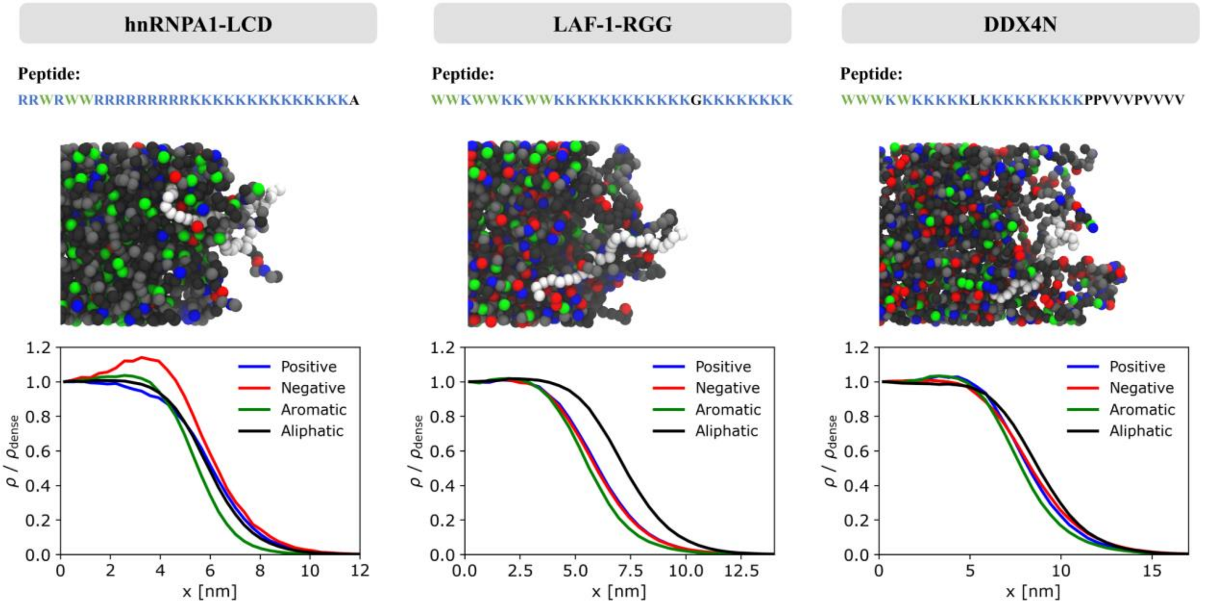

__Figure S8:__ Distribution of different protein residue types (positive: K/R, negative: D/E, aromatic: Y/W/F, aliphatic: G, A, V, I, L, M, P) within the dense phase of the simulation box containing 16 protein copies and 2 peptides. x is the distance from the slab center of mass.

Results and Discussion - Peptides targeting the interface of hnRNPA1-LCD condensates (line 250)

"We performed additional simulations to investigate how interfacial composition varies with peptide concentration (Materials and Methods). Increasing peptide concentration enriched aromatic and negatively charged protein residues at the interface, consistent with the contact map and suggesting that the peptide may not only localize but also induce structural rearrangements in the interfacial region (Figure S8-9)."

4. The production of the contact map (Figure 3B) highlights that there are favorable R/W interactions between the peptide ("Peptide 2") and hnRNPA1. These results are further supported by the experiments in Figure 4. However, when presenting the interface of peptides with LAF-1-RGG and DDX4N negatively charged peptides are suggested interfacial peptides. However, the peptides that were chosen for experiments for both LAF-1-RGG and DDX4N are incredibly similar to that investigated with hnRNPA-1, i.e. aromatic + lysine. Therefore, it seems that the authors' choice biased the "molecular grammar". The authors conclude on p. 18 that the following physicochemical traits are common for all discovered peptides: surfactant-like, aromatic rich, and matched net charge.

Response:

Regarding the reviewer's first point about possible selection bias: after applying the aggregation filters, we selected candidates from the filtered Pareto front (Figure S10). While considering reduced homotypic interactions, we prioritized keeping the primary interfacial localization objective (p_int) close to the maximum value observed. As noted by the reviewer, negatively charged peptides were also Pareto-optimal for LAF-1-RGG and DDX4N, but these showed lower p_int values and were therefore not selected for experimental validation. We conclude that the sequence similarity of the tested designs results from the computational pipeline combined with prioritization of the p_int objective rather than a bias in selecting similar sequences. We have clarified this rationale for selecting Pareto-optimal sequences in the main text:

Results and Discussion - Designing peptides targeting other protein condensates (line 340)

"Consistent with the hnRNPA1-LCD case, we selected a Pareto-optimal peptide with a value of the primary p_{int} objective close to the maximum observed, leading to an efficient tradeoff with low homotypic interactions."

The second point of the reviewer regarding the physicochemical traits is answered point by point below.

a. Surfactant-like: This seems to be consistent with the findings.
b. Aromatic Rich: this is potentially biased by the use of Mpipi.

We refer to the answer to reviewer point 2.2 where we address potential force field biases.

c. Matched net charge: not supported by experimental work.
i. No experimental evidence was presented indicating that negatively charged sequences do or do not yield interface partitioning.

We acknowledge that we did not experimentally validate negatively charged designs, as they performed worse in the p_{int} objective compared with positively charged alternatives. In response to the reviewer's comments regarding the term "molecular grammar," we have replaced it with "design principles" in the abstract. This change better reflects that our work focuses not on experimentally characterizing a wide range of distinct peptide sequences, but on designing optimal peptides for a given condensate target.

Abstract (line 27)
"Overall, our pipeline represents a promising strategy for the rational design of interface-localizing peptides and the identification of corresponding design principles."

Furthermore, we would like to highlight that we mention a matched net charge as a key feature underlying interfacial localization for hnRNPA1-LCD and LAF-1-RGG, but not for DDX4N (line 350): *"In the case of DDX4N (Figure 5D), the optimization results differed significantly from the other two cases. Most Pareto-optimal sequences did not exhibit a tail matching the condensate's net charge, as observed in the previous cases, even though such architectures were explored during the optimization process."*

ii. The valine rich segments of peptide 2 might embed in the DDX4N interior if the hydrophobic residues of the condensate are buried. The many lysine residues most likely remain outside of the condensate.

To address this reviewer point, we created contact maps of the DDX4N/peptide system for three different force fields: Mpipi, CALVADOS 2, and Martini3-IDP. Across all force fields, the surfactant-like behavior was consistent, with the aromatic head group preferentially interacting with the protein dense phase and the valine-rich tail showing reduced interaction. The lysine-rich middle section displayed intermediate levels of interaction with the protein and less than the aromatic head, consistent with the reviewer's suggestion.

Manuscript modifications and new figures related to the new contact maps are described under reviewer points 2.5 and 2.6.

5. Contact maps were not generated for these interactions: LAF-1-RGG/Pep2 and DDX4N/Pep2. They would help support some of the "molecular grammar" claims.

We agree with the reviewer that these contact maps provide valuable additional information, and we have added them to the manuscript.

New Figure S11

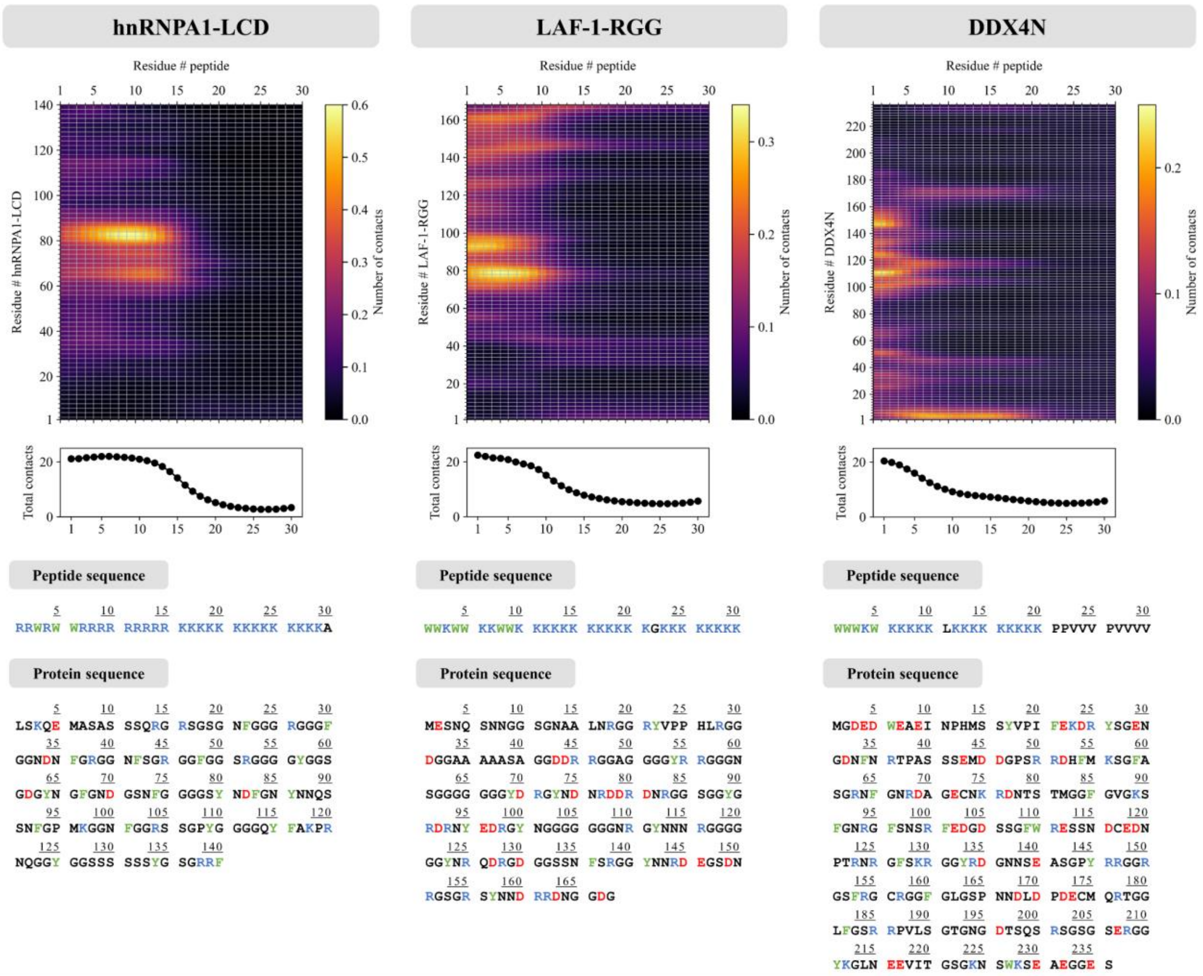

Figure S11: *Protein–peptide contact maps for the experimentally validated cases. The hnRNPA1-LCD case is also shown in Figure 3.*

Results and Discussion - Designing peptides targeting other protein condensates (line 321)

"The lysine-rich region was excluded from the condensate phase in the simulations, as visible in the contact map (Figure S11)."

Results and Discussion - Designing peptides targeting other protein condensates (line 354)

"Instead, the peptide sequences often featured valine-rich tails---which interact only weakly with most amino acids according to the Mpipi force field and are excluded from the dense phase (Figure S11)---as well as polylysine patches."

6. Contact maps generated from other force fields would strengthen the molecular grammar arguments, but if the use of Mpipi is intentional to force those strong pi-pi interactions as a dominant design feature, then that needs to be clearly stated.

In response to this reviewer point, we generated contact maps using the CALVADOS 2 and Martini3-IDP force fields. The interaction patterns obtained with CALVADOS 2 closely resemble those from Mpipi, with most interaction hotspots along the protein sequence being present in both force fields. The surfactant-like behavior is also reproduced with CALVADOS

2. In the case of Martini3-IDP, we observed some common hotspots within the contact map, but the surfactant-like behavior was weaker, especially in the hnRNPA1-LCD and LAF-1-RGG cases. We discuss these new findings at the end of the Results section, together with considerations of potential force field biases.

New Figure S22

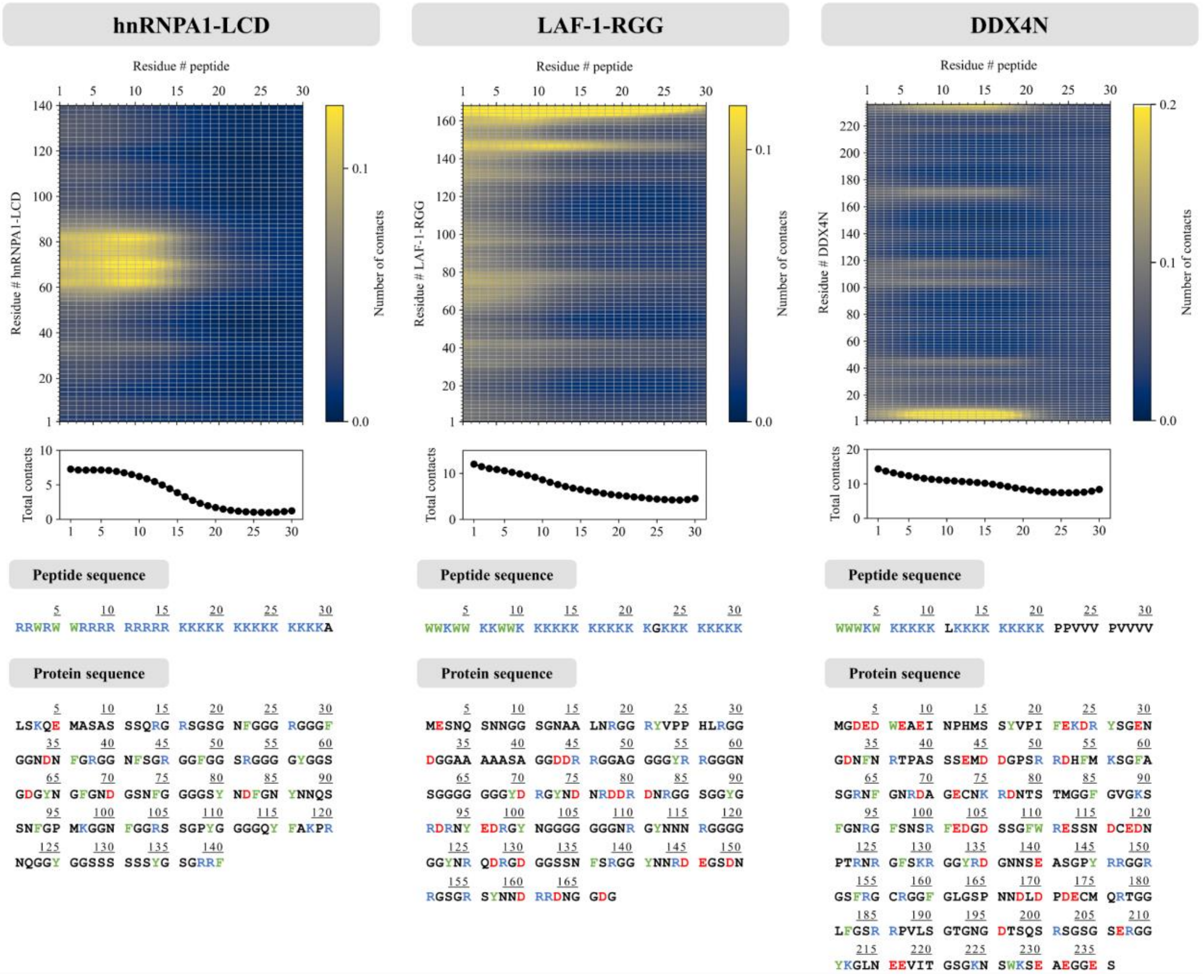

Figure S22: *Protein–peptide contact maps for the experimentally validated cases, generated using the CALVADOS 2 force field.*

New Figure S24

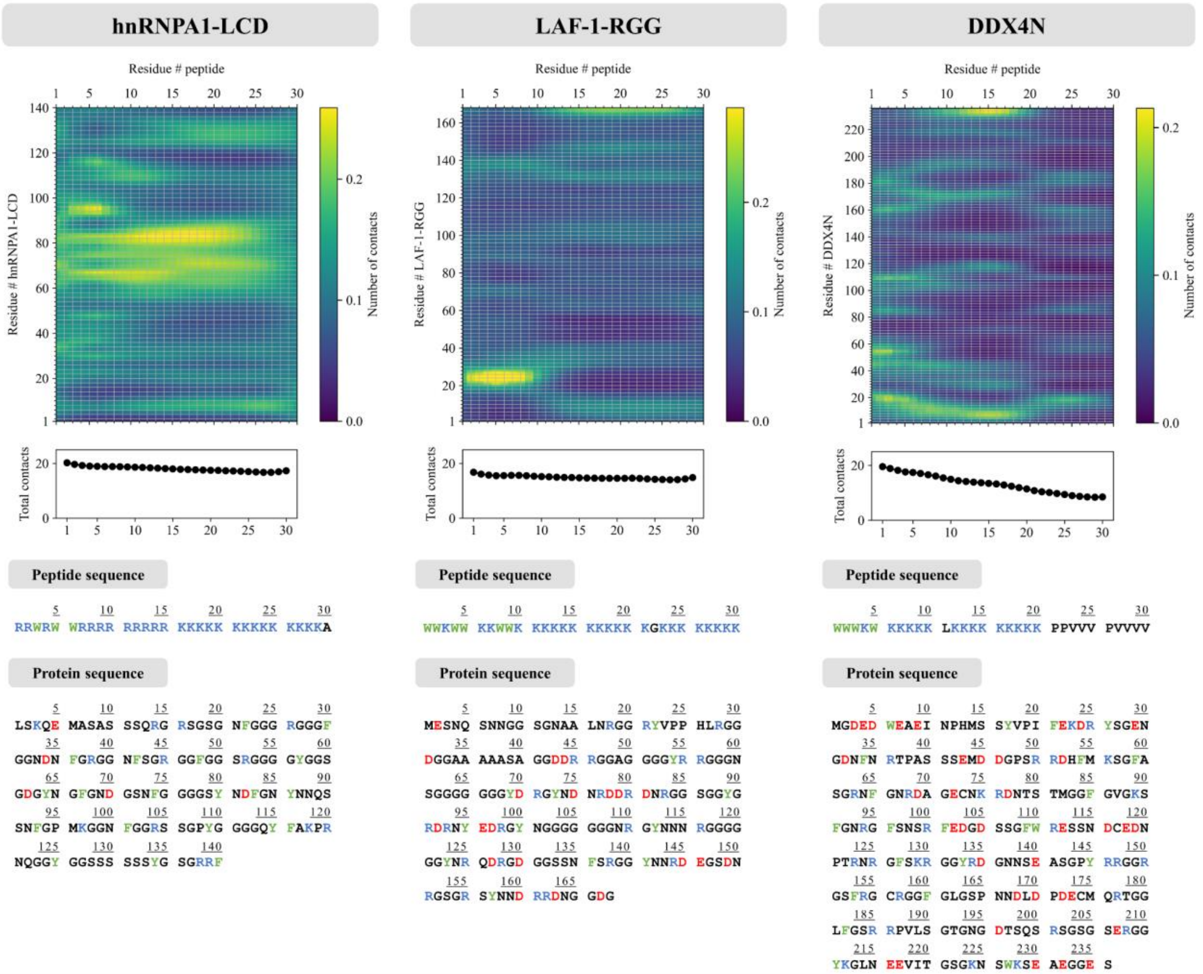

Figure S24: *Protein–peptide contact maps for the experimentally validated cases, generated using the Martini3-IDP force field.*

Results and Discussion - Designing peptides targeting other protein condensates (line 378)

Comparison to other force fields, see reviewer point 1.3

Reviewer #3 (Remarks to the Author):

The manuscript “De novo design of peptides localizing at the interface of biomolecular condensates” by Timo Schneider and co-workers presents a computational pipeline starting from coarse-grained MD simulations and an active learning loop to optimize peptide sequences for their ability to localize at the interface of biomolecular condensates. The authors use this pipeline to design interface-localizing peptides for three condensate systems, hnRNPA1, LAF1 and Ddx4. They synthesized one selected Pareto-optimal peptide sequence for each system, and showed that these selected peptides indeed localized to the interface of their respective condensate systems, and that it results in a smaller average condensate size compared to no or a random peptide.

Understanding the interfacial properties of condensates is of significant importance, given the recent examples in which condensate interfaces play an important role: amyloid

formation of client proteins and the liquid to solid transition of protein droplets and condensate interface potentials that can promote redox reactions. The manuscript provides a new approach to design peptides that specifically localize to the interface and potentially change condensate interfacial properties, which may also help to gain a better understanding of the (dynamic) nature of the condensate interface. In light of this novel approach to design potentially relevant peptides that may advance our understanding of condensates, and the current interest in condensates from a biological point of view, this manuscript is likely of interest to the community and readership of Nature Communications. However, the authors should address the following serious concerns before the work can be considered for publication.

Major:
1. The novelty of this work in relation to other recent papers on the interfacial localization of proteins or peptides on condensates should be more explicitly mentioned. In particular, the sentence "However, to our knowledge preferential interfacial localization has not been demonstrated for small molecules or peptides." (p.3) should be revised. There have been several recent manuscripts describing peptide derivatives, rationally designed polymers, and dendrimers that preferentially localize to the interface of condensates or coacervates.
- https://www.nature.com/articles/s41557-025-01800-4
- https://pubs.acs.org/doi/full/10.1021/jacs.5c03740
- https://pubs.acs.org/doi/10.1021/jacs.5c09772
- https://pubs.acs.org/doi/10.1021/acsnano.4c16908
- https://www.sciencedirect.com/science/article/pii/S0021979725020004
- https://pubs.acs.org/doi/10.1021/acs.langmuir.5c03118

Moreover, there is also an entire body of work on stabilization of such droplets by membranes assembled from small amphiphilic molecules, including fatty acids and phospholipids. These should be considered small molecules. Examples include:
- https://www.nature.com/articles/nchem.1921
- https://pubs.acs.org/doi/10.1021/jacs.0c12494
- https://advanced.onlinelibrary.wiley.com/doi/full/10.1002/advs.202412312
- https://www.biorxiv.org/content/10.1101/2021.02.19.432011v3

and many more.
Some of these works have explicitly also looked at the effects of amphiphiles on droplet size. I do not think all these papers have to be cited, but it is important to place the current work in the right literature context.

Response:

We thank the reviewer for highlighting these relevant studies. We have incorporated them in the Introduction and Conclusions. Specifically, we explicitly mention this prior work on interface-localizing polymers, peptide derivatives, as well as condensate encapsulation within lipid bilayers. We also highlight previous studies showing modulation of coalescence behavior and interfacial tension by such molecules, which is consistent with our findings.

We have also clarified the novelty of our work: the computational pipeline enables a systematic, high-throughput exploration of sequence space to design interface-partitioning peptides tailored to a given condensate target. The pipeline reduces human bias and makes it feasible to focus on short (30-residue) peptides, for which achieving interfacial localization is intrinsically more challenging than for larger proteins or polymers.

Introduction (line 54)

"For a set of natural and rationally designed proteins, interfacial localization has been confirmed[refs], and some condensates exhibit colloidal stability based on mechanisms reminiscent of Pickering emulsions.[refs] Also, condensates interfaces have been targeted by encapsulation in lipid bilayers, formed by for instance fatty acids or phospholipids.[refs] There have also been a set of engineered, potentially dendritic, polymers[refs] as well as peptides covalently fused to PEG polymers.[refs] Generally, reducing molecular size by designing short peptides while maintaining interfacial localization is challenging, because fewer interaction sites are available per molecule. In other words, the penalty in translational entropy associated with interfacial confinement becomes increasingly difficult to offset when decreasing the size of the molecule.[refs]"

Introduction (line 88)

"In contrast to previous studies that generated interface-localizing molecules often through one-shot rational design, our high-throughput, simulation-based approach augmented by active learning enables a more thorough exploration of the design space, allowing the identification of efficient short-peptide sequences tailored to specific condensate targets."

Conclusions (line 413)

"This is consistent with findings showing that interface-localizing molecules can influence both the coalescence behavior and the surface tension of biomolecular condensates.[refs]"

Conclusions (line 418)

"This architecture is similar to the amphiphilic, sometimes also referred to as condensate-amphiphilic, nature of larger proteins, polymers, and PEG-modified peptides previously shown to partition at the interface of biomolecular condensates.[refs]"

2. My main issue with the MD part of the workflow is that the one-bead per residue model is an extremely crude, oversimplified representation of the true interactions between peptides. Combined with the fact that the simulation only contains 16 protein copies, I am doubtful that this model can accurately capture the chemical nature of the different condensate systems, let alone the interface. When looking at the snapshots in Figure 3b for example, every protein is both in the bulk and at the interface, and the simulated peptide will almost always be "at the interface", simply because the system is so small. This point is salient, because the peptides that come out of this pipeline have remarkably low sequence complexity, but the authors derive "key physicochemical design parameters" from the predicted sequences. One may even ask if these sequences could not have been simply designed by hand. In particular, two questions arise here: is the system size large enough to accurately determine the relative size of the aromatic-rich block and the charge-dense block?

Response:

We acknowledge that the one-bead per residue model is a coarse representation of real molecular interactions, and that final designs may vary when other simulation models are used. In this revision, we performed additional simulations using CALVADOS 2 and Martini3-IDP for the three selected designs and generated corresponding contact maps (see answer to reviewer 1's point 1.3, and reviewer 2's points 2.2, and 2.6). The reasonable agreement between models, together with experimental validation, supports the physical relevance of our findings despite the simplicity of the representation in Mpipi.

We also want to note that even though the obtained sequences exhibit low sequence complexity, there are still very non-trivial aspects that our optimization pipeline addresses, such as the composition and residue types in the different sequence segments, as well as the relative size of these segments. We expect that these findings will guide future design of interface-localizing molecules targeting specific condensates.

To address the reviewer point of whether the system size in the simulations is sufficiently large, we repeated the adaptive biasing force simulations for a larger system size (128 proteins) and compared the results to the original simulation setup (16 proteins), for the specific case shown in the snapshot in Figure 3b. The results are highly similar, illustrating that the smaller simulation setup is justified to reduce the computational cost of the pipeline. We have added the result to the SI, and added a comment in the Methods section.

New Figure S26

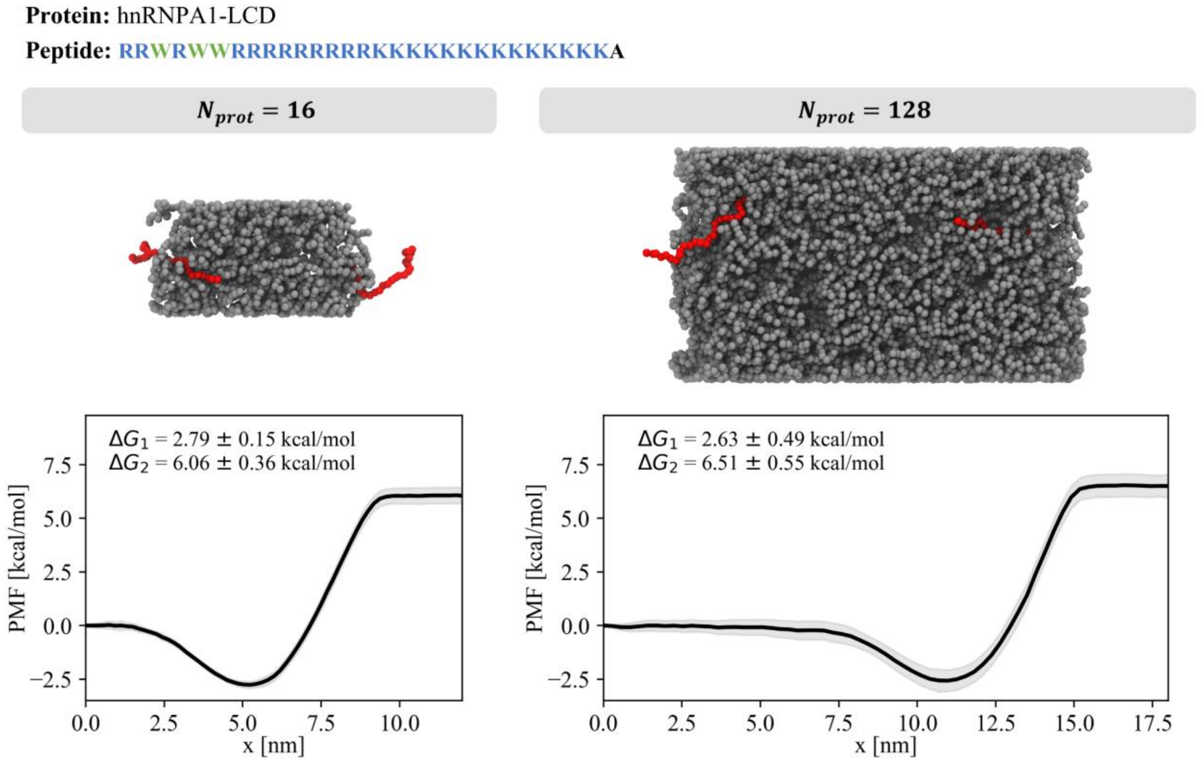

Figure S26: *Interface partitioning adaptive biasing force simulation results for two different system sizes. x is the distance from the slab center of mass, and the indicated errors correspond to standard deviations (small system: n=3, large system: n=10).*

Materials and Methods (line 487) - Interface partitioning

"To validate that the small system size does not significantly affect our results, we repeated the simulation protocol for a larger system containing 128 protein copies (COM distance: 0–18 nm, harmonic walls: 250 \AA, 10 replicas) for the experimentally validated hnRNPA1-LCD/peptide system, and observed highly similar results (Figure S26)."

And secondly, is the one-bead per residue model accurate enough to find realistic and diverse sequences especially for the water-soluble block. For example, why does this block

contain exclusively charged residues? Considering the effects of counterion condensation, there should be no hardly any difference between 1 charge per amino acid, or 1 per 2. Oligovalines can easily form beta sheet structures.

We acknowledge that the one-bead per residue representation is a simple representation of the condensate system, and we have added a statement to the conclusion (see answer to reviewer point 2.2). The need to simulate hundreds of different peptide sequences leads to prohibitive computational costs when using less coarse-grained models.

As indicated by the reviewer, in the Mpipi force field ions are treated only implicitly via a Debye-Hückel electrostatic screening term, corresponding to a fixed salt concentration of 150 mM. To investigate the effect of charged amino acid content in the water-soluble block, we selected the experimentally validated hnRNPA1-LCD peptide and compared it with a reduced-charge mutant using the more detailed Martini3-IDP force field (Figure R1). In this force field, amino acid side chains, water, as well as salt ions are explicitly represented at the expense of significantly larger computational cost. In the case of the original sequence, there is a preference of the peptide to localize around the interfacial region, while for a mutant where half the lysine residues were mutated to alanines, this behavior is lost (Figure R1). Therefore, this charged tail appears to be an effective driver preventing full recruitment into the dense phase, not only within the implicit-ion Mpipi model, but also in the Martini framework.

New Figure R1 (review only)

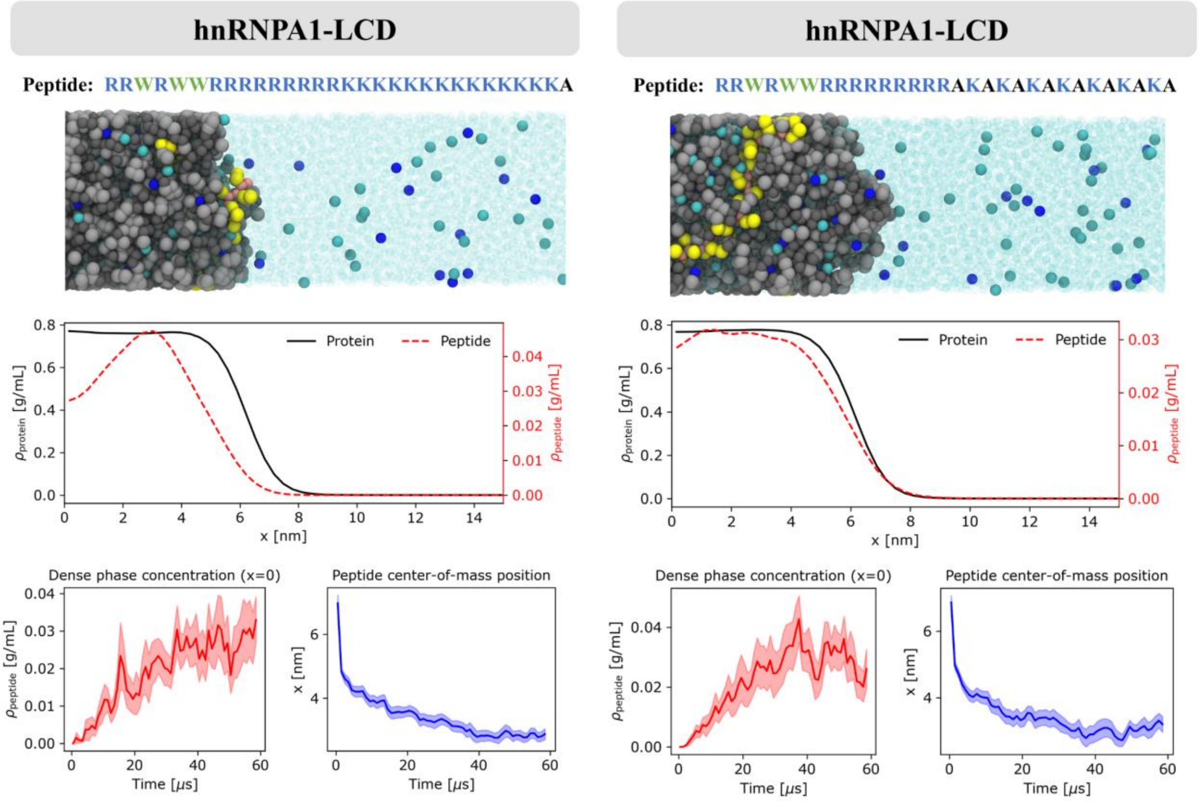

***Figure R1:** Results of Martini3-IDP slab coexistence simulations for two different peptide variants for the hnRNPA1-LCD system. Convergence was assessed from the peptide dense-phase concentration and center-of-mass position. The final 20 us were used to construct density profiles. Uncertainties correspond to standard errors from 16 independent simulations.*

We acknowledge that oligovaline segments can form beta-sheet structures. The experimentally validated peptide containing valine residues passes the three aggregation filters that remove highly aggregation-prone and beta-sheet forming sequences. The proline residues interrupting the valine patches play a key role due to their beta-sheet breaking property.

3. In addition, the machine learning and optimization pipeline contains many choices that are not justified or adequately explained. For example:

Response:

We address each point below and provide further clarification and justification where appropriate.

- What is acceptable statistical error (p. 20)? Compared to what?

During preliminary simulations with hnRNPA1-LCD and peptides, this simulation setup was able to resolve the effects of single amino-acid substitutions involving aromatic residues. We clarified this in the Methods section.

Materials and Methods - Interface partitioning (line 470)

"The small system size and harmonic walls enable faster simulations with acceptable statistical error, for instance allowing resolution of the effects of single amino-acid substitutions that remove or add aromatic residues, as tested in initial trial runs with the hnRNPA1-LCD system."

- How was the equilibration time for the simulations determined (5 ns, 30 ns)?

Equilibration time was estimated based on the deviation of the peptide position from its initial coordinates, as well as relaxation of the total system potential energy. We added 5 examples from the hnRNPA1-LCD initialization to the SI. In principle, the equilibration could be shortened, but because its contribution to total computational cost is already negligible, we kept it fixed at 5 ns to retain a margin for new peptides sampled during optimization.

Materials and Methods - Interface partitioning (line 482)

"Each simulation began with energy minimization and 5 ns of equilibration, allowing the system to relax, as assessed by the deviation from initial coordinates and the total potential energy (Figure S25), followed by a 250 ns production run."

New Figure S25

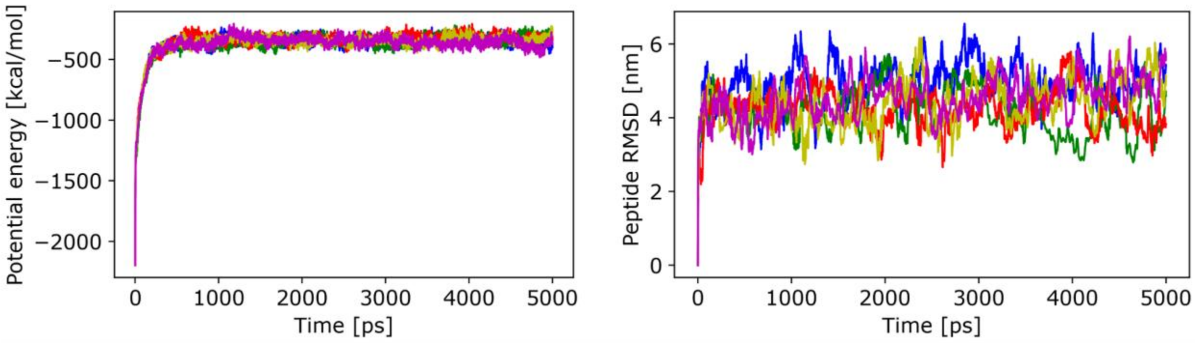

Figure S25: *Equilibration metrics for 5 examples from the hnRNPA1-LCD initialization, based on total potential energy and deviation of peptide position from initial coordinates.*

- How was the set of sequence features chosen?
Next to straight-forward composition features, we encoded the distribution of different residue types using moments of the distribution (Supplementary Information), and included additional features connected to popular sequence features for disordered proteins. Also, the fact that these features can be linearly derived from a one-hot encoded sequence is crucial for the MILP formulation. We summarized these aspects in the main text (line 172): "*We engineered a set of 44 features based on overall composition as well as spatial distribution of charged, aromatic, and strongly self-interacting residues. Additionally, we included features connected to the sequence charge and hydropathy decoration, which play a pivotal role in governing the behavior of disordered proteins.*[ref1,ref2] *It is important to emphasize that all features can be derived as linear transformations of the one-hot encoded amino acid sequence, which was crucial for later steps.*"

We initially constructed this set of 44 features and performed machine learning benchmarks (Figure S1), which showed satisfactory performance of the surrogate models. We did not perform filtering or feature selection starting from a larger feature set. We have clarified this by adding a statement in the Supplementary Information.

Supplementary Information - Featurization of peptide sequences
"These features were not obtained through filtering or feature selection, but were kept as initially defined based on satisfactory performance in initial ML benchmarks (Figure S1)."

- What is the balance between exploration and sampling relevant regions of the design space (p.23). How was this determined?
This parameter was set a priori and not tuned. Exhaustively benchmarking all optimization hyperparameters is difficult due to the high computational cost of the pipeline. We now specify in the main text that tuning the exploit/explore balance could further improve pipeline efficiency.

Results and Discussion - Simulation, machine learning, and optimization setup (line 205)
"The explore/exploit balance as well as the exploration strategy were fixed a priori. Extensive optimization could potentially further increase pipeline efficiency."

- What is the justification for choosing 25 exploration points at iterations 3 and 4, and zero for the last three iterations?
Generally, exploration is most valuable early, when the surrogate model has seen few samples and the design space is poorly covered. In case of this first optimization, we limit computational costs by shifting the focus on exploitation in the last iterations. We clarify this in the Methods section.

Materials and Methods - Machine learning and optimization (line 567)
"In the first optimization with hnRNPA1-LCD as the condensate target, we reduced the number of exploration points to 25 for iterations 3 and 4, and to zero for the last three iterations, fully focusing on the exploitation component to limit computational cost."

- Why are the results of the simulations invariant to sequence flipping due to the coarse-grained representation? (SI p.3)

In the Mpipi one-bead-per-residue coarse-grained model, each amino acid is represented as a single bead, and adjacent beads are connected via a harmonic potential. The bead identity and corresponding interaction parameters depend solely on amino acid type and not on position along the sequence. Because the model does not include an explicit peptide backbone or stereochemistry of side chains, chain directionality is not encoded. As a result, sequences that differ only by sequence flipping are equivalent, and their simulated behavior is identical. We have clarified this point in the Supplementary Information.

Supplementary Information - Featurization of peptide sequences
"It is important to note that the one-bead-per-residue representation in Mpipi does not include an explicit peptide backbone or side-chain stereochemistry, meaning that chain directionality is not encoded. Therefore, the simulations are invariant to sequence flipping."

- Why was the second term intentionally overestimated? (SI p.7). Does this give rise to bias in the sequence?
The volume ratio between dense and dilute phase ϕ = V_dil/V_den, which is part of the objective function, is fixed to 100 for all condensate systems. This is a rough estimate with high uncertainty. We analyzed the relative contributions of both terms in equation (13) (SI p. 7) after the initialization round for hnRNPA1-LCD and found that in the top 5% of sequences according to the interface partitioning objective, the first term makes up 94 ± 5 % (mean ± std, n=30) of the sum. We conclude that minimization of the first term is likely the limiting factor, and therefore perform this slight overestimation of the second term to account for the high uncertainty associated with its prefactor ϕ. This serves as a safeguard against underestimating the true value for ϕ, which would lead to suboptimal designs. Overestimation is less critical, as the second term appears to be easier to minimize. After the approximation leading to (14), the first term still accounts for 93 ± 6 % of the sum for this initialization dataset. While this slightly affects the optimization, we expect the effect to be marginal when compared to biases originating from the uncertainty associated with ϕ or from choosing a specific force field. We added the following clarifications to the Supplementary Information:

Supplementary Information - Objective function interface partitioning
"This corresponds to a rough estimate and therefore carries high uncertainty. Initialization results for the hnRNPA1-LCD system suggested that the first term containing ΔG_1 is more challenging to minimize, as in the top 5 % of initialization sequences the first term accounts for 94 ± 5 % (mean ± std, n=30) of the sum. Given the high uncertainty in the ϕ parameter, we intentionally overestimated the second term containing ΔG_2 as a safeguard against underestimating ϕ, leading to the final objective to be maximized: […] After this approximation, the first term still accounts for 93 ± 6 % of the sum for the top 5 % (measured by p_{int}) of initialization sequences."

- In the second line of equation 13 (SI p.7), the prefactor (V_den/V_int) is apparently set to 1. What is the justification for this?
We do not set V_den/V_int to 1, we multiply the entire objective by V_int/V_den, leading to the final expression in equation 13. Multiplying the objective function by a positive constant leads to an equivalent optimization problem. To clarify this, we add the formula for ϕ in the text:

Supplementary Information - Objective function interface partitioning

“where ϕ=V_dil/V_den denotes the volume ratio of the dilute to dense phases”

4. The manuscript lacks a functional characterization of the synthesized interfacially localizing peptides. The introduction suggests that this pipeline is aimed at targeted modulation of the interface of condensates without perturbing their interior, but the authors have not quantitatively compared the condensate interior properties (e.g., viscosity, density) with and without the peptide; and the surface tension or surface charge with and without the peptide. An alternative proof of targeted modulation could be a suppression of interfacial aggregation in the case of hnRNPA1, or an altered redox activity.

Response:

We agree with the reviewer that a quantitative comparison of the condensate interior as well as surface properties in the presence and absence of the peptides is important to understand its influence in modulating condensate behavior. Following this comment, have we performed additional analyses.

We have already shown that peptide addition leads to a shift in size distribution for all three cases (Figure 4C, Figure S19). In connection with this observation, we also noticed that in the presence of the peptide, droplets more frequently came into contact without fusing, directly indicating altered surface properties. To address this reviewer’s comment, we quantified this by counting the number of contacts within an observation window after 30 minutes of incubation and normalized it to the number of contacts expected from random placement, to deconvolute effects arising from differences in size distribution. We find that these contacts are more abundant when the peptide is present, suggesting inhibited coalescence and changes in the interfacial properties. We have incorporated these findings by adding a new figure S17 as well as corresponding modifications to the main text.

New Figure S17

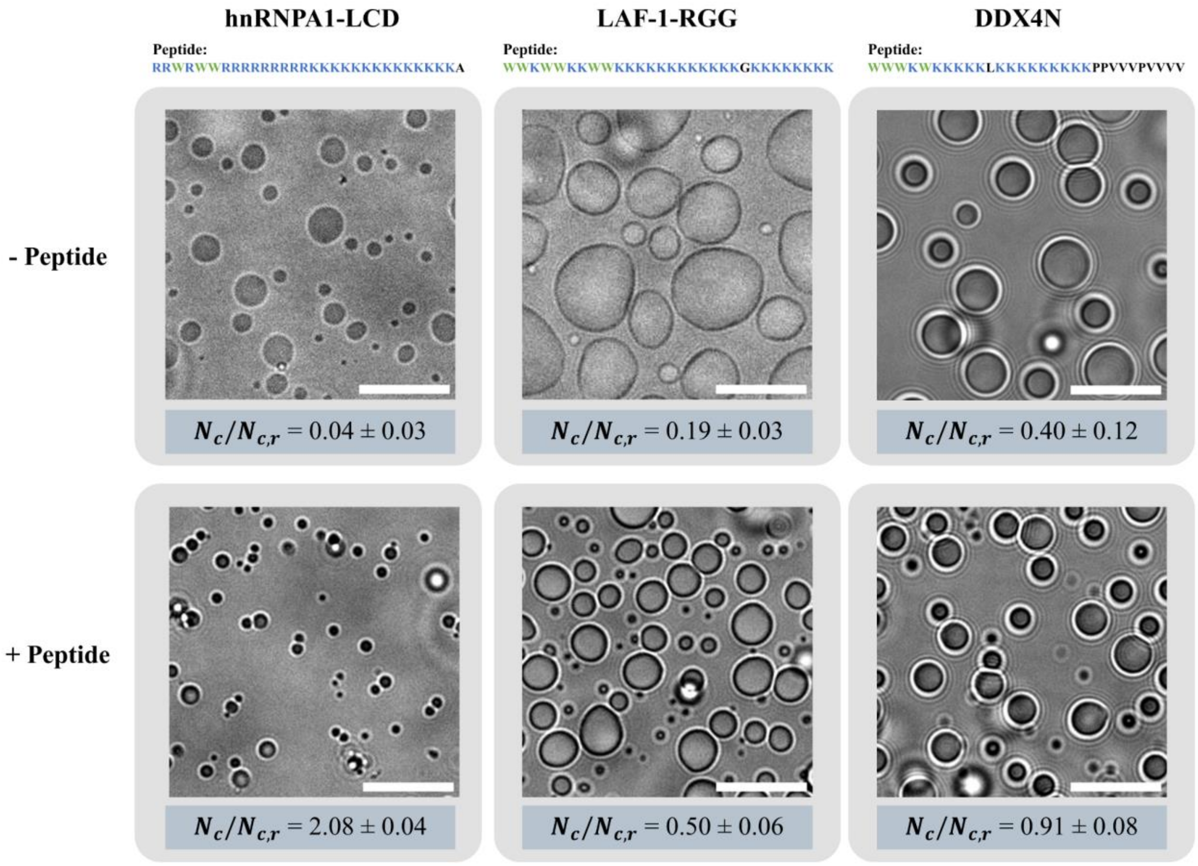

Figure S17: *Analysis of the number of contacts N_c between condensates formed under different conditions after 30 minutes of incubation. To account for differences in size distribution, we normalized by the number of contacts expected from randomly redistributing the condensates within the observation window, $N_{c,r}$. Assuming circular geometry, independent placement, and neglecting border effects, the overlap probability between two condensates is given by $p_c(A,B) = (r_A + r_B)^2\pi/A_{tot}$, leading to an expected number of contacts $N_{c,r} = \pi/A_{tot}\ \Sigma_{i>j}\ (r_i + r_j)^2$. Results show that contacts between condensates with the interface-localizing peptides present are generally more likely, demonstrating inhibited coalescence and therefore altered surface properties. Three windows of 128×128 um^2 were analyzed for each condition (mean ± SD). Scale bar: 20 um.*

Results and Discussion - Peptides targeting the interface of hnRNPA1-LCD condensates (line 292)

"We further analyzed how the interface partitioning of peptide 2 alters the interface and the bulk of the hnRNPA1-LCD condensates. Figure S17 shows the number of contacts between condensates in the absence and presence of peptide 2 after 30 minutes of incubation, demonstrating a higher number of contacts for the sample with peptide 2 and therefore inhibited coalescence, consistent with the shift in size distribution."

Results and Discussion - Designing peptides targeting other protein condensates (line 347)

"Also, coalescence was again inhibited (Figure S17), as observed by condensates more frequently being in contact without fusing…"

Results and Discussion - Designing peptides targeting other protein condensates (line 371)

"...and condensates in contact without fusing were more abundant in the presence of the peptide (Figure S17)."

For the analysis of the effect of the peptides on the bulk of condensates, we quantified the fluorescence lifetime of two different fluorescent molecules (the molecular rotor BODIPY and the dye Atto565-NHS ester) by **Fluorescence Lifetime Imaging Microscopy** (**FLIM**). Fluorescence lifetime is sensitive to the dye's local environment and, for BODIPY specifically, is considered to a certain extent as a reporter of local viscosity. As shown in the new Figure 4D and Figure S20, both dyes show similar lifetime in condensates in the presence and absence of peptide for hnRNPA1-LCD and LAF1-RGG, suggesting a similar chemical environment in the dense phase. However, in the case of DDX4N, the lifetime of both fluorescent probes changed in the presence of the peptide, suggesting an influence of the peptide not only on the interface but also on the properties of the bulk. This observation is likely explained by the lowest predicted preference of the peptide to go to the condensate interface compared to the other condensate systems. We incorporated these findings with the following modifications:

Old Figure 4:

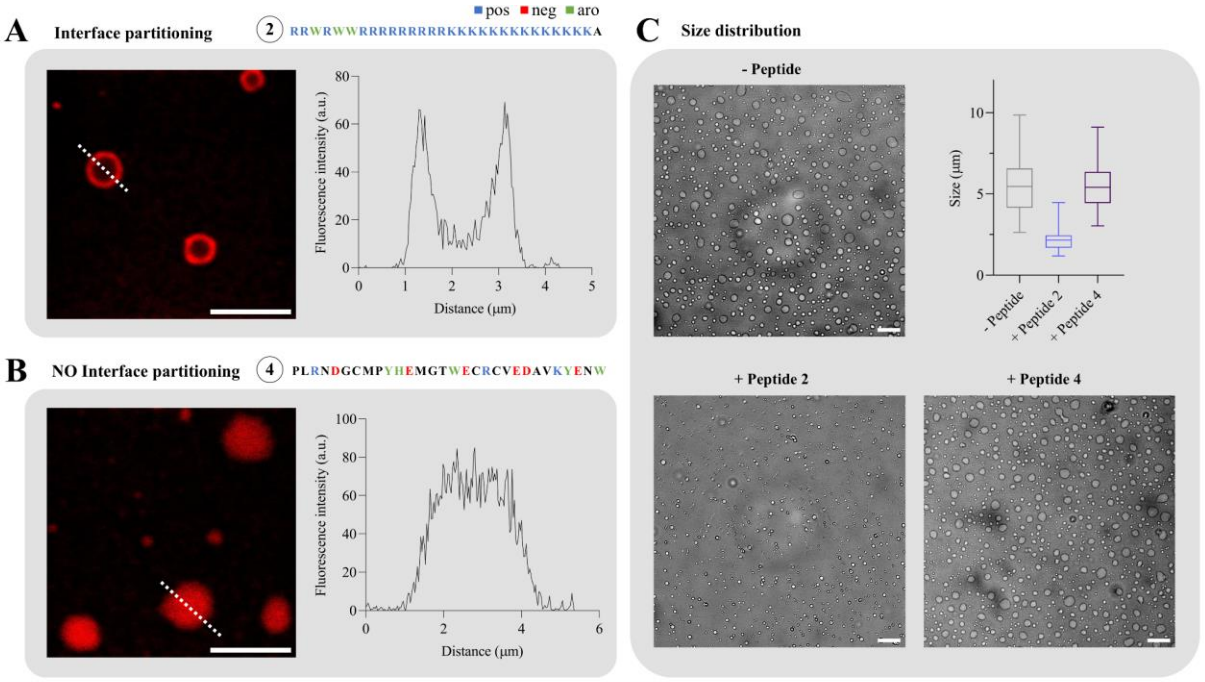

New Figure 4:

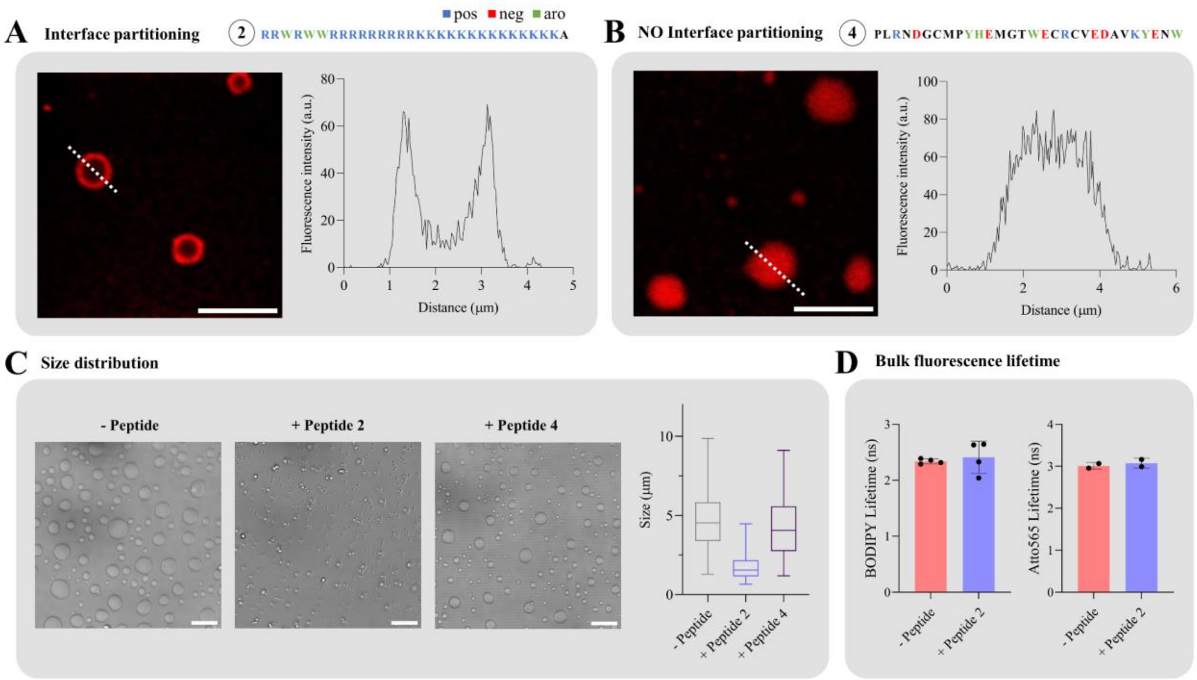

Figure 4: *…(D) Fluorescence lifetime analysis indicates that addition of peptide 2 does not significantly alter condensate bulk properties.*

New Figure S20:

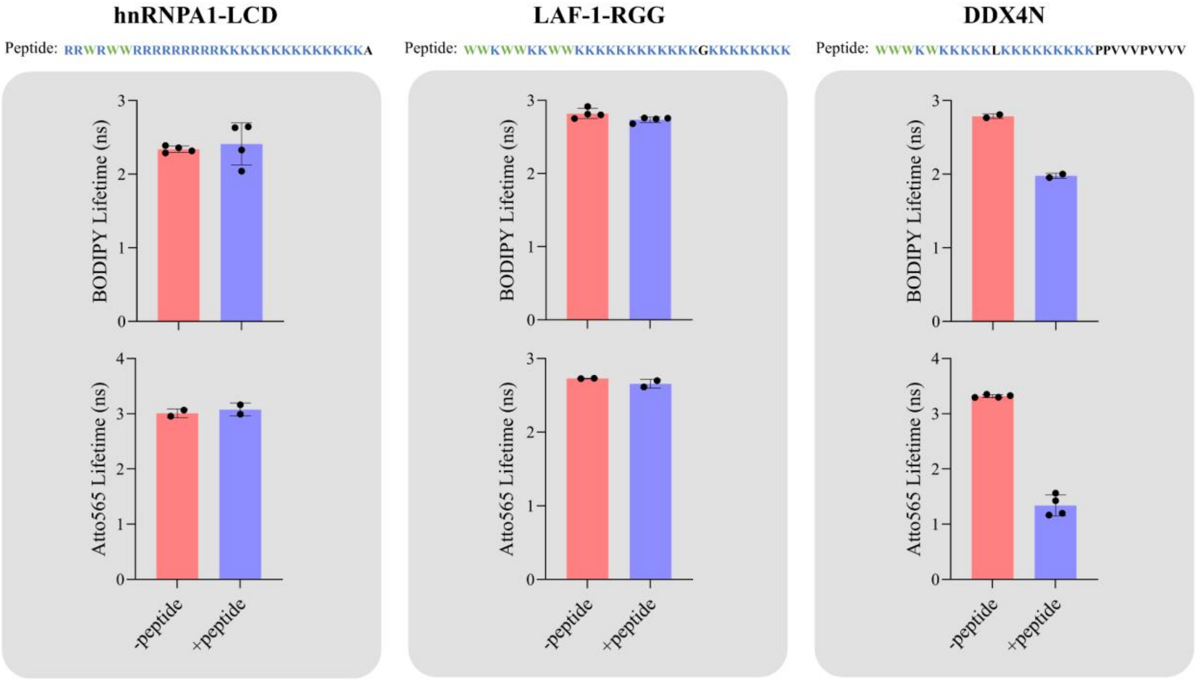

Figure S20: *Impact of interface-partitioning peptides on the fluorescence lifetime of two different dyes in the condensate bulk (mean ± SD). In two out of three cases, the lifetime is unaffected.*

Results and Discussion - Peptides targeting the interface of hnRNPA1-LCD condensates (line 296)

"The properties in the bulk of the condensates were analyzed by quantifying the fluorescence lifetime of two small molecules (Atto-565-NHS ester, and BODIPY) using

Fluorescence Lifetime Imaging Microscopy (FLIM). Fluorescence lifetime is sensitive to the dye's local environment and, for BODIPY specifically, is associated with local viscosity[ref]. As shown in Figure 4D, the lifetime of both molecules in the interior of the condensates does not change upon peptide addition, suggesting a negligible effect of the peptide on the bulk."

Results and Discussion - Designing peptides targeting other protein condensates (line 347)
"...and addition of the peptide did not affect fluorescence lifetime of Atto565 and BODIPY in the bulk (Figure S20)."

Results and Discussion - Designing peptides targeting other protein condensates (line 372)
"In this case, however, the fluorescence lifetime of Atto565 and BODIPY appeared altered (Figure S20), suggesting that the peptide also affects bulk properties to some extent. While the peptide still shows a preference for the interface, its partial impact on bulk properties is consistent with the lowest hypervolume progress and the lowest predicted interface partitioning among the three optimization cases."

Finally, we would like to emphasize that the primary goal of this work is the computational design of interface-localizing peptides, combined with experimental validation of the pipeline and analysis of key sequence features. Although a deeper investigation of how these peptides alter condensate properties is certainly interesting and relevant, it lies beyond the scope of this study. The broader consequences of interface partitioning will be an exciting direction for future work.

Minor:
5. "Moreover, a high-throughput experimental strategy for extensive screening is still lacking." (p.4). This manuscript does not provide a high-throughput experimental strategy.
Response:
We did not intend to claim that we developed a high-throughput experimental strategy. Our point was that, as no established high-throughput method currently exists, we cannot rely on one to design interface-localizing peptides. To clarify this, we modify the statement in the main text, making it more general:

Introduction (line 63)
"Moreover, a high-throughput experimental or computational strategy for extensive screening is still lacking."

6. Line 73 and 75: "dynamics" is missing after "molecular" (2x)
Response:
As indicated by the reviewer, we replaced the more general term "molecular simulations" with "molecular dynamics simulations" at the indicated positions.

7. Figure 4c and Figure S12: what is the blurry dark ring in the center of the field of view in the brightfield images?
Response:
The blurry dark ring is an imaging artifact, likely originating from the camera lens. This is supported by its appearance across different conditions.

We acquired new images using confocal microscopy, resulting in improved image quality, using newly prepared protein and peptide batches. We replaced the bright field microscopy images in Figure 4C with confocal microscopy images and moved the bright field images to the Supporting Information (Figure S14). We also updated the size distribution data for hnRNPA1-LCD by combining the previous bright field data with the new confocal data, recovering the same trend:

Updated Figure 4:

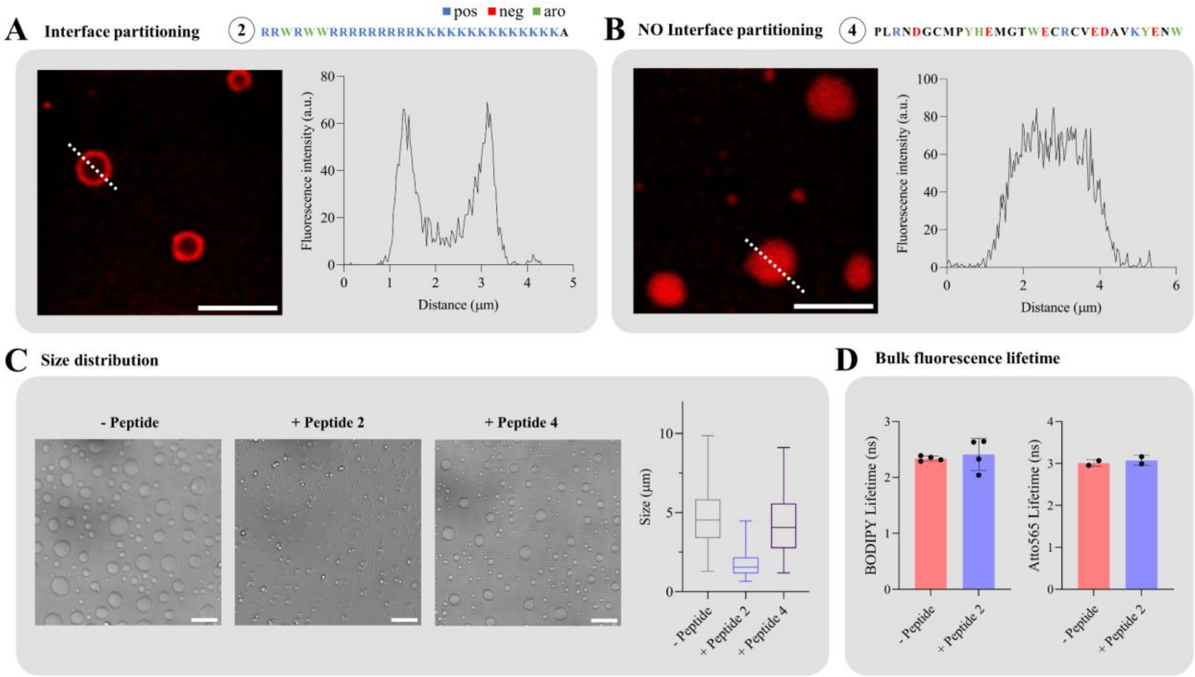

Figure 4: *…(C) Confocal microscopy images and corresponding size distributions (n=50), which were evaluated also considering bright field microscopy images acquired using a different protein and peptide batch (Figure S14, n=50). Addition of peptide 2 decreased the size of the condensate, while addition of peptide 4 had a negligible impact. Scale bar: 10 um…*

New Figure S14:

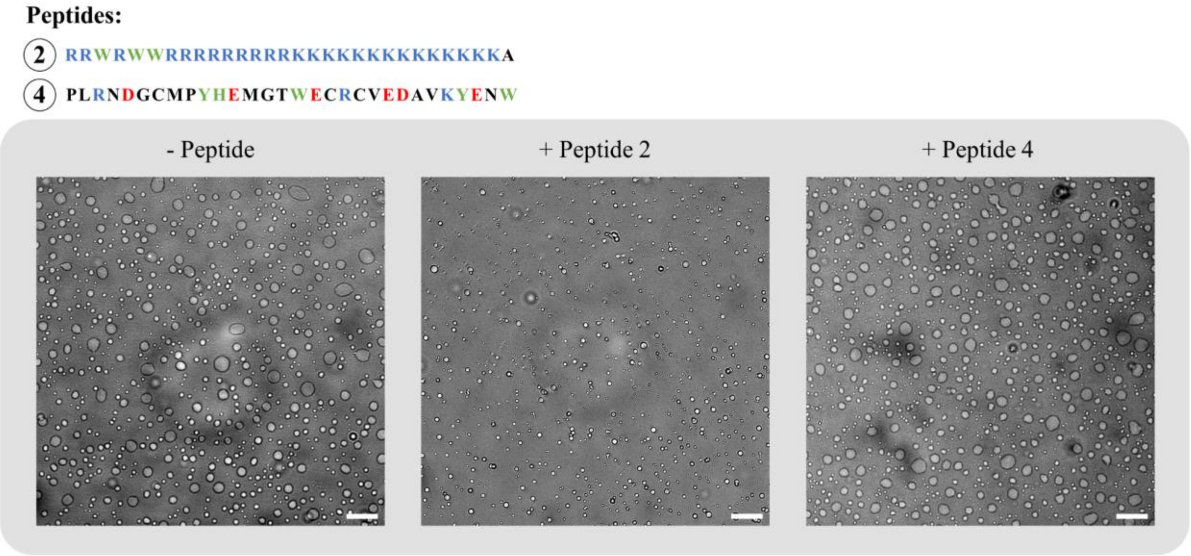

Figure S14: *Bright field microscopy images of hnRNPA1-LCD condensates in the absence and presence of peptide 2 or peptide 4. Scale bar: 20 um.*

Results and Discussion - Peptides targeting the interface of hnRNPA1-LCD condensates (line 280)

"Interestingly, consistent with its interfacial localization, peptide 2 also caused a significant decrease in average condensate diameter (from 4.7 ± 1.8 um to 1.7 ± 0.8 um, mean ± standard deviation, n = 100), as analyzed by confocal microscopy and bright field microscopy (Figure 4C, Figure S14)." [...] "The addition of control peptide 4 did not substantially affect the condensate diameter, which remained nearly unchanged at 4.2 ± 1.8 um."

8. Figure 5d and Figure S8: why do not all condensates exhibit complete surface coverage? Are the systems not in equilibrium?

Response:

The fact that interfacial localization does not change over time (Figure S13) indicates that the system has reached equilibrium. As discussed in the answer to reviewer point 1.5, we designed the peptides in the low-concentration limit and do not explicitly model interface partitioning behavior at higher concentrations (only implicitly by limiting homotypic interactions), which could be a valid extension of the design pipeline in future studies. At the tested experimental concentrations, we assume that adsorption does not form a single peptide monolayer. Confocal microscopy suggests that there might be multilayer adsorption, or formation of a second phase wetting the condensate interface, which could explain the "patchy" behavior observed in Figure 5d and Figure S18. Regardless, interfacial localization in the low-concentration limit appears to correlate with finite-concentration behavior, as supported by experimental validation. We modified the manuscript to incorporate these points:

Results and Discussion - Designing peptides targeting other protein condensates (line 363)

"For DDX4N, and to some extent also for the other systems, interfacial localization is not perfectly uniform and results in multilayer adsorption or formation of a second phase wetting

the condensates. This cannot be fully captured by our simulation pipeline, which optimizes adsorption in the low-concentration limit and only implicitly accounts for finite-concentration behavior by limiting homotypic peptide interactions. Nevertheless, the enrichment of peptides at the interface indicates that low-concentration design correlates with finite-concentration behavior and supports the applicability of our designs in this regime."

Conclusions (line 445)

We added a comment on extending the design pipeline to finite concentrations (see also answer to reviewer point 1.5)

9. Figure S10: should the labels be inverted? I would expect the condensates with the peptide to have a smaller average size.

Response:

The labels are correct. Addition of the interface-partitioning peptide decreases the average condensate size observed by brightfield microscopy. Assuming that the total dense phase volume remains approximately constant (a reasonable assumption given the 10:1 protein-to-peptide molar ratio), this results in a higher abundance of smaller (nano)condensates. We modified the main text to clarify this point:

Results and Discussion - Peptides targeting the interface of hnRNPA1-LCD condensates (line 283)

"Dynamic light scattering analysis indicated that peptide 2 also promoted formation of nanometer-sized clusters which are below the limit of detection of microscopy, indicating a global shift in the size distribution toward smaller values (Figure S15)."

Revisions - Nature Communications

De novo design of peptides localizing at the interface of biomolecular condensates

Reviewer #1 (Remarks to the Author):

In the revised manuscript, Schneider et al. have largely addressed the concerns raised previously. Using dye labelled peptides and FCS analyses, the authors have shown that identified peptide sequences have poor homotypic interactions at evaluated concentrations, making them distinct from typical surfactant architectures.

Using a higher resolution CG FF such as MARTINI3-IDP, the authors have validated some of the previously identified molecular designs for interface localization and demonstrated reasonable agreement with the one-bead-per-residue models. One minor concern I have is regarding response number 3 - "In the case of Martini3-IDP, interfacial localization was clearly confirmed for two peptides." The density profiles reported in Figure S23, appear to show only for the DDX4N case that the peptide is partly in the protein-rich as well as water-rich phases. In the other two, the peptide appears to be sequestered away from the interface. Which two peptides are the authors referring to here? Additionally, by evaluating simulations with varying number of peptide chains, the authors demonstrate that the distribution of condensate residues at the interface is not a given upon localization. In light of these additional simulations, experiments and analyses, I recommend the manuscript for publication.
Response: We sincerely thank once more this reviewer, as well as the other two reviewers, for their thorough, constructive comments, which have substantially improved the manuscript. Their efforts are much appreciated. We clarified the minor concern in the main text (line 390): *"In the case of Martini3-IDP, preference for the interfacial region over the condensate bulk was confirmed for the DDX4N and hnRNPA1-LCD systems, although in the latter case the peptide mainly localized just beneath the condensate interface."*

Reviewer #2 (Remarks to the Author):

The authors have made an exceptional effort addressing all reviewers concerns and criticism. Of particular note the additional experimental validation and further simulation efforts that apply force fields, CALVADOS 2 and MARTINI3 IDP, to address any concerns of excessive biasing.
Response: We sincerely thank once more this reviewer, as well as the other two reviewers, for their thorough, constructive comments, which have substantially improved the manuscript. Their efforts are much appreciated.

Reviewer #3 (Remarks to the Author):

The authors have done a commendable job to address all the points raised by the reviewers with new experiments, detailed explanations and significant reorganization of their

manuscript. I deeply appreciate the efforts that the authors made and the seriousness with which they have addressed most of the points. The authors' responses and revisions in the manuscript have addressed the most important concerns adequately, and I would be happy to recommend the manuscript for publication if the authors could take a look at a few remaining points of unclarity.

Response: We sincerely thank once more this reviewer, as well as the other two reviewers, for their thorough, constructive comments, which have substantially improved the manuscript. Their efforts are much appreciated. We address the remaining points raised by the reviewer below.

- Regarding FCS experiments and the authors' response to Point 1. I appreciate the authors' efforts to perform these FCS experiments. However, interpreting FCS data can be complicated, and small fractions of larger clusters are easily overlooked because of the small contribution to the autocorrelation function. If the authors choose to present these data, they should at least show the exponential fit and residuals. From the residuals it is more straightforward to assess the potential presence or absence of larger assemblies. I may be wrong, but it seems that there is a slight "long tail" in the G-1 data starting around 10^0 ms, which could indicate a small fraction of larger clusters. Moreover, of the optimized interfacial peptide sequences, the peptide designs for LAF-1 and Ddx4 condensates seem the most prone to self-assembly in the absence of condensates, but these were not tested by FCS. Related to this point, the authors may want to nuance their statement about "This observation confirms that homotypic peptide interactions are minimized, as no larger assemblies are produced by the peptide alone"

Response: As suggested by the reviewer, we plotted the data with the corresponding exponential fit and residuals, for each replicate separately. The small peak at 10^0 ms can be attributed to measurement noise since it is only present in one single replica.

We also adapted the statement pointed out by the reviewer (line 288): *"This observation confirms that homotypic peptide interactions are minimized for this specific example, as no larger assemblies are produced by the peptide alone."*

Old Figure S16:

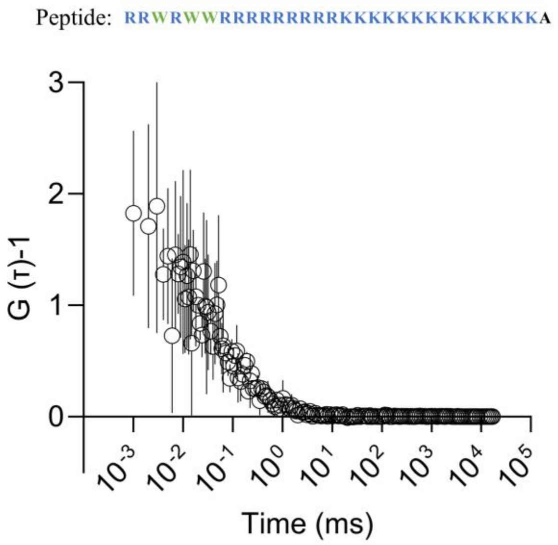

New Figure S16:

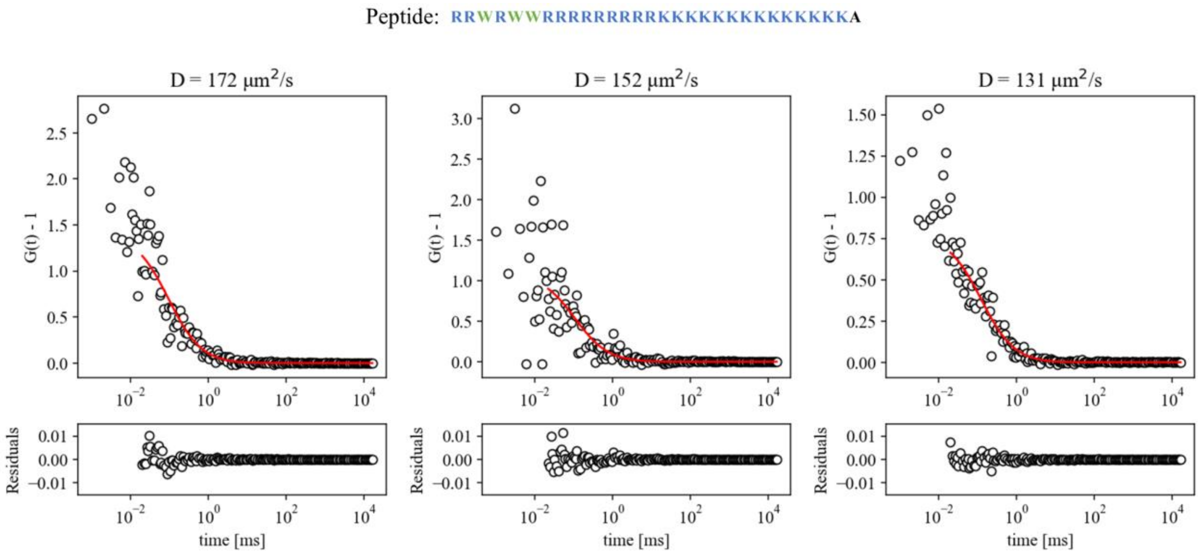

Figure S16: *FCS results (3 replicas) of the peptide 2 alone (1 uM, 50 % labeled) in 20 mM Tris (pH 7.5) and 150 mM NaCl. The fitted diffusion coefficient was 152 ± 20 um²/s, corresponding to a hydrodynamic radius of 1.41 +- 0.19 nm calculated using the Stokes–Einstein equation (mean ± SD, n = 3). The theoretical radius of gyration of a monomer, assuming a C-α separation distance of 0.381 nm, is estimated to be between 0.9 nm (ideal chain: R_g = N b/sqrt{6}) and 3.3 nm (rod: R_g = N b/sqrt{12}).*

- Regarding the new Figure S13, and the conclusion that interfacial localization does not change over time: this figure should be accompanied by an analysis of the (unadjusted) intensity profiles across the condensate droplets. It is difficult to assess the change in localization from only the image where brightness and contrast settings may differ between experiments. From these images it appears that the peptide actually does leave the interface slowly to dissolve in the surrounding dilute phase, but I may be wrong.

Response: Prompted by the comment of the reviewer, we have added the intensity profiles into the corresponding figures, showing that the peptide remains preferentially localized at the interface over time, as intended by design, and that this localization is not due to slow diffusion inside the dense phase. As noted by the reviewer, there is an increase of the peptide concentration in the dilute phase relative to the dense phase over time. This could be due to an increase of the clusters in the size range 500-1000 nm (DLS data, Figure S15) over time, related for instance to an emulsification process similar to the one observed in Tand D. et al., Nat. Chem. 2025 (https://doi.org/10.1038/s41557-025-01800-4) or to an alternative aging process.

Old Figure S13:

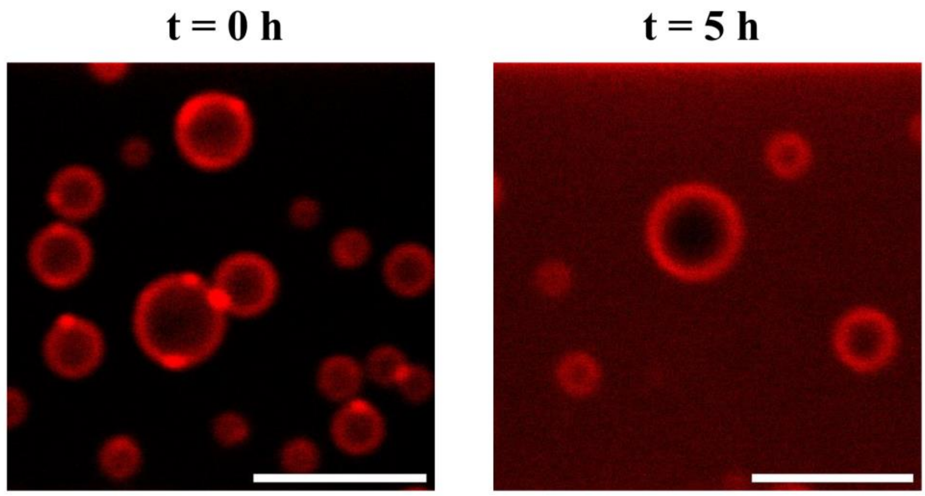

New Figure S13:

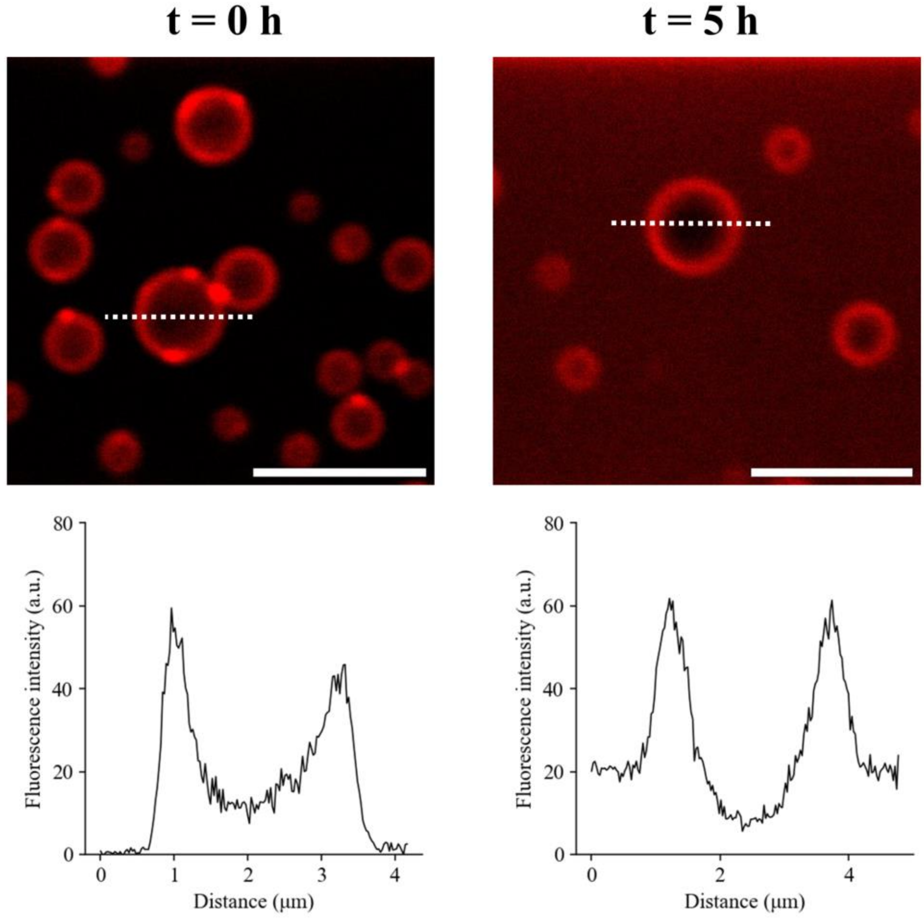

Figure S13: *Interfacial localization of designed peptide at the condensate formed by hnRNPA1-LCD (10 uM protein/peptide, 20 mM Tris (pH 7.5) and 150 mM NaCl) is consistent over 5 hours of incubation. Scale bar: 5 um.*

- Regarding the new Figure S15, and the authors' response to Point 3.9. I do not agree with the authors' conclusion, and also find this figure confusing. "Assuming that the total dense phase volume remains approximately constant (a reasonable assumption given the 10:1

protein-to-peptide molar ratio), this results in a higher abundance of smaller (nano)condensates.” If microscopy shows that the average size of micron-sized condensates becomes smaller and the total dense phase volume remains constant, this does not say anything about the abundance of nanocondensates. The total number of (smaller) micron-sized condensates may simply be larger, and the number and size of the nanocondensates remains the same. Moreover, Figure S15 does not show that there is a higher abundance of nanocondensates: the distributions are normalized, and only show an increase in the average size of the nanocondensates with addition of peptides. Why is that expected? Since there is no information about the relative amounts of nanocondensates and micron-sized condensates, it cannot be concluded that “indicating a global shift in the size distribution toward smaller values (Figure S15)”.

Response: We appreciated that this point required clarification. In the presence of the peptide, the DLS data indicates the presence of nanocondensates in the size range 500–1000 nm, which in the absence of peptide would otherwise coarsen and grow into larger micron-scale condensates. This is consistent with the significant decrease in average condensate diameter observed in the micron scale. Since nanometer-sized clusters are present also without peptide, we appreciated that the sentences “*promoted formation of nanometer-sized clusters*” and “*indicating a global shift in the size distribution toward smaller values*” were confusing. We rephrased (line 284): “*promoted formation of clusters in the size range 500-1000 nm, below the limit of detection of microscopy (Figure S15), which would grow into larger micron-size condensates in the absence of the peptide. We further confirmed that these clusters did not form in the absence of the protein, as shown by fluorescence correlation spectroscopy (FCS) (Figure S16).*”
